# New functions of pirin proteins and a 2‐ketoglutarate: Ferredoxin oxidoreductase ortholog in *Bacteroides fragilis* metabolism and their impact on antimicrobial susceptibility to metronidazole and amixicile

**DOI:** 10.1002/mbo3.1429

**Published:** 2024-08-07

**Authors:** Andrea M. Gough, Anita C. Parker, Patricia J. O'Bryan, Terence R. Whitehead, Sourav Roy, Brandon L. Garcia, Paul S. Hoffman, C. Jeffrey Smith, Edson R. Rocha

**Affiliations:** ^1^ Department of Microbiology and Immunology Brody School of Medicine at East Carolina University Greenville North Carolina USA; ^2^ USDA Agricultural Research Service Peoria Illinois USA; ^3^ Department of Medicine, Division of Infectious Diseases and International Health University of Virginia Charlottesville Virginia USA

**Keywords:** amixicile, anaerobic bacteria, antimicrobial, *B. fragilis*, metronidazole, pirin‐protein interactions

## Abstract

The understanding of how central metabolism and fermentation pathways regulate antimicrobial susceptibility in the anaerobic pathogen *Bacteroides fragilis* is still incomplete. Our study reveals that *B. fragilis* encodes two iron‐dependent, redox‐sensitive regulatory pirin protein genes, *pir1* and *pir2*. The mRNA expression of these genes increases when exposed to oxygen and during growth in iron‐limiting conditions. These proteins, Pir1 and Pir2, influence the production of short‐chain fatty acids and modify the susceptibility to metronidazole and amixicile, a new inhibitor of pyruvate: ferredoxin oxidoreductase in anaerobes. We have demonstrated that Pir1 and Pir2 interact directly with this oxidoreductase, as confirmed by two‐hybrid system assays. Furthermore, structural analysis using AlphaFold2 predicts that Pir1 and Pir2 interact stably with several central metabolism enzymes, including the 2‐ketoglutarate:ferredoxin oxidoreductases Kor1AB and Kor2CDAEBG. We used a series of metabolic mutants and electron transport chain inhibitors to demonstrate the extensive impact of bacterial metabolism on metronidazole and amixicile susceptibility. We also show that amixicile is an effective antimicrobial against *B. fragilis* in an experimental model of intra‐abdominal infection. Our investigation led to the discovery that the *kor2AEBG* genes are essential for growth and have dual functions, including the formation of 2‐ketoglutarate via the reverse TCA cycle. However, the metabolic activity that bypasses the function of Kor2AEBG following the addition of phospholipids or fatty acids remains undefined. Overall, our study provides new insights into the central metabolism of *B. fragilis* and its regulation by pirin proteins, which could be exploited for the development of new narrow‐spectrum antimicrobials in the future.

## Graphical abstract

This study reveals that several enzymes involved in the central metabolism of *Bacteroides fragilis* are regulated by protein‐protein interactions with pirin proteins. We observed changes in susceptibility to the antimicrobials metronidazole and amixicile in various metabolic mutants. Amixicile, a novel inhibitor that binds to thiamine^−^diphosphate dependent enzymes, has proven effective in eliminating *B. fragilis* in a model of intra‐abdominal infection. Furthermore, we identified a 2‐ketoacid: ferredoxin oxidoreductase as essential for growth and proposed its multifunctional roles.

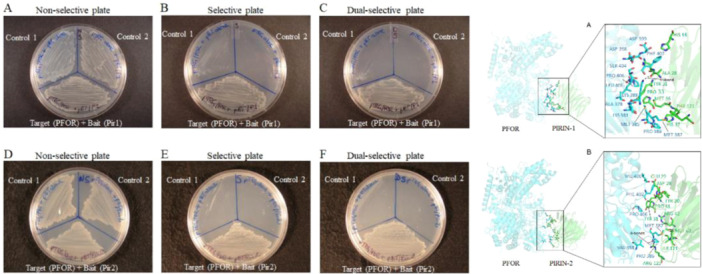

## 1 INTRODUCTION

Among the anaerobic pathogenic bacteria causing human infections, *Bacteroides fragilis* is the most frequent isolate, and multi‐drug‐resistant strains are on the rise accounting for most treatment failures (Byun et al., 2019; Hartmeyer et al., 2012; Jasemi et al., 2021; Nagy et al., 2011; Schuetz, 2014; Snydman et al., 2017). Metronidazole (MTZ) remains the antibiotic of choice for the management of infections caused by anaerobes and resistance to MTZ is generally still low, however, resistant strains have been reported in regional survey studies (Alauzet et al., 2019; Hartmeyer et al., 2012; Jasemi et al., 2021; Nagy et al., 2011; Shafquat et al., 2019; Shilnikova & Dmitrieva, 2015). MTZ is a derivative of the 5‐nitroimidazole class of prodrugs requiring [(2 pairs of 2e^−^) or 4e^‐^] reductions of the nitro group for activation to produce a reactive radical species accountable for its lethal DNA mutagenic and strand fragmentation activity (Alauzet et al., 2019; Dingsdag & Hunter, 2018; Ghotaslou et al., 2018; Sisson et al., 2000). Therefore, given the continuous increase in *B. fragilis* multi‐drug resistance (MDR) traits and the steady decrease in the number of new alternative antibiotics, there is a need to find alternative anaerobic therapeutics. In this regard, recent studies have reported on a novel antimicrobial, amixicile (AMIX), that exhibits excellent in vitro and in vivo activity against oral anaerobic bacterial pathogens grown on biofilm or internalized by host cells (Gui et al., 2019; Gui et al., 2020; Gui et al., 2021; Hutcherson et al., 2017; Reed et al., 2018).

Amixicile, a novel second generation of nitazoxanide with improved bioavailability and selectivity (Hoffman, 2020), inhibits the action of thiamine‐diphosphate (ThPP) cofactor in pyruvate: ferredoxin oxidoreductase (PFOR) and related members of the 2‐ketoacid ferredoxin oxidoreductase (KFOR) superfamily found in obligate anaerobic bacteria, anaerobic human intestinal parasites, and in members of the epsilon proteobacteria such as *Campylobacter* and *Helicobacter* (Hoffman et al., 2014; Hoffman, 2020; Kennedy et al., 2016; Warren et al., 2012). Unlike MTZ, AMIX and NTZ do not undergo redox electron transfer, they are not mutagenic (Ballard et al., 2011; Hoffman et al., 2007; Warren et al., 2012), and do not exhibit cross‐resistance with MTZ in tested organisms (Hoffman et al., 2007). Also important, AMIX targets are not found in humans, mitochondria or in aerobic and facultative anaerobes which utilize pyruvate dehydrogenase (PDH) in the catabolism of pyruvate (Hoffman, 2020). Preclinical studies show that AMIX is effective in treating *Clostridioides difficile* colitis, *Helicobacter pylori* gastritis and periodontitis in animal models (Gui et al., 2021; Hoffman et al., 2014; Warren et al., 2012). More specifically, AMIX inhibits the growth of the “Red complex” of oral anaerobic pathogens (Gui et al., 2019; Gui et al., 2020; Gui et al., 2021; Hutcherson et al., 2017; Reed et al., 2018) and the anaerobic protozoan *Trichomonas vaginalis* (Jain et al., 2022). Pharmacokinetic studies indicate that AMIX is efficiently absorbed (bioavailability), does not concentrate in or alter the intestinal microbiota of healthy mice and is eliminated via the renal system (Hoffman et al., 2014). However, very little is known about AMIX activity against *B. fragilis*.

PFOR is a major metabolic enzyme in the oxidative decarboxylation of the pyruvate pathway in anaerobes and the best‐studied member of the KFOR superfamily (Gibson et al., 2016; Ragsdale, 2003). However, PFOR is not essential for *B. fragilis* growth in vitro, and though PFOR is a major player in MTZ activation and a target for AMIX activity, the lack of PFOR adds a modest increase in resistance to MTZ (Diniz et al., 2004) and does not abolish susceptibility to AMIX (this study). This indicates that other metabolic pathways containing ThPP‐binding enzymes that catalyze the cleavage or formation of carbon‐carbon bonds of 2‐ketoacids or 2‐hydroxyketones (Gibson et al., 2016; Prajapati et al., 2022) may play important roles in MTZ and AMIX susceptibility. Nearly all ThPP‐dependent enzymes can perform one‐electron redox reaction steps that occur in the 2‐electron process in oxidoreductases, and the low potential (~ −500mV) electrons generated can reductively activate pro‐drugs such as MTZ (Chen et al., 2019; Gibson et al., 2016; Reed et al., 2012).

In addition to PFOR, *B. fragilis* encodes three other ThPP‐binding KFOR members: two 2‐ketoglutarate:ferredoxin oxidoreductases (KGOR); the *kor1AB* (BF638R_4321‐4322) and the *kor2ABG* genes encompassed in the *kor2CDABEG* putative operon (BF638R_1660‐1655), and the indolepyruvate ferredoxin oxidoreductase, *iorAB* (BF638R_1606‐1605). *B. fragilis* Kor1AB and Kor2ABG subunits are orthologs to *Magnetococcus marinus MmOGOR*, KorAB (Chen et al., 2019), *Hydrogenobacter thermophilus HtOGORs*, KorAB, and ForDABGE (Yamamoto et al., 2006; Yamamoto et al., 2010; Yun et al., 2001; Yun et al., 2002), KGOR of *Thermococcus litoralis* α‐ and β‐subunits (Mai and Adams, 1996), and KGOR of *Tharnea aromatica* KorAB (Dörner & Boll, 2002) which catalyze the reductive carboxylation of succinyl‐CoA to form 2‐ketoglutarate (2‐KG) in the reverse (reductive) TCA cycle. However, there is a paucity of information regarding the contribution of Kor1AB and Kor2CDAEBG in *B. fragilis* reverse reductive TCA cycle mode.

Interestingly, several studies have demonstrated that *B. fragilis* has a bifurcated TCA cycle containing the heme‐dependent reductive branch oxaloacetate to succinate pathway, and the heme‐independent citrate/isocitrate to 2‐KG oxidative pathway branch but lacking reductive synthesis of 2‐KG via succinate (Baughn & Malamy, 2002; Baughn & Malamy, 2003; Chen & Wolin, 1981; Harris & Reddy, 1977; Macy et al., 1975; Macy et al., 1978). However, there is strong evidence that *B. fragilis* and other anaerobic bacteria synthesize 2‐KG from succinate via reductive carboxylation of succinyl‐CoA. Using [1,4‐^14^C]‐succinate, or D‐[U‐^14^C]‐glucose it was demonstrated that radiolabeled carbon was incorporated into glutamate via succinate, but not via the citrate/isocitrate pathway (Allison & Robinson, 1970; Allison et al., 1979). In the closely related organism *Bacteroides thetaiotaomicron*, metabolomic studies monitoring carbon flux with radiolabeled [U‐^13^C]‐glucose also showed that glutamate and its precursor 2‐KG are synthesized via succinate but not via citrate (Schofield et al., 2018). Assimilation of [U‐^13^C]‐acetate in *B. thetaiotaomicron* results in the propagation of labeled citrate, but not to glutamate indicating that 2‐KG is not formed by the oxidative branch (Schofield et al., 2018), though it is assumed to occur in *B. fragilis* under heme‐limiting conditions (Baughn & Malamy, 2002). Nonetheless, no genetic evidence nor enzymatic activity responsible for the reductive formation of 2‐KG in *B. fragilis* has been described. In this regard, we have initiated studies to understand the regulation and metabolic role of Kor1AB and Kor2CDAEBG in *B. fragilis* central metabolism.

Pirins are highly conserved redox‐sensitive, iron‐binding proteins belonging to the functionally diverse cupin protein superfamily. Pirins contribute to the control and modulation of a diverse range of regulatory and metabolic activities in archaea, prokaryotes and eukaryotes. (Agarwal et al., 2009; An et al., 2004; Dunwell, et al., 2000; Dunwell et al., 2001; Dunwell, et al., 2004; Hihara et al., 2004; Lapik & Kaufman, 2003; Orzaez et al., 2001; Pang et al., 2004; Yoshikawa et al., 2004; Wendler et al., 1997). In the bacterium *Serratia marcescens*, pirin interacts with the pyruvate dehydrogenase (PDH) E1 subunit and modulates the PDH activity determining the direction of the pyruvate metabolism toward the TCA cycle or the fermentation pathway (Soo et al., 2007). The involvement of pirin in modulating the central metabolism of prokaryotes was also demonstrated in *Streptomyces ambofaciens* (Talà et al., 2018) and *Aliivibrio salmonicida* (Hansen et al., 2012). In *Acinetobacter baumanii*, pirin regulates the expression of adaptative efflux‐mediated antibiotic resistance (Young et al., 2023). In *Bacteroides thetaiotaomicron,* recombinant pirin exerts immunomodulating actions preventing the induction of pro‐inflammatory interleukins in animal models of Crohn's disease (Delday et al., 2019).

In this study, we show that *B. fragili's* central metabolism activities are controlled, at least partially, by pirin proteins. Using a series of metabolic mutant strains and electron transport inhibitors, we show that resistance to MTZ involves a complex accumulation of mutations affecting metabolic pathways and electron transfer in energy conservation mechanisms. In addition, we carry out experiments to understand the metabolic role of Kor1AB and Kor2CDAEBG in *B. fragilis* central metabolism and show evidence that *kor2AEBG* genes are essential for growth and may play a role in the formation of 2‐KG. Lastly, we demonstrate the novel ThPP‐dependent enzyme cofactor inhibitor AMIX has antimicrobial activity against *B. fragilis* in an experimental model of intra‐abdominal infection.

## 2 MATERIALS AND METHODS

### 2.1 Strains and growth conditions

The bacterial strains and plasmids used in this study are listed in Table 1. *Bacteroides* species Strains were routinely grown on BHIS (brain heart infusion) supplemented with l‐cysteine (1 g/L), hemin (5 mg/L), and NaHCO_3_ (20 mL of a 10% solution per litre). A chemically defined medium was formulated as follows: KH_2_PO_4_ (1.15 g/L); (NH4)_2_SO_4_ (0.4 g/L); NaCl (0.9 g/L); l‐methionine (75 mg/L); MgCl_2_.6H_2_O (20 mg/L); CaCl_2_.2H_2_O (6.6 mg/L); MnCl_2_.4H_2_O (1 mg/L); CoCl_2_.6H_2_O (1 mg/L); resazurin (1 mg/L); l‐cysteine (1 g/L); hemin (5 mg/L); and d‐glucose (5 g/L) or otherwise stated in the text. The final pH was 6.9. Rifampin (20 μg/mL), gentamicin (100 μg/mL), erythromycin (10 μg/mL), tetracycline (5 μg/mL), cefoxitin (25 μg/ml), 5‐fluor‐2'‐deoxyuridine, FUdR, (200 μg/mL), or anhydrotetracycline, aTC (100 ng/mL) were added to the media when required. *E. coli* strains were routinely grown on lysogeny broth media with appropriate antibiotics.

**Table 1:** Bacterial strains and plasmids used in this study.

Strains	Genotype	References
*B. fragilis* 638R	clinical isolate, Rif^r^	Privitera et al. (1979)
BER‐2	638R *ΔfurA::cfxA*, Rif^r^ Cfx^r^	Robertson et al. (2006)
BER‐63	638R *ΔftnA::tetQ*, Rif^r^ Tet^r^	Gauss et al. (2012)
BER‐74	638R *Δbfr::cfxA*, Rif^r^ Cfx^r^	Gauss et al. (2012)
BER‐75	638R *ΔftnA::tetQ Δbfr::cfxA*, Rif^r^ Tet^r^ Cfx^r^	Gauss et al. (2012)
BER‐150	638R *ΔfnrA::tetQ*, Rif^r^ Tet^r^	This study
BER‐156	638R *ΔnrfA::tetQ*, Rif^r^ Tet^r^	Lab strain
BER‐164	638R *Δpir1::tetQ*, Rif^r^ Tet^r^	This study
BER‐165	638R *Δpir2::cfxA*, Rif^r^ Cfx^r^	This study
BER‐166	638R *Δpir1::tetQ Δpir2::cfxA* Rif^r^ Tet^r^ Cfx^r^	This study
BER‐173	BER‐164 carrying pER‐287, *pir1* ^*+*^, Rif^r^ Tet^r^ Erm^r^	This study
BER‐174	BER‐165 carrying pER‐288, *pir2* ^*+*^, Rif^r^ Cfx^r^ Erm^r^	This study
BER‐175	BER‐166 carrying pER‐287, *pir1* ^*+*^, Rif^r^ Tet^r^ Cfx^r^ Erm^r^	This study
BER‐176	BER‐166 carrying pER‐288, *pir2* ^*+*^, Rif^r^ Tet^r^ Cfx^r^ Erm^r^	This study
BER‐183	638R *Δtdk*, Rif^r^ FUdR^r^	Parker et al. (2022)
BER‐231	638R *ΔPFOR::tetQ*, Rif^r^ Tet^r^	This study
BER‐282	638R *pyc*::pFD516, Rif^r^ Erm^r^	This study
BER‐285	638R made metronidazole resistant at 2 μg/ml	This study
BER‐286	638R made metronidazole resistant at 4 μg/ml	This study
BER‐287	638R made metronidazole resistant at 8 μg/ml	This study
BER‐289	638R made metronidazole resistant at 16 μg/ml	This study
BER‐293	638R made metronidazole resistant at 32 μg/ml	This study
BER‐294	638R *Δkor1AB*, Rif^r^	This study
BER‐295	638R *Δkor2AEBG*, Rif^r^	This study
BER‐296	638R *ΔPFOR::tetQ Δkor1AB,* Rif^r^	This study
BER‐298	638R *ΔpoxB*, Rif^r^	This study
BER‐299	638R *ΔPFOR::tetQ ΔpoxB*, Rif^r^	This study
BER‐300	638R *Δkor1AB ΔpoxB*, Rif^r^	This study
BER‐301	638R *ΔPFOR::tetQ Δkor1AB ΔpoxB*, Rif^r^	This study
BER‐302	638R Δ*PFOR::tetQ Δkor2AEBG*, Rif^r^	This study
BER‐303	638R *ΔPFOR::tetQ Δkor1AB Δkor2AEBG*, Rif^r^	This study
BER‐308	638R *ΔcitS Δicd ΔacnA*, Rif^r^	This study
BER‐309	638R *Δkor2AEBG ΔcitS Δicd ΔacnA*, Rif^r^	This study
BER‐310	638R *ΔPFOR::tetQ Δkor2AEBG ΔcitS Δicd ΔacnA*, Rif^r^	This study
BER‐311	638R *ΔPFOR::tetQ Δkor1AB Δkor2AEBG ΔcitS Δicd ΔacnA*, Rif^r^	This study
BER‐323	BER‐183 *ΔfnrC Δtdk*, Rif^r^ FUdR^r^	This study
IB260	638R *ΔkatB::tetQ*, Rif^r^ Tet^r^	Rocha et al. (1996)
IB263	638R hydrogen peroxide resistant, *hpr*, Rif^r^	Rocha & Smith (1998)
IB274	638R *ahpCF::*pFD516, Rif^r^ Erm^r^	Rocha & Smith (1999)
IB276	638R *ahpF::*pFD516, Rif^r^ Erm^r^	Rocha & Smith (1999)
IB336	638R *Δdps::tetQ*, Rif^r^ Tet^r^	Rocha & Smith (2004)
IB370	638R *ΔtrxB*::*cfxA*, Rif^r^ Cfx^r^	Rocha et al. (2007)
IB430	638R *ΔahpC::tetQ*, Rif^r^ Tet^r^	Lab strain
IB483	ADB77 reverted to *thyA* ^+^, *ΔtrxC ΔtrxD*::*cfxA ΔtrxE ΔtrxF ΔtrxG* Rif^r^ Cfx^r^	Reott et al. (2009)
IB500	IB483 *ΔoxyR::tetQ* Rif^r^ Cfx^r^ Tet^r^	Reott et al. (2009)
IB542	IB336 *Δbfr::cfxA*, Rif^r^ Tet^r^ Cfx^r^	Betteken et al. (2015)
IB567	IB542 pFD288::*bfr* ^*+*^, Rif^r^ Tet^r^ Cfx^r^ Erm^r^ *bfr* ^*+*^	Betteken et al. (2015)
IB573	IB542 pFD288::*dps* ^*+*^, Rif^r^ Tet^r^ Cfx^r^ Erm^r^	Betteken et al. (2015)
ADB77	638R isogenic *ΔthyA* Rif^r^ Tp^r^	Baughn & Malamy (2002)
ADB247	ADB77 *ΔfrdC247* reverted to *thyA* ^+^, Rif^r^	Baughn & Malamy (2002)
ADB260	ADB77 *ΔfrdB260 ΔthyA* Rif^r^ Tp^r^	Baughn & Malamy (2002)
BER‐274	ADB77 *ΔrelA ΔspoT* reverted to *thyA* ^+^, Rif^r^	Lab strain
BER‐278	ADB247 carrying pER‐364, *frdC* ^*+*^, Rif^r^ Erm^r^	This study
BER‐283	ADB260 carrying pER‐365, *frdB* ^*+*^, *ΔthyA* Rif^r^ Tp^r^ Erm^r^	This study
*B. fragilis* BF8	chromosomal *nimB* Ni^r^	Haggoud et al. (1994)
*C. difficile*	ATCC 43255	ATCC
*P. gingivalis*	ATCC 33277	ATCC
*Pr. melaninogenica*	ATCC 25845	ATCC
*E. coli*		
DH10B	cloning host strain	Invitrogen
HB101::RK231	HB101 containing RK231, (Km^r^) (Tet^r^) (Str^r^)	Guiney et al. (1984)
S17‐1 λpir	Strain with the RK2 *tra* genes for conjugative transfer integrated in the chromosome *(RP4‐2‐Tc::Mu‐Km::Tn7, pro, res* ^−^ *mod* ^*+*^, Tp^r^ Sm^r^) λ*pir* lysogen.	Simon et al. (1983)
XL1‐Blue MRF'	Δ(*mcrA*)*183* Δ(*mcrCB‐hsdSMR‐mrr*)*173 endA1 supE44 thi‐1 recA1 gyrA96 relA1 lac* [F' *proAB lacI* ^q^ *Z*Δ*M15* Tn*5* (Kan^r^ *)*].	Stratagene
BacterioMatch II Reporter stain	Δ(*mcrA*)183 Δ(*mcrCB‐hsdSMR‐mrr*)173 *endA1 hisB*	Stratagene
	*supE44 thi‐1 recA1 gyrA96 relA1 lac* [F' *lacI* ^*q*^ *His3 aadA Kan* ^*r*^]	
Plasmids		
pTRG	BacterioMatch II two‐hybrid system target plasmid,	Stratagene
pBT	BacterioMatch II two‐hybrid system bait plasmid,	Stratagene
pNBU2‐*bla‐ermGb*	NBU2 integrase (*intN2*) based genomic insertion vector derived from pKNOCK‐*bla*‐*ermGb* inserts into NBU2 *att1* or *att2* sites of tRNA^ser^. (Amp^r^) Erm^r^	Koropatkin et al. (2008)
pNBU2*‐bla‐tetQb*	NBU2 integrase (*intN2*) based genomic insertion vector derived from pKNOCK‐*bla*‐*tetQb* inserts into NBU2 *att1* or *att2* sites of tRNA^ser^. (Amp^r^) Tet^r^	Martens et al. (2008)
pExchange‐*tdk*	Derivative of pKNOCK‐*bla*‐*ermGb* carrying cloned *tdk* gene for counter‐selection. (Amp^r^) Erm^r^	Koropatkin et al. (2008)
pFD288	*Bacteroides‐E. coli* shuttle vector, *oriT*, pUC19::pBI143 chimera, (Sp^r^) Erm^r^	Smith et al. (1995)
pFD340	*Bacteroides*‐*E. coli* expression shuttle vector. (Amp^r^) Erm^r^	Smith et al. (1992)
pFD516	suicide vector, derived from deletion of pBI143 in	Smith et al. (1995)
pLGB36	Suicide vector for allelic replacement in *Bacteroides* species including *B. fragilis* strain 638R, Erm^r^ selection and aTC‐inducible ss‐Bfe3 counterselection.	Ito et al. (2020)
pER‐63	A 2.1 kb BamHI/EcoRI DNA fragment containing *cfxA* cassette gene was cloned into the BamHI/EcoRI sites of pFD516. (Sp^r^), Erm^r^.	This study
pER‐65	A 2.4 kb BamHI/SacI DNA fragment containing *tetQ* gene cassette was cloned into the BamHI/SacI sites of pFD516. (Sp^r^), Tet^r^.	This study
pER‐283	A 1.9 bp N‐terminal BglII/BamHI and a 1.935 bp SstI C‐terminal DNA fragments flanking BF638R_3039 gene was cloned, respectively, into BamHI and SacI sites of the suicide‐vector pER‐65 to construct *Δpir1::tetQ* deletion mutant. (Sp^r^).	This study
pER‐284	A 1.240 bp N‐terminal BglII/BamHI and a 1.830 bp EcoRI C‐terminal DNA fragments flanking BF638R_1469 gene was cloned, respectively, into the BamHI and EcoRI sites of the suicide‐vector pER‐63 to construct *Δpir2::cfxA* deletion mutant (Sp^r^)	This study
pER‐287	An 0.808 kb DNA fragment containing promoterless *pir1* gene was cloned into the BamHI/SacI sites of the expression vector pFD340. (Amp^r^) Erm^r^	This study
pER‐288	A 0.746 kb kb DNA fragment containing promoterless *pir2* gene was cloned into the BamHI/SacI sites of the expression vector pFD340. (Amp^r^) Erm^r^	This study
pER‐364	A 0.96 kb DNA fragment containing promoterless *frdC* gene was cloned into the BamHI/KpnI sites of the expression vector pFD340. (Amp^r^) Erm^r^	This study
pER‐365	A 1.040 kb DNA fragment containing promoterless *frdB* gene was cloned into the BamHI/SacI sites of the expression vector pFD340. (Amp^r^) Erm^r^	This study
pER‐372	A 2,461 DNA fragment containing 2,435 bp in‐frame null deletion of *kor1AB* genes (BF638R_4321‐4322) was cloned into the BamHI/SalI sites of pLGB36 vector. (Amp^r^) Erm^r^	This study
pER‐373	A 2,040 DNA fragment containing 2,354 bp in‐frame null deletion of *kor2ABG* genes (BF638R_1655‐1658) was cloned into the BamHI/XmaI sites of pLGB36 vector. (Amp^r^) Erm^r^	This study
pER‐375	A 3,723 DNA fragment containing 1,350 bp in‐frame null deletion of *poxB* gene (BF638R_3245) was cloned into the BamHI/SalI sites of pLGB36 vector. (Amp^r^) Erm^r^	This study
pFD1198	A 1 kb DNA fragment upstream and 1 kb DNA fragment downstream of *fnrA* gene (BF638R_0432) were clone into the BamHI/SacI and SacI/EcoRI sites of pFD516. A 2.3 TetQ cassette was cloned into the SacI site to replace an internal 700 bp deleted DNA fragment (Spr) Erm^r^.	This study
pFD1245	An 802 bp insertional inactivation fragment of the *pyc* gene (BFR638R_1927) cloned into the BamHI/EcoRI sites of pFD516. (Sp^r^) Erm^r^	This study
pFD1277	A 2,243 bp DNA fragment containing 566 bp in‐frame null deletion of *fnrC* gene (BF638R_1017) was cloned into the PstI/XbaI sites of pExchange‐*tdk* vector. (Amp^r^) Erm^r^	This study
pFD1278	A 2.002 kb N‐terminal SphI/BamHI and a 1.819 SacI flanking BF638R_3194 gene was cloned, DNA fragments into the SphI/BamHI and SacI sites of the suicide‐vector pER‐65 to construct *ΔPFOR::tetQ* deletion mutant. (Sp^r^).	This study

*Note*: Parenthesis indicates antibiotic resistance expression in *E. coli.*

Abbreviations: Amp^r^, ampicillin resistance; ATCC, American type culture collection; Cfx^r^, cefoxitin resistance; Erm^r^, erythromycin resistance; FUdR^r^, 5‐fluor‐2′‐deoxyuridine resistance; Kan^r^, kanamycin resistance; Ni^r^, 5‐nitroimidazole resistance; Rif^r^, rifampicin resistance; Sp^r^, spectinomycin resistance; Str^r^, streptomycin resistance; Tet^r^, tetracycline resistance; Tp^r^, trimethoprim resistance

#### 2.1.1 Bacterial two‐hybrid system assay

##### Screening partial chromosomal library for protein‐protein interactions with Pir1 or Pir2

The chromosomal DNA from *B. fragilis* 638 R was partially restricted with Sau3AI to obtain fragments with average size within the range of 0.5–5 kb as previously described (Rocha & Smith, 1995). A genomic library was constructed by ligating the partial Sau3AI fragments into the unique BamHI site downstream of the RNAP α‐subunit in the bacterial two‐hybrid system (THS) target plasmid pTRG (BacterioMatch II; Stratagene) and amplified in *E. coli* XL1‐Blue MRF' strain (Stratagene). The pirin 1, Pir1, (GenBank locus‐tag BF638R_3039) or pirin 2, Pir2, (BF638R_1469) ORFs were amplified with primers described in Table S1 and cloned in‐frame with the Lambda repressor gene (*λcl*) under the control of the *lac‐UV5* promoter in the bait plasmid pBT according to manufacturer's instructions. The new constructs, pBT/Pir1 or pBT/Pir2 were cotransformed with the pTRG/genomic library into the *E*. *coli* THS reporter strain and plated on a selective screening medium containing 5 mM 3‐amino‐1,3,4‐triazole (3‐AT). The isolated two‐hybrid system positive transformants were plated on a dual‐selective medium containing 5 mM 3‐AT and 12.5 μg/ml streptomycin for validation as recommended by the manufacturer. The nucleotide sequence of the DNA fragment inserted into the TRG plasmid from each of the selected THS transformants was obtained using the pTRG forward primer and the deduced amino acid sequences were obtained.

##### Protein‐protein interaction of Pir1 or Pir2 with PFOR and Zn‐ADH

The entire ORF of PFOR and Zn‐ADH were amplified by PCR and cloned in‐frame with the RNA α‐subunit of pTRG plasmid to construct pTRG/PFOR and pTRG/ADH, respectively. The pTRG/PFOR and pBT/Pir1, the pTRG/PFOR and pBT/Pir2, the pTRG/ADH and pBT/Pir1, or the pTRG/ADH and pBT/Pir2 plasmids were cotransformed into the *E*. *coli* THS reporter strain, respectively, and selected on nonselective medium, selective medium, and dual‐selective medium exactly as previously described (Robertson et al., 2006).

##### Prediction of protein‐protein interactions using computational modeling

Structure‐based computational modeling of protein‐protein interactions was used to assess the potential contributions of side‐chain atoms in the interactions of Pir1 and Pir2 with PFOR, Zn‐ADH, and with 140 enzyme subunits of the central metabolism and energy conservation of the TCA cycle, pyruvate metabolism, branched‐chain amino acid aminotransferase (BCAAT), ThPP‐binding enzymes, carboxylases/decarboxylases, oxidoreductases and dehydrogenases. The 3‐dimensional structure of heteromeric protein‐protein interactions was predicted using Alphafold2‐Multimer https://colab.research.google.com/github/sokrypton/ColabFold/blob/main/AlphaFold2.ipynb (Evans et al., 2021; Jumper et al., 2021; Mirdita et al., 2022). Amino acid sequences of Pir1 and Pir2 along with other target enzymes were used as primary inputs for Alphafold2‐Multimer. For models involving the interaction of Pir1 and Pir2 with PFOR, final models were also subjected to 2000 steps of energy minimization using an AMBER force field and in these specific cases, the final energy relaxed models were used for interface analysis. Resulting models were analyzed using the Protein Interaction Z‐Score Assessment (PIZSA) webserver http://cospi.iiserpune.ac.in/pizsa (Roy et al., 2019) using a distance threshold default 4 Å to define interface residues contacts for the potential contributions of side chain atoms in the protein‐protein interactions. A Z‐score threshold ≥1.500 defines a stable association. The interface area (Å^2^) and the buried surface area (Å^2^) of the interacting residues were calculated using the Proteins, Interfaces, Structures and Assemblies (PDBePISA) webserver https://www.ebi.ac.uk/msd-srv/prot_int/pistart.html (Krissinel & Henrick, 2007).

##### HPLC analysis of short‐chain fatty acids

Bacteria were grown to mid‐logarithmic phase (OD_550nm_ to 0.3–0.4) or for 24 h anaerobically in peptone yeast extract basal medium containing 0.5% d‐glucose (PYG) prepared as described previously (Holdeman et al., 1977). Anaerobic mid‐log cultures were split, and one half were exposed to atmospheric air for 1 h or 24 h in an aerobic shaker incubator at 37℃. For iron restriction, 2,2'‐dipyridyl (50 μM) was added to the medium. After pelleting cultures, the clear supernatants were passed through 0.22 μm filters. Samples were analyzed for SCFAs using a Bio‐Rad HPLC organic acid system with AMINEX 87H, 300 × 7 mm column with 5 mM sulfuric acid eluant at 0.6 mL/min, 65℃, with refractive index detector. Uninoculated media were used as blank and the media background was subtracted except for glucose peak. The HPLC analysis was performed at the USDA Agricultural Research Service.

##### RNA extraction and RT‐PCR analysis

Bacteria were grown in a chemically defined medium supplemented with 100 μM ferrous sulfate or 50 μM 2,2'‐dipyridyl to mid‐logarithmic phase anaerobically and exposed to atmospheric air for 1 h. Total RNA was extracted from bacterial pellet using the hot‐phenol method as described previously (Rocha & Smith, 1997), and cleaned using RNeasy Mini kit (Qiagen) according to manufacturer instructions. RNA was DNAse treated using the Ambion DNA‐free protocol (Ambion, Inc.). First‐strand cDNA synthesis was carried out from 1 μL total RNA at 1 μg/μL with random hexamer primers and Superscript III RT kit (Invitrogen Inc.) according to the manufacturer's instructions. Real‐time PCR quantification of each pirin mRNA was performed with 1 μL cDNA sample diluted 1:10 and forward and reverse primers described in Table S1. Real‐time PCR efficiencies were performed for each primer set. The 16S rRNA was used as a reference to normalize gene expression to a housekeeping gene. Relative expression values were calculated using the Pfaffl method (Pfaffl, 2001). Fold induction relative to the wild type in anaerobic conditions was determined for each gene using 16S RNA as the reference gene and all results were the average of at least two independent experiments with freshly isolated RNA.

### 2.2 Antibiotic susceptibility assays

The agar dilution method for minimal inhibitory concentration (MIC) determination and the disc inhibition assays were performed with BHI agar (20 mL/plate) containing heme (5 μg/mL). Overnight bacterial cultures in BHIS were diluted in PBS to a density of approximately 0.5 MacFarland scale. Five μL of suspension was applied on plates containing different concentrations of MTZ or AMIX. Disc inhibition was performed by spreading bacterial suspension with a swab. Five μL of 1 mg/mL MTZ solution or 10 μl of 1 mg/mL sterile AMIX in aqueous solution was applied on top of a sterile 6 mm disc paper. Plates were inoculated in duplicate. One set was incubated for 48 h anaerobically at 37℃, and the other set was incubated aerobically at 37℃ for 24 h before anaerobic incubation for 48 h. Thymine (50 μg/mL) and sodium succinate (20 mM) were added to the media when required for growth of the *frdB* and *frdC* mutants and their parent strain ADB77, a BF638R *ΔthyA* isogenic strain (Table 1). The zone of inhibition around the disc was measured in mm. Electron transport system inhibitors (ETS) or redox cycling agents were added to the medium when required. The ETS inhibitors were added to BHI plates at concentrations 2 to 4‐fold lower than the amount needed to cause growth inhibition of *B. fragilis* 638 R as indicated in the text. The growth inhibition concentrations of ETS are as follows: Closantel, 12.5 μM; acriflavine, 100 μM; 2‐heptyl‐hydroxyquinoline‐N‐oxide (HQNO), 120 μM; antimycin A, >200 μM; rotenone, >200 μM; and 2‐mercaptopyridine‐N‐oxide (2‐MPNO), 50 μM.

#### 2.2.1 Construction of mutant strains

Deletion mutants with an antibiotic cassette replacing the gene internal DNA deleted fragment were constructed using pFD516 as a suicide vector to mobilize mutated DNA fragments from *E. coli* DH10B into *B. fragilis* 638 R for recombinant genetic exchange as described previously (Robertson et al., 2006; Rocha & Smith, 2004; Rocha et al., 2007). The forward and reverse primers used to PCR amplify DNA fragments to construct a deleted DNA fragment are shown in Table S1. The construction of null deletion mutants in *B. fragilis* 638 R was carried out using a pLGB36 suicide vector for allelic replacement as described previously (Ito et al., 2020). Briefly, the pLGB36 constructs in *E. coli* S17‐1 λpir strain were mobilized to *B. fragilis* 638R by biparental mating and transconjugants were selected on BHIS plates containing rifampicin (20 μg/ml), gentamycin (100 μg/mL) and erythromycin (10 μg/mL). A colony of erythromycin‐resistant first crossed‐over recombinant strain was grown on 5 mL BHIS containing rifampicin (20 μg/mL), gentamycin (100 μg/mL), and without erythromycin, until OD_550 nm_ of 0.3–0.4. Then, 100 ng/mL aTC was added and the culture was incubated for approximately 2–3 h to induce the ss‐Bfe3 killer protein for counterselection. Ten μL aliquots were removed and spread on four plates of BHIS containing rifampicin (20 μg/mL), gentamycin (100 μg/mL), and aTC (100 ng/mL). After incubation for 3–4 days, colony PCR was performed using forward and reverse primer sets described in Table S1 to identify transconjugants with chromosomal deletion fragments compared to the parent strain. Erythromycin susceptibility was performed to confirm the loss of the suicide vector.

#### 2.2.2 Intra‐abdominal infection

All procedures involving animals followed the guidelines given by the National Research Council's *Guide for the Care and Use of Laboratory Animals* (National Research Council, 2011) and approved by the Institutional Animal Care and Use Committee of East Carolina University. The rat tissue cage model of intra‐peritoneal infection was performed exactly as described previously (Lobo et al., 2013) to test the in vivo efficacy of AMIX against the *B. fragilis* 638 R strain. Four groups of three Sprague‐Dawley rats were infected with 4 ml of approximately 1 × 10^5^ CFU suspension in PBS into the tissue cage. One group was administered AMIX 20 mg/kg once daily intraperitoneally (IP) from Day 1 through Day 7 postinfection. The second group received AMIX 0.5 mg injected intra‐cage to obtain approximately 20 μg/ml final concentration. This expected intra‐cage concentration corresponds to the AMIX concentration reached in rat serum receiving AMIX 20 mg/kg/day via oral (Hoffman et al., 2014). Intra‐cage injection of AMIX was administered once daily at Day 1 through Day 7 postinfection. The other two groups received saline administered IP or intra‐cage as control. Intra‐cage tissue fluid was aspirated on Day 1, Day 2, Day 4, and Day 8 postinfection, serially diluted, and plated on BHIS. After 3 to 4 days of incubation in an anaerobic chamber at 37℃, colonies were counted and normalized to CFU/mL of tissue cage fluid. The limit of detection was 1 × 10^1^ CFU/mL.

### 2.3 Statistical analysis

Built‐in statistical analysis in the GraphPad Prism software version 10.2.3 was performed for disc inhibition data using one‐way ANOVA followed by Dunnett and Bonferroni tests for multiple comparisons of the means of each column with the mean of a control column. Only P values with significance in both tests were considered statistically significant. GraphPad Prism reports the summary of *p* value as follows: *p* ≥ 0.05, not significant (ns); *p* = 0.01–0.05, significant (*); *p* = 0.001–0.01, very significant (**); *p* = 0.0001–0.01 (***) and *p* < 0.0001 (****) as extremely significant.

## 3 RESULTS

### 3.1 The expression of *pir1* and *pir2* genes in response to oxygen and iron limitation

**Figure 1:**
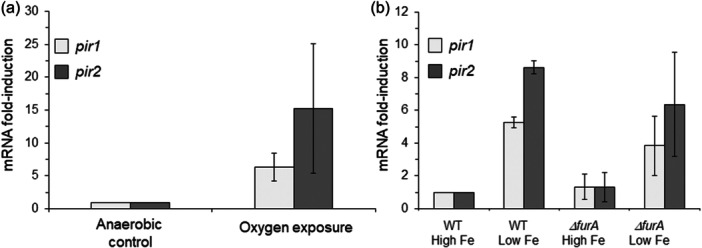
Fold‐induction of *pir1* and *pir2* mRNA following (a) oxygen exposure or (b) growth in iron‐limiting conditions. In panel a, the BF638R parent strain was grown to the mid‐logarithmic phase in BHIS and exposed to atmospheric air for 1 h. In panel b, the BF638R parent strain and its isogenic *ΔfurA* strain were grown to mid‐logarithmic phase in defined medium with protoporphyrin IX supplemented with 100 μM FeSO_4_ (High Fe) or with 50 μM 2,2’‐dipyridyl (Low Fe). For each condition, RNA was isolated and real‐time RT‐PCR was performed in triplicate. The 16S rRNA gene was used as an internal standard, and the results are expressed as fold induction relative to levels in the control condition. The values are means of fold induction compared to control from two independent experiments. The error bars indicate standard deviations.

*B. fragilis* 638R contains two pirin‐like proteins, Pir1 (BF638R_3039) and Pir2 (BF638R_1469). A genome transcription microarray of iron and heme‐regulated genes showed that both *pir1* and *pir 2* genes are upregulated by iron limitation (NCBI GEO DataSets GSE241210 and GSE241676, unpublished) and following oxygen exposure (Sund et al., 2008). Real‐time RT‐PCR using total RNA confirmed that *pir1* and *pir2* mRNAs were induced over sixfold and 15‐fold in the presence of oxygen, respectively (Figure 1a), and over fivefold and eightfold under limiting iron conditions compared to the parent strain, respectively (Figure 1b). Furthermore, deletion of the ferric uptake regulator, *ΔfurA*, did not significantly alter the *pir1* or *pir2* iron‐regulated expression under high‐iron conditions compared to the parent strain. This indicates that the iron regulation of both *pir1* and *pir2* mRNA expression is FurA‐independent.

A phylogenetic unrooted tree constructed from multiple amino acid alignments showed that the *Bacteroides* species Pir1 and Pir2 orthologs are grouped into two distinct clusters in a branch divergent from other members of the Bacteroidetes phylum (Supplemental File S1). The multiple amino acid sequence alignment revealed that the N‐terminal domain residues His56, His58, His101, and Glu103 ligands of the iron center of human pirin (Liu et al., 2013; Pang et al., 2004) are conserved in both Pir1 and Pir2 His58, His60, His102, and Glu104, respectively (Supplemental File S2).

### 3.2 The two‐hybrid system identifies Pir1 and Pir2 protein‐protein interactions with PFOR and a Zn‐ADH

**Figure 2:**
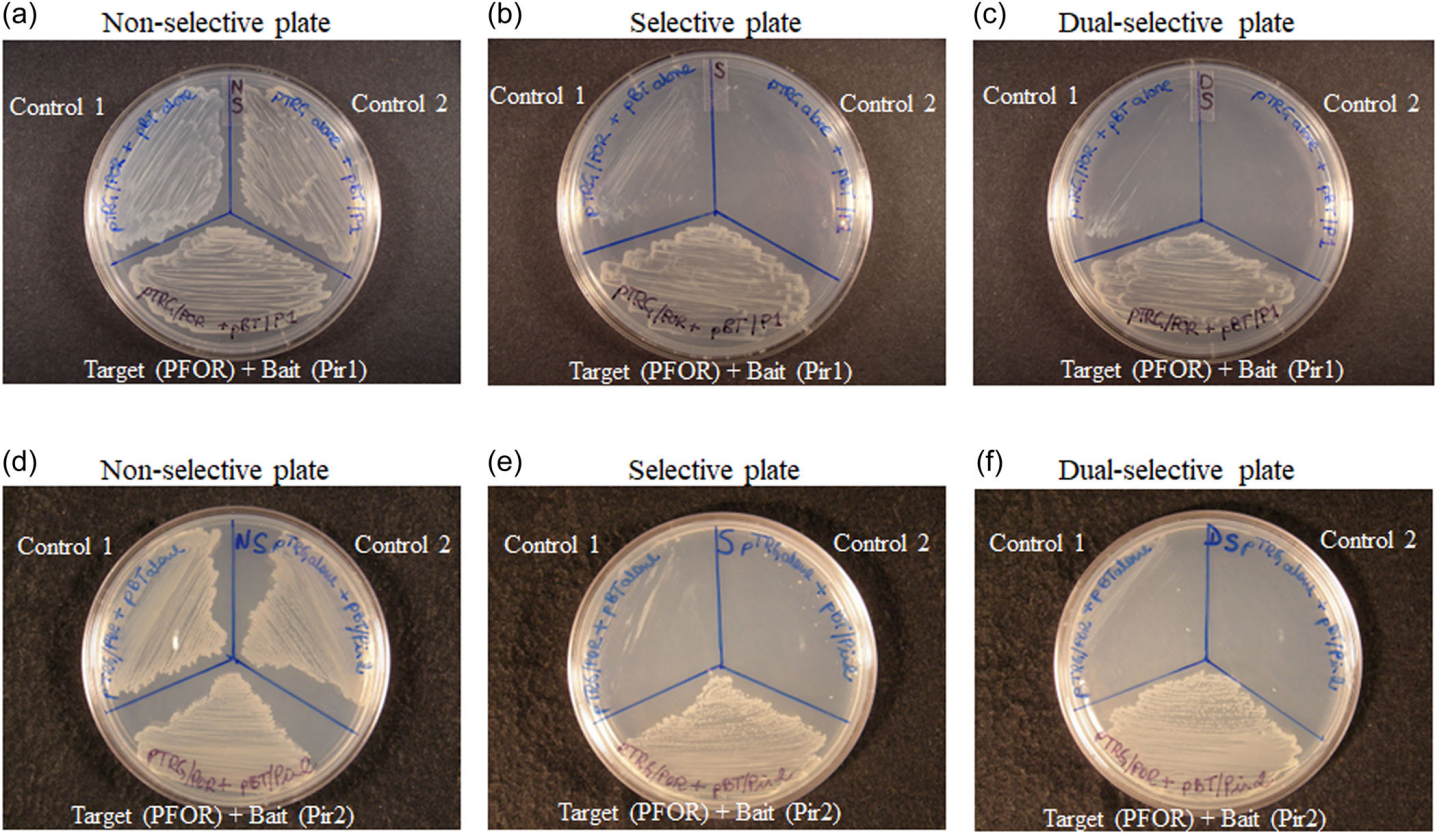
Bacterial two‐hybrid system (THS) assay showing protein‐protein interaction of PFOR with Pir1 (panels a, b, and c) and PFOR with Pir2 (panels d, e, and f). *E*. *coli* THS reporter strain cotransformed with pBT/Pir1 (bait) and pTRG/PFOR (prey) constructs are shown in panels a, b and c. *E*. *coli* THS reporter strain cotransformed with pBT/Pir2 (bait) and pTRG/PFOR (prey) constructs are shown in panels d, e and f. Bacteria were grown on a control nonselective plate (a and d), selective plate (b and e), and dual selective plate (c and f). Self‐activation controls are *E*. *coli* THS reporter strain carrying the following constructs: Control 1, empty “bait” (pBT alone) cotransformed with loaded “prey” (pTRG/PFOR) in panels a–f; Control 2, loaded “bait” (pBT/Pir1) cotransformed with empty “prey” (pTRG alone) in panels a, b, and c, or loaded “bait” (pBT/Pir2) cotransformed with empty “prey” (pTRG alone) in panels d, e, and f. In panels 2e and 2f, 7 mM 3‐amino‐1,3,4‐triazole (3‐AT) was used instead of 5 mM 3‐AT used in panels 2b and 2c. See Materials and Methods for details on the bacterial two‐hybrid system assay.

An *E. coli* THS assay (BacterioMatch II; Stratagene) was used to screen a *B. fragilis* 638R partial Sau3AI genomic library cloned in the target plasmid (pTRG) for expression of peptides forming protein‐protein interactions with either Pir1 or Pir2 protein in the bait plasmid (pBT). Over 50,000 co‐transformed colonies were plated on selective THS media, and 12 colonies carrying pTRG/cloned insert interacting with pBT/Pir1 and 11 colonies carrying pTRG/insert interacting with pBT/Pir2 grew on selective media and confirmed by growth on dual‐selective media. This indicated that peptides expressed from the pTRG/insert were positively interacting with Pir1 or Pir2. The deduced amino acid sequence in‐frame with the C‐terminal region of the RNAP α‐subunit of each pTRG/insert interacting with pBT/Pir1 or pBT/Pir2 is shown in Supplemental File S3. Three deduced peptide sequences showed homology to PFOR (BF638R_3194) interacting with Pir1, and six clones showed homology to zinc‐binding alcohol dehydrogenase, Zn‐ADH (BF638R_1292), one interacting with Pir1 and the other five with Pir2. To confirm these findings, the entire PFOR ORF (BF638R_3194) or Zn‐ADH ORF was cloned in‐frame with the RNA‐α subunit into the pTRG vector. The new constructs, pTRG/PFOR and pTRG/ADH were co‐transformed with the pBT/Pir1 or pBT/Pir2 into the two‐hybrid system reporter strain as described above. The THS assays showing protein‐protein interaction of PFOR with Pir1 are shown in Figure 2b,c, and Zn‐ADH with Pir1 or Pir2 is shown in Supplemental File S4. Non‐negligible/slight growth of control 1 was observed in Figure 2b,c.

### 3.3 AlphaFold2‐multimer‐based modeling of protein‐protein interactions between Pir1 or Pir2 with metabolic enzymes

**Figure 3:**
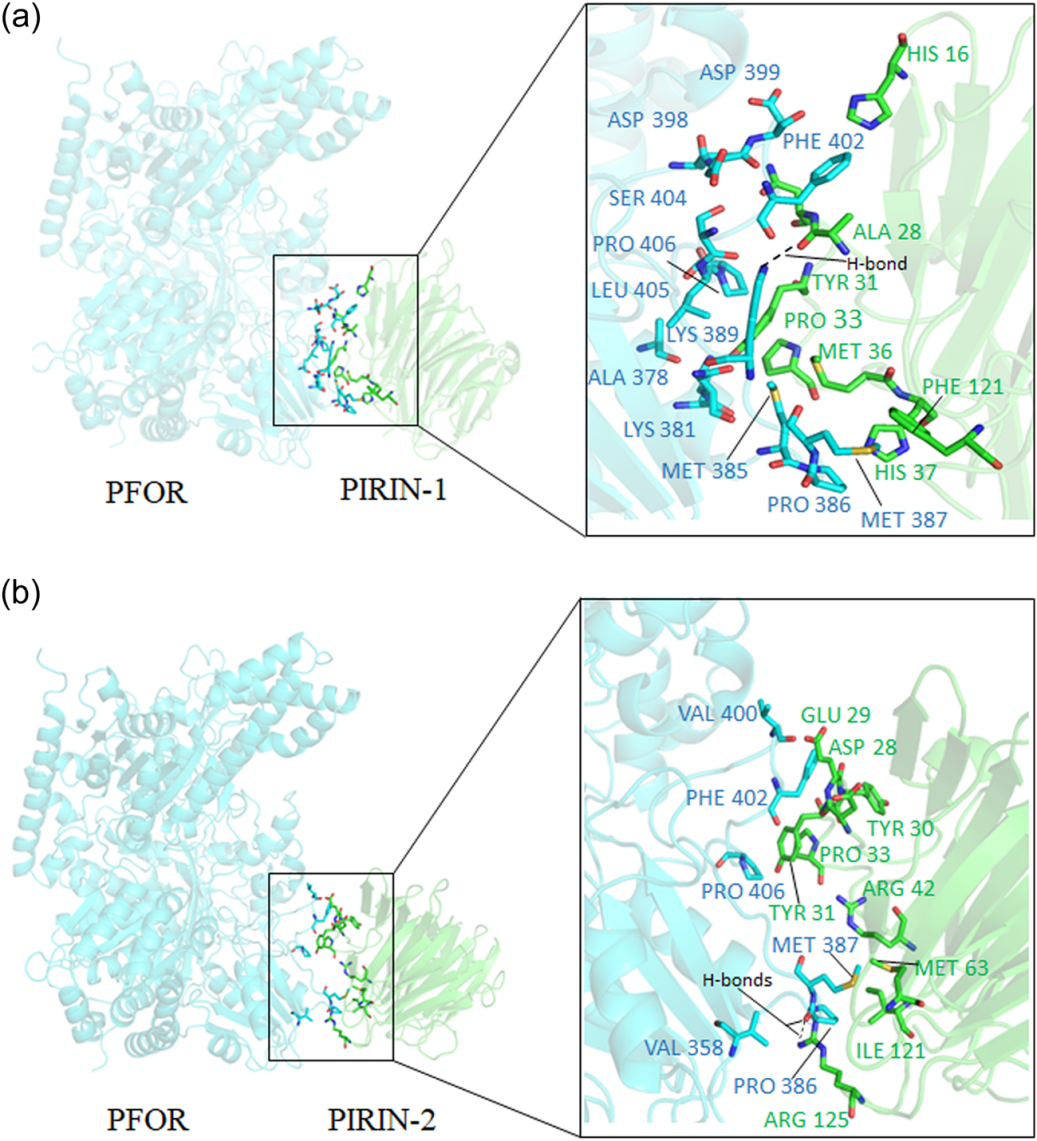
Ribbon cartoon diagram of AlphaFold2 Multimer 3D‐structure modeling of the (a) Pirin1:PFOR and (b) Pirin2:PFOR complexes. Pirin proteins are shown in green and PFOR in cyan. Residue interaction pairs are drawn with stick models (Inset). Amino acids making contact in the protein‐protein interface are depicted with font color corresponding to each chain. A list of the amino acid interaction pairs and their relative buried surface area is shown in Supplemental file S5A. See Material and Methods section for additional details on the computational modeling methodology. Images were generated using PyMol molecular graphics system version 2.5.8 (Schrödinger, LLC).

To model protein‐protein interactions of Pir 1 and Pir2 with PFOR, we used AlphaFold2‐Multimer to predict 3D structures of Pir1:PFOR and Pir2:PFOR complexes. In agreement with the THS assay, the final relaxed AlphaFold2 models showed stable interactions of PFOR with Pir1 as judged by PISZA analysis (Z‐score of 1.710 [stable association >1.5]; Figure 3a, Supplemental File S5A). Although we did not obtain any genomic library clone indicating interactions of PFOR with Pir2, AlphaFold2 also predicted stable interactions of PFOR with Pir2 with a Z‐score of 1.949 (stable association >1.5; Figure 3b, Supplemental File S5A). To test this prediction, a THS assay was carried out using pTRG/PFOR and pBT/Pir2 constructs to co‐transform the *E. coli* THS reporter strain. Indeed, this experiment strongly supports that a direct interaction is formed between Pir2 and PFOR (Figure 2e,f) though a negligible/slight growth of control 1 was observed in both Figure 2e,f.

These findings prompted us to perform additional 3D‐structural modeling using Pir1 or Pir2 to understand if stable association with other metabolic enzymes of the central metabolism and energy conservation processes are also predicted. For this purpose, AlphaFold2‐Multimer predictions using unrelaxed mode were used to broadly screen putative protein‐protein interactions of Pir1 and Pir2 with enzymes involved in the biochemical pathways depicted in Supplemental File S5, including Zn‐ADH. The resulting 3D structural models were then used to calculate the PISZA‐interface Z‐score and the buried surface area of the interacting amino acids with Pir1 or Pir2 (Supplemental File S5). In agreement with the THS screen (Supplemental File S4), stable interactions of Zn‐ADH with Pir1 or Pir2 were predicted using this approach (Supplemental File S5A). Interestingly, 15 of the additional 140 tested enzymes were predicted to form stable protein‐protein interactions with Pir1, 17 with Pir2, and 17 with both Pir1 and Pir2 (Supplemental File S5A), while 91 of the enzymes screened did not form stable protein‐protein interactions with neither Pir1 nor Pir2 (Supplemental File S5B). Of the 49 proteins interacting with Pir1, Pir2 or Pir1 and Pir2, 32 enzymes belong to the oxidoreductase functional class, 9 are transferases, 2 are lyases, 2 are ligases, 2 proteins contain tetratricopeptide repeat (pfam13424 and pfam00515), 1 isomerase, and 1 conserved hypothetical protein. Among the oxidoreductases, in addition to PFOR, several subunits of the KFORs, Kor1AB and Kor2CDAEBG, were found to form stable protein‐protein interactions with Pir1 and/or Pir2 (Supplemental File S5A). Interestingly, the amino acid residues of Pir1 and Pir2 that are predicted to coordinate stable interactions in 50% of the stable complexes are shared, (i.e., Ala28, Asn29, Tyr31, Pro59 of Pir1 and Asp28, Glu29, Tyr31 of Pir2, Supplemental File S2), indicating that a common binding site may be used.

### 3.4 *pir1* and *pir2* deletion mutants alter the production of some SCFAs

To investigate whether *pir1* and *pir2* gene deletions could affect cellular metabolic activities, fermentation of SCFA products was determined (Supplemental File S6). The results reported are the mean and the data point range from two independent biological replicates determination. Although there were wide‐range variations in the production of some SCFAs by the same strain under the same growth conditions in two independent experiments, we describe here the SCFAs whose amount produced by the *pir1* or *pir2* mutants were clearly altered compared to the parent strain. For example, no lactate or propionate was detected in anaerobic mid‐log phase growth cultures of the parent strain. However, they were produced by the *Δpir1*, *Δpir2*, and *Δpir1 Δpir2* double mutant strains (Supplemental File S6). The lactate produced by the *ΔΔpir1 Δpir2* double mutant strain was approximately fivefold and twofold higher than the amount produced by the *Δpir1* and *Δpir2* mutants, respectively. This suggests that both Pir1 and Pir2 might contribute to the suppression of lactate production in the parent strain during mid‐log phase growth. The amount of propionate produced by the *Δpir1* strain in mid‐log phase growth was reduced approximately threefold in the *Δpir2* and *Δpir1 Δpir2* double mutant strains. In contrast, the amount of isovalerate and phenylacetate produced by the parent strain in mid‐log phase growth was abolished or nearly abolished in the mutant strains. The amount of butyrate produced by the parent strain did not significantly change in the *Δpir1* strain but was reduced approximately fivefold in the *Δpir2* strain and increased approximately threefold in the *Δpir1 Δpir2* double mutant strain compared to the parent strain. The amount of acetate produced in the parent strain was reduced by approximately 50% in both the *Δpir1* and *Δpir1 Δpir2* double mutant while the *Δpir2* deletion did not significantly affect acetate production compared to the parent strain. This suggests that Pir1, but not Pir2, may have a modest effect on acetate production in mid‐log phase growth compared to the parent strain.

In mid‐log phase cultures under low‐iron conditions, the amount of propionate and isovalerate produced by the parent strain were greatly reduced or nearly abolished in all three mutant strains suggesting that Pir1 or Pir2 play a role in the production of propionate and isovalerate. In contrast, the amount of isobutyrate produced by the *Δpir1* strain decreased approximately 15‐fold compared to the *Δpir2* and *Δpir1 Δpir2* double mutant strains while no isobutyrate was observed in the parent strain. This suggests that Pir2 has a stronger effect in the modulatory suppression of isobutyrate production than Pir1 in iron‐limiting conditions. In addition, butyrate was only produced in the *Δpir1 Δpir2* double mutant strain in mid‐log phase growth in iron‐limiting conditions suggesting that Pir1 or Pir2 can repress butyrate as butyrate was only detected in the absence of both *pir1* and *pir2* genes.

Mid‐log phase cultures exposed to oxygen for 1 h did not seem to significantly affect changes in SCFA production by the *pir* mutants except that propionate was not detected in the *Δpir1* strain and a twofold increase was observed in the *Δpir1 Δpir2* double mutant compared to the amount of propionate produced by the parent strain.

There were no distinct changes in SCFAs produced in anaerobic stationary phase cultures after 24 h incubation except that the *Δpir1 Δpir2* double mutant strain produced a minor amount of butyrate compared to its absence in the parent strain and single mutants.

In mid‐log phase cultures exposed to oxygen for 24 h, butyrate was produced in the *Δpir1* and *Δpir1* Δ*pir2* double mutant strains but not in the *Δpir2* mutant or the parent strain cultures. This indicates that Pir1, but not Pir2, modulates the repression of butyrate production in *B. fragilis* in cultures exposed to oxygen for 24 h. Isovalerate was only detected in the *Δpir2* mutant in cultures exposed to oxygen for 24 h but was undetectable in the *Δpir1*, *Δpir1 Δpir2* double mutant and parent strains. Phenylacetate production increased approximately threefold in the *Δpir1*, *Δpir2*, and *Δpir1 Δpir2* double mutant strains compared to the parent strain after oxygen exposure for 24 h.

In cultures exposed to oxygen under low‐iron conditions for 24 h, there was an increase of approximately eightfold and threefold in the production of isobutyrate by the *Δpir2* and *Δpir1 Δpir2* double mutant strains compared to the *Δpir1* and parent strains, respectively. In addition, the amount of butyrate produced by the *Δpir1* or *Δpir2* strains increased approximately two to threefold in the *Δpir1 Δpir2* double mutant strain compared to its undetectable presence in the parent strain. There was a very modest production of formate in iron‐limiting cultures exposed to oxygen for 24 h which was not detected in any of the mutant strains.

In anaerobic stationary phase growth cultures for 48 h, the amount of butyrate produced by the *Δpir2* strain increased approximately fourfold in the *Δpir1 Δpir2* double mutant strain compared to its absence in the parent and *Δpir1* strains. In contrast, the amount of isovalerate produced by the parent and *Δpir1* strains was reduced approximately twofold in the *Δpir2* and *Δpir1 Δpir2* double mutant strains while the amount of phenylacetate produced by the parent and *Δpir1* strains was reduced approximately fourfold in the *Δpir2* and *Δpir1 Δpir2* double mutant strains.

Taken together, these findings indicate that Pir1 and Pir2 affect the upregulation or repression of different metabolic pathways involved mostly in the production of minor SCFA products dependent upon the culture growth conditions. However, the mechanisms that control *B. fragilis* fermentation pathways were not further pursued in this study and remain to be defined.

### 3.5 The effect of *Δpir1* and *Δpir2* mutations and constitutive expression of *pir1* and *pir2* genes in susceptibility to MTZ and AMIX

**Figure 4:**
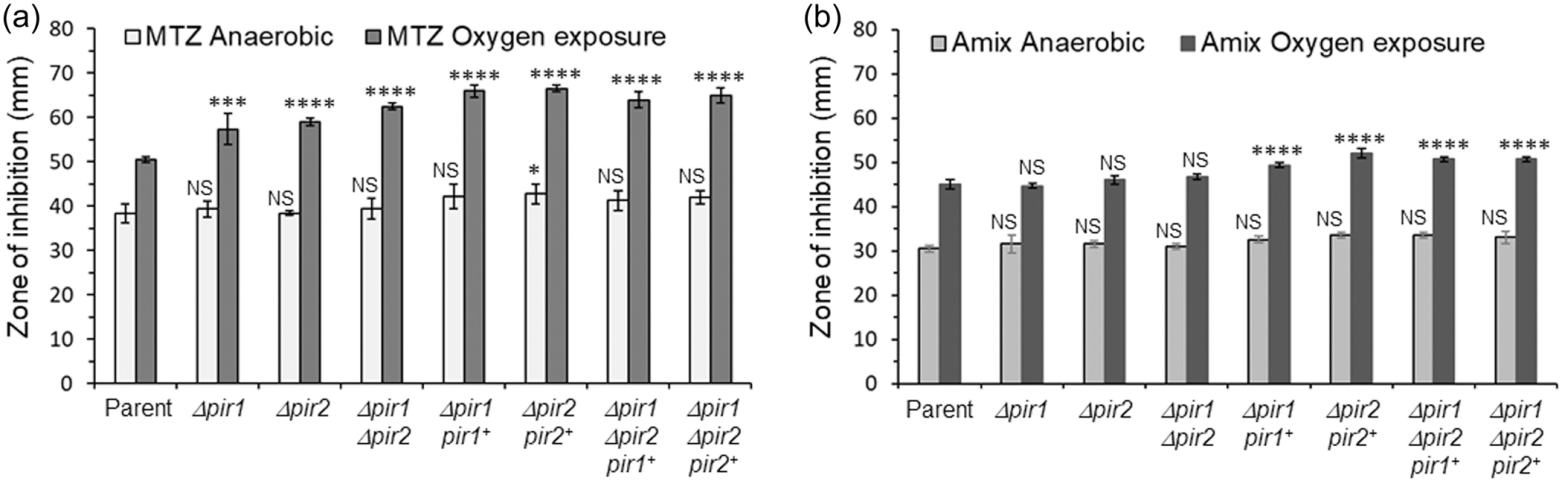
Disc diffusion assay sensitivity for (a) metronidazole (MTZ) and (b) Amixicile (AMIX). *B. fragilis* strains are depicted in each panel. Each bar represents the average zone of inhibition (mm) of at least three independent biological replicates. Vertical error bars denote standard deviation of the means from two independent experiments in triplicate. The significance of the *p* value was calculated by one‐way ANOVA followed by multiple comparisons using Dunnett and Bonferroni tests. Only groups with statistical significance in both tests are reported. *p* < 0.05 (*), *p* < 0.01 (**), *p* < 0.001 (***), and *p* < 0.0001 (****). NS, not significant.

The results above indicate that Pir1 and Pir2 interactions with key enzymes of central metabolism alter metabolic pathways. To investigate this further, we focused our investigation on the effects of *pir1* and *pir2* gene deletions on antimicrobial susceptibility to MTZ and AMIX. In *B. fragilis*, MTZ sensitivity is linked to redox cycling processes that occur during carbon redox steps that flow through its different fermentation pathways, and it is also altered by heme availability (Paunkov et al., 2022a; Paunkov et al., 2022b; Paunkov et al., 2023). To examine if the effect of Pir1 and Pir2 on cellular metabolism described above could alter susceptibility to MTZ and AMIX, MIC determination and disc inhibition assays were performed (Table 2 and Figure 4). The sensitivity and resistance terms used in this study refer to the antimicrobial concentration required to inhibit growth of the parent strain, BF638R, and not to the antimicrobial breakpoint concentration guideline recommended for its clinical use. Disc diffusion assay showed that *Δpir1*, *Δpir2* were statistically more sensitive to MTZ following oxygen exposure compared to the parent strain. The *Δpir1 Δpir2* double‐mutant was more sensitive to MTZ than the *Δpir1* and *Δpir2* single mutant strains (Figure 4a). However, the genetically complemented strains enhanced sensitivity to MTZ compared to mutant strains following oxygen exposure, respectively. There were no significant changes in MTZ susceptibility in anaerobic cultures compared to the parent strain, except for a modest increase in MTZ sensitivity in the *Δpir2 pir2* ^*+*^ strain (Figure 4a). The lack of *pir* genes did not significantly affect sensitivity to AMIX in anaerobic conditions or in cultures exposed to oxygen while the genetically complemented strains were statistically more sensitive to AMIX in oxygen exposed cultures (Figure 4b). It is unclear why genetically complemented strains *Δpir1 pir1* ^*+*^, *Δpir2 pir2* ^*+*^, *Δpir1 Δpir2 pir1* ^*+*^ and *Δpir1 Δpir2 pir2* ^*+*^ enhanced sensitivity to MTZ and AMIX instead of restoring sensitivity to parent strain level following oxygen exposure as determined by disc inhibition assays. Because genetic complementation with regulators or modulators may cause unexpected global phenotype effects, we assume that constitutive expression of pirins may have altered control of unidentified mechanisms that led to enhancement in sensitivity to these antimicrobials.

**Table 2:** Agar dilution determination of minimal inhibitory concentration (MIC μg/mL) of metronidazole (MTZ) and amixicile (AMIX) for *Bacteroides species* and *B. fragilis* 638R mutant strains.

Strains	MTZ anaerobic	MTZ O_2_ exposure	AMIX anaerobic	AMIX O_2_ exposure
BF638R	0.5	0.5	2	1
BF638R isolated at 2 μg/mL MTZ	4	2	2	1
BF638R isolated at 4 μg/mL MTZ	8	8	4	4
BF638R isolated at 8 μg/mL MTZ	16	16	4	4
BF638R isolated at 16 μg/mL MTZ	32	32	4	4
*B. fragilis* ADB77	0.5	0.5	2	1
*B. fragilis* BF8	16	2	2	1
*B. fragilis* ATCC 25285	1	0.5	1	1
*B. thetaiotaomicron* VPI 5482	1	1	1	1
*B. vulgatus* ATCC 8482	1	0.5	1	0.0625
*B. fragilis* 638R mutant strains				
*Δpir1::tetQ*	0.5	0.5	2	1
*Δpir2::cfxA*	0.5	0.5	2	1
*Δpir1::tetQ Δpir2::cfxA*	0.5	0.5	2	1
*Δpir1::tetQ/pir1* ^*+*^	0.25	0.125	2	0.5
*Δpir2::cfxA/pir2* ^*+*^	0.25	0.125	2	0.5
*Δpir1::tetQ Δpir2::cfxA/pir1* ^*+*^	0.25	0.125	2	0.5
*Δpir1::tetQ Δpir2::cfxA/pir2* ^*+*^	0.25	0.125	2	0.5
BF638R *pir1* ^*+*^	0.5	0.0625	2	1
BF638R *pir2* ^*+*^	0.5	0.0625	2	1
*ΔPFOR::tetQ*	1	1	2	1
*Δkor1AB*	0.5	0.5	2	1
*Δkor2AEBG*	1	1	4	2
*ΔPFOR::tetQ Δkor1AB*	1	1	2	2
*ΔpoxB*	1	0.5	4	1
*ΔPFOR::tetQ ΔpoxB*	1	1	4	1
*Δkor1AB ΔpoxB*	1	0.5	2	1
*ΔPFOR::tetQ Δkor1AB ΔpoxB*	1	1	2	2
*ΔPFOR::tetQ Δkor2AEBG*	1	1	2	2
*ΔPFOR::tetQ Δkor1AB Δkor2AEBG*	1	1	4	2
*pyc::pFD516*	0.5	0.5	1	1
*ΔfrdC247*	0.25	0.25	1	0.5
*ΔfrdB260*	0.25	0.0625	0.5	0.5
*ΔfrdC/frdC* ^*+*^	0.5	0.25	2	1
*ΔfrdB260/frdB* ^*+*^	0.5	0.25	1	1
*ΔcitS Δicd ΔacnA*	1	1	4	2
*Δkor2AEBG ΔcitS Δicd ΔacnA*	1	1	2	2
*ΔPFOR::tetQ Δkor2AEBG ΔcitS Δicd ΔacnA*	4	2	2	1
*ΔPFOR::tetQ Δkor1AB Δkor2AEBG ΔcitS Δicd ΔacnA*	4	2	4	2
Hydrogen peroxide resistant strain, *hpr*	0.5	0.25	2	1
*ΔkatB::tetQ*	0.5	0.25	2	1
*ahpCF:pFD516*	0.5	0.25	2	1
*ahpF::pFD516*	0.5	0.25	2	1
*ΔahpC::tetQ*	0.5	0.25	2	1
*ΔftnA:tetQ*	0.5	0.25	2	1
*Δbfr::cfxA*	0.5	0.25	2	1
*Δdps::tetQ*	0.5	0.25	2	1
*Δbfr::cfxA ΔftnA::tetQ*	0.5	0.25	2	1
*Δbfr Ddps*	0.25	0.25	2	0.5
*Δbfr::cfxA Δdps::tetQ/bfr* ^*+*^	0.25	0.25	1	0.5
*Δbfr::cfxA Δdps::tetQ/dps* ^*+*^	0.25	0.125	1	0.5
*ΔfnrA::tetQ*	0.5	0.5	2	1
*ΔnrfA::tetQ*	0.5	0.5	2	1
*ΔfnrC*	0.5	0.5	1	1
*ΔrelA ΔspoT*	0.5	0.5	2	1
*ΔtrxB::cfxA*	0.25	0.25	1	0.5
*ΔtrxC ΔτrxD::cfxA ΔtrxE ΔtrxF ΔtrxG*	0.5	0.5	2	2
*ΔtrxC ΔtrxD::cfxA ΔτrxE ΔtrxF ΔtrxG ΔoxyR::tetQ*	0.5	0.25	2	0.5

*Note*: Anaerobic: Inoculated plates were immediately incubated anaerobically. O_2_ exposure: Duplicate plates were incubated for 16–20 h in an aerobic incubator at 37℃ before anaerobic incubation at 37℃.

In contrast, the MICs for MTZ and AMIX in the *Δpir1*, *Δpir2*, and *Δpir1 Δpir2* double mutant strains were not altered in cultures exposed to oxygen compared to the parent strain as determined by the agar dilution assay (Table 2). However, the complemented *pir1* and *pir2* mutant strains were twofold more sensitive to MTZ (MIC 0.25 μg/mL) in anaerobic cultures, and fourfold more sensitive to MTZ (MIC 0.125 μg/mL) in cultures exposed to oxygen compared to the parent strain in agar dilution method, respectively (Table 2). The MTZ MIC of the parent strain overexpressing *pir1* or *pir2* genes remained unaltered in anaerobic conditions (MIC 0.5 μg/mL) but when these strains were exposed to oxygen, they showed an eightfold increase in MTZ sensitivity (MIC 0.0625 μg/mL) compared to the parent strain (Table 2). In addition, the genetically complemented strains expressing *pir1* or *pir2* genes did not affect AMIX MIC anaerobically, but it caused a twofold increase in AMIX sensitivity (0.5 μg/ml) in oxygen‐exposed cultures compared to the parent strain. The parent strain overexpressing *pir1* or *pir2* genes showed no changes in amixicile MIC anaerobically or following oxygen exposure (Table 2).

Altogether, these findings support the roles of Pir1 and Pir2 in altering susceptibility to MTZ or AMIX though there are some inconsistencies between disc inhibition and agar dilution assays. Because pirin proteins may modulate the derepression or suppression of different metabolic pathways, it is a challenge to point out which metabolic activities could contribute to changes in susceptibility to MTZ or AMIX. Therefore, mutants defective in a variety of cellular metabolic functions were used to examine their antimicrobial susceptibility.

### 3.6 Different functional metabolic mutants were tested for susceptibility to MTZ and AMIX

**Figure 5:**
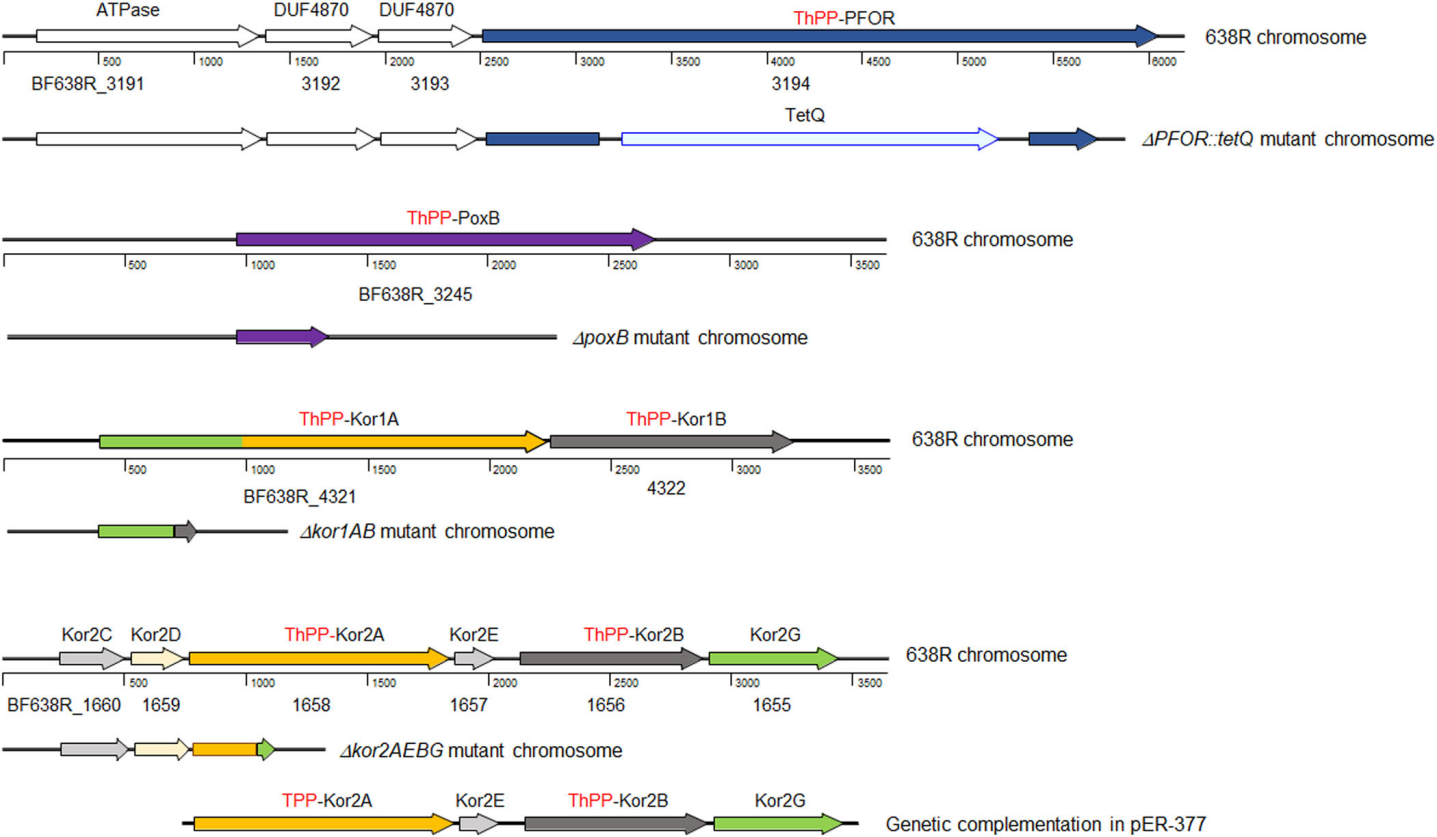
Schematic representation of *B. fragilis* 638R chromosomal regions for PFOR, PoxB, Kor1AB, and Kor2AEBG as shown in the panels. Each locus tag is depicted below the respective deduced ORF symbolized by an arrow. The designation of the predicted peptide product is depicted above each open arrow gene region respectively. The Arrow direction depicts the transcription orientation. Arrows filled with color represent the functional annotation group assigned to PFOR (dark blue), PoxB (purple), KorA (orang), KorB (dark gray), KorG (light green), KorD (gold) or, KorC (light gray) orthologs, respectively. The deletion construct representation of each chromosomal region mutant is shown below the native chromosome region, respectively. The DNA fragment containing the promoterless *kor2AEBG* genes cloned into the expression vector pFD340 (pER‐377) was used for genetic complementation studies. ATPase, predicted ATPase AAA+ superfamily; DUF4870, putative membrane protein of unknown function; Kor1A, 2‐ketoglutarate ferredoxin oxidoreductase subunit α (CBW24739); Kor1B, 2‐ketoglutarate ferredoxin oxidoreductase subunit β (CBW24740); Kor2A, 2‐ketoglutarate ferredoxin oxidoreductase subunit α (CBW22186); Kor2B, 2‐ketoglutarate ferredoxin oxidoreductase subunit β (CBW22184); Kor2C, conserved hypothetical protein containing tetratricopeptide repeat (CBW22188); Kor2D, ferredoxin, 2‐ketoglutarate‐acceptor oxidoreductase subunit δ (CBW22187); Kor2E, hypothetical protein (CBW22185); Kor2G, 2‐ketoglutarate ferredoxin oxidoreductase subunit γ (CBW22183); PFOR, pyruvate:ferredoxin oxidoreductase (GenBank accession number CBW23670); PoxB, putative pyruvate dehydrogenase (CBW23720); ThPP, thiamine diphosphate cofactor.

**Figure 6:**
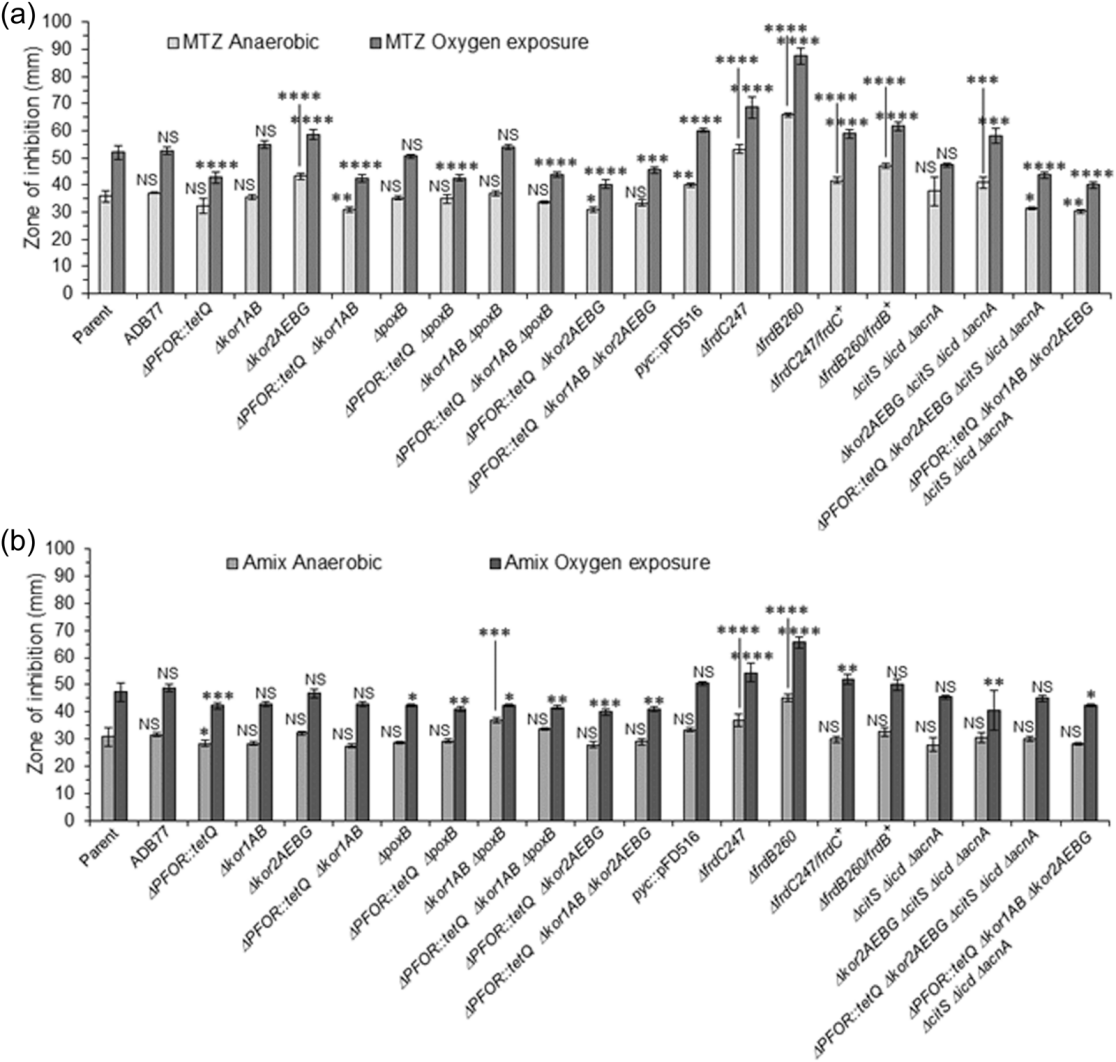
Disc diffusion assay sensitivity for (a) metronidazole (MTZ) and (b) amixicile (AMIX). *B. fragilis* strains are depicted in each panel. Each bar represents the average zone inhibition (mm) of at least three independent biological replicates. Vertical error bars denote the standard deviation of the means from two independent experiments in triplicate. The significance of the *p* value was calculated by one‐way ANOVA followed by multiple comparisons using Dunnett and Bonferroni tests. Only groups with statistical significance in both tests are reported. *p* < 0.05 (*), *p* < 0.01 (**), *p* < 0.001 (***), and *p* < 0.0001 (****). NS, not significant.

A series of mutant strains available in our laboratory collection encompassing different metabolic and physiological functions such as: ThPP‐binding 2‐ketoacid oxidoreductases, carboxylases, TCA cycle enzymes, oxidative and redox stress responses, stringent response, nitrate reductase ortholog, anaerobic Fnr‐like regulators, and iron‐storage proteins listed in Table 1 were tested for MTZ and AMIX susceptibility. The genomic organizations and the deletion construct diagrams of four members of the ThPP‐dependent 2‐ketoacid oxidoreductases: the *PFOR*, *kor1AB*, and *kor2CDAEBG* which use ferredoxin as electron acceptor, and the putative inner membrane enzyme that catalyzes the oxidative decarboxylation of pyruvate, *poxB*, used in this study are shown in Figure 5. The results are presented in Table 2, Figure 6, and Supplemental File S7. Of particular interest, the *Δkor2AEBG*, *ΔpoxB* and *ΔPFOR::tetQ* single mutant strains, the *ΔPFOR::tetQ Δkor2AEBG* double mutant, the *ΔPFOR::tetQ Δkor1AB ΔpoxB* triple‐mutant, and the *ΔPFOR::tetQ Δkor1AB Δkor2AEBG* triple mutant strains showed twofold increase in MTZ resistance (MIC 1 μg/ml) compared to the parent strain (MIC 0.5 μg/ml). No significant changes occurred in the *Δkor1AB* and *pyc* single mutant strains as determined by agar dilution assay (Table 2). In contrast, the *Δkor2AEBG* mutant was statistically more sensitive to MTZ in anaerobic cultures and cultures exposed to oxygen compared to the parent strain as determined by disc inhibition assay (Figure 6). The *Δkor2AEBG ΔcitS Δicd ΔacnA* quadruple mutant sensitivity to MTZ was not statistically significantly different from *Δkor2AEBG* single mutant as determined by disc inhibition assays or agar dilution method (Table 2, Figure 6). Interestingly, the *ΔPFOR::tetQ Δkor2ABG ΔcitS Δicd ΔacnA* quintuple mutant had an eightfold increase in MTZ resistance (MIC 4 μg/mL) anaerobically and fourfold increase (MIC 2 μg/mL) following exposure to oxygen compared to the parent strain (Table 2). The *ΔcitS Δicd ΔacnA* triple mutant was twofold more resistant to MTZ (MIC 1 μg/mL) and to AMIX (4 μg/mL, 2 μg/mL) in anaerobic cultures and cultures exposed to oxygen compared to the parent strain, respectively (Table 2). In contrast, the *ΔcitS Δicd ΔacnA* triple mutant susceptibility to MTZ or AMIX was not significantly altered as determined by disc inhibition assay (Figure 6). Insertional mutation of the pyruvate carboxylase biotin‐containing subunit gene, *pyc*, also increased susceptibility to MTZ anaerobically or in cultures exposed to oxygen as determined by disc inhibition assay but not in agar dilution assay. It is unclear why some strains showed conflicting susceptibility between disc inhibition and agar dilution methods determination.

The *ΔPFOR::tetQ* mutant was statistically more resistant to AMIX in anaerobic cultures and cultures exposed to oxygen compared to the parent strain as determined by disc inhibition assays (Figure 6b) but changes in AMIX susceptibility were not observed in agar dilution assay (Table 2). Interestingly, the *Δkor1AB ΔpoxB* was statistically more sensitive to AMIX anaerobically but more resistant to AMIX in cultures exposed to oxygen as determined by disc inhibition assays (Figure 6b) whereas no significant changes in susceptibility were seen in MIC values (Table 2). The *ΔpoxB*, *ΔPFOR:tetQ ΔpoxB*, *ΔPFOR:tetQ Δkor1AB ΔpoxB*, *ΔPFOR::tetQ Δkor2AEBG*, *ΔPFOR::tetQ Δkor1AB Δkor2AEBG*, *Δkor2AEBG ΔcitS Δicd ΔacnA*, and *ΔPFOR::tetQ Δkor1AB Δkor2AEBG ΔcitS Δicd ΔacnA* mutants were statistically more resistant to AMIX only in cultures exposed to oxygen, but no significant changes in susceptibility occurred in anaerobic cultures compared to the parent strain as determined by disc inhibition assays (Figure 6b). However, the *ΔpoxB*, *ΔPFOR:tetQ ΔpoxB*, *ΔPFOR::tetQ Δkor1AB Δkor2AEBG*, and *ΔPFOR::tetQ Δkor1AB Δkor2AEBG ΔcitS Δicd ΔacnA* were twofold more resistant to AMIX in anaerobic conditions as determined by agar dilution assays (Table 2).

Among the mutants involved in the TCA cycle shown in Figure 6, it was the deficiency in the fumarate reductase subunits, *frdB* and *frdC* that caused the highest increase in MTZ and AMIX sensitivity. The *ΔfrdB* mutant had a twofold increase in MTZ susceptibility (MIC 0.25 μg/mL) compared to the parent strain. Remarkably, following oxygen exposure, the Δ*frdB* mutant had an eightfold increase in MTZ susceptibility (MIC 0.0625 μg/mL) (Table 2). The lack of the *frdB* or the *frdC* subunits in increasing MTZ and AMIX susceptibility is also clearly noticeable in the disc inhibition assays (Figure 6). However, the genetically complemented strains, *ΔfrdB260/frdB* ^*+*^ and *ΔfrdC/frdC* ^*+*^ brought MTZ susceptibility close, but not identical, to the parent strain levels as determined by MIC and disc inhibition assays (Table 2 and Figure 6). It is unclear why there was a significant difference in MTZ and AMIX susceptibility between the two fumarate reductase deficient strains, *ΔfrdB260* and *ΔfrdC247* though *ΔfrdC247* is reverted to *thyA* ^*+*^. It is unlikely that this was due to the addition of succinate and thymine supplements to the media since the susceptibility of their parent strain ADB77 (638R *ΔthyA* isogenic) used as a control was not altered compared to the 638R strain. Perhaps, unidentified intrinsic factors may exist though there are no significant differences in molar growth yield or generation time between *ΔfrdB* and *ΔfrdC* strains (Baughn & Malamy, 2003).

Overall, these findings appear to associate deficiencies in the oxidative TCA cycle branch such as lack of PFOR with significantly more resistance to MTZ while deficiencies in the reductive TCA branch genes such as *frdB*, and *frdC* with significantly more sensitivity to MTZ. These alterations in the oxidative or reductive balance of the central metabolism could ultimately lead to dysregulation of the NADH/NAD^+^ redox processes and cellular bioenergetics. To test this assumption, inhibitors of *B. fragilis* NADH:electron acceptor transport coupling system (ETS) for fumarate reductase reduction of fumarate to succinate as described previously (Harris & Reddy, 1977) were used as mentioned below.

The nitrite reductase deficient strain, *ΔnirfA::tetQ*, was significantly more sensitive to MTZ anaerobically but not in cultures exposed to oxygen (Supplemental File S7A) while it did not alter sensitivity to AMIX compared to the parent strain (Supplemental File S7C). Strains deficient in thiol/disulfide redox homeostasis such as *ΔtrxB::tetQ*, and *ΔtrxC ΔtrxD::cfxA ΔtrxE ΔtrxF ΔtrxG ΔoxyR::tetQ* sextuple mutants were significantly more sensitive to MTZ anaerobically but not in oxygen exposed cultures. The *ahpCF*::pFD516 and *ahpF:*:pFd516 insertional mutants were significantly more sensitive to MTZ in both anaerobic cultures and cultures exposed to oxygen compared to the parent strain. The sensitivity of the *ΔahpC::tetQ* single mutant was not altered compared to the parent strain as determined by disc inhibition assays (Supplemental File S7A). This suggests that the AhpF subunit of the alkyl hydroperoxide reductase may contribute to the resistance to MTZ in the parent strain. The *ΔtrxB::tetQ* strain was significantly more sensitive to AMIX in both anaerobic growth and cultures exposed to oxygen while the *ΔtrxC ΔtrxD::cfxA ΔtrxE ΔtrxF ΔtrxG ΔoxyR::tetQ* sextuple mutants were more resistant to AMIX in anaerobic cultures (Supplemental File S7C). The MICs for MTZ and AMIX determined for the strains mentioned in Supplemental File S7 are shown in Table 2.

Moreover, this study shows that members of the ferritin superfamily, FtnA Dps and Dps‐like protein, Bfr play a significant role in increasing sensitivity to MTZ and AMIX (Supplemental File S7B,D). The *Δdps::tetQ Δbfr::cfxA* double mutant was significantly more susceptible to MTZ and AMIX in anaerobic cultures and upon oxygen exposure compared to the *ΔftnA*, *Δdps* and *Δbfr* single mutant strains as determined by disc inhibition assays (Supplemental File S7B,D). This indicates that there might be a synergistic effect of *Dps* and *Bfr* contributing to MTZ and AMIX antibiotic resistance. Deletion of the *ftnA* gene had a modest increase in AMIX sensitivity in oxygen‐exposed cultures. However, it was the genetic complementation of the *Δdps::tetQ Δbfr::cfxA* double mutant strain with *bfr* ^*+*^ or *dps* ^*+*^ genes in the strains *Δdps::tetQ Δbfr::cfxA bfr* ^*+*^ and *Δdps::tetQ Δbfr::cfxA dps* ^*+*^ that caused a significant increase in susceptibility to MTZ and AMIX in anaerobic cultures and cultures exposed to oxygen compared to the single mutants and the parent strain, respectively. It is unclear why the constitutive expression of *dps* or *bfr* genes enhanced the sensitivity of the *Δdps::tetQ Δbfr::cfxA* double mutant strain to MTZ and AMIX instead of bringing the mutant sensitivity closer to the parent strain levels. It is unlikely that this is simply due to lowering intracellular free ferrous iron content by increasing iron‐stored levels. This contradicts a previous study showing that deficiency in the ferrous uptake system FeoAB increases MTZ resistance (Veeranagouda et al., 2014). However, the possibility that changes in iron homeostasis caused alterations in metabolic pathways activities that affect MTZ susceptibility cannot be disregarded.

Because several metabolic pathways, that can with different intensities alter *B. fragilis* 638R susceptibility to MTZ and AMIX, have reduction/oxidation functions, we cannot rule out that they are linked to dysregulation of ETS and redox balance as a common determining factor in MTZ and AMIX susceptibility. Therefore, we investigated whether ETS inhibitors and redox cycling agents could play a role in MTZ and AMIX susceptibility.

### 3.7 The effect of ETS inhibitors on MTZ and AMIX susceptibility

**Figure 7:**
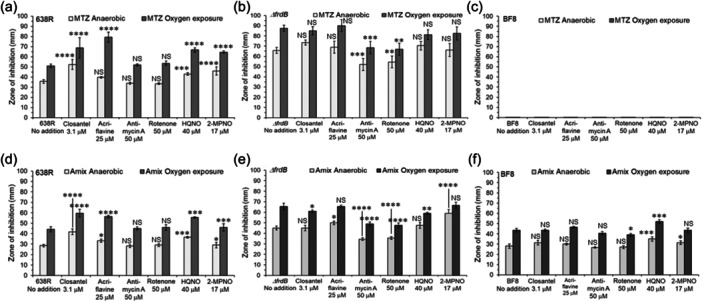
Disc diffusion assay sensitivity of *B. fragilis* strains to metronidazole (MTZ), panels A,B,C or for Amixicile (AMIX), panels D,E,F. A and D: Parent (*B. fragilis* 638R). B and E: *ΔfrdB* (derived from ADB77, isogenic BF638R *thy* ^*‐*^ strain). C and F: *B. fragilis* BF8 strain (*nimB* ^*+*^). Strain designations are depicted in each panel. For these experiments, 20 mM succinate and 50 μg/ml thymine were added to the BHIS media. Each bar represents the average zone of inhibition (mm) of at least three independent biological replicates. Vertical error bars denote the standard deviation of the means from two independent experiments in triplicate. The significance of the *P* value was calculated by one‐way ANOVA followed by multiple comparisons using Dunnett and Bonferroni tests. Only groups with statistical significance in both tests are reported. *p* < 0.05 (*), *p* < 0.01 (**), *p* < 0.001 (***), and *p* < 0.0001 (****). NS, not significant.

The non‐inhibitory concentrations of the ETS inhibitors: acriflavine, rotenone, 2‐heptyl‐hydroxyquinoline‐N‐oxide (HQNO), and antimycin A used in the disc inhibition assays are mentioned in the text and Materials and Methods section. Closantel, a halogenated salicylanilide antimicrobial whose mechanism of action is not completely understood but decouples oxidative phosphorylation and leads to inhibition of ATP synthesis (Rajamuthiah et al., 2014; Tran et al., 2016; Van Den Bossche et al., 1979; Williamson & Metcalf, 1967), and 2‐Mercaptopyridine‐N‐oxide (2‐MPNO), an NADH:fumarate reductase inhibitor (Turrens et al., 1999) were also included in this study. The BF638R strain was statistically more sensitive to both MTZ and AMIX in the presence of closantel, acriflavine, HQNO, and 2‐MPNO under both anaerobic and oxygen‐exposed conditions except that acriflavine did not change susceptibility to MTZ anaerobically (Figure 7a,d). Antimycin A and rotenone did not cause statistically significant changes in either MTZ or AMIX susceptibility compared to no addition control as determined by disc inhibition assays.

The *ΔfrdB* mutant strain, which is significantly more sensitive to MTZ and AMIX than to the parent strain (Figure 6), did not show significant susceptibility changes to MTZ in the presence of closantel, acriflavine, HQNO, or 2‐MPNO compared to no addition control (Figure 7b). However, in the presence antimycin A and rotenone, the *ΔfrdB* mutant was more resistant to MTZ and AMIX compared to no addition control (Figure 7b,e). In the presence of closantel or HQNO, the *ΔfrdB* mutant was more resistant to AMIX in cultures exposed to oxygen but not in anaerobic cultures. However, in the presence of acriflavine or 2‐MPNO, it was more sensitive to AMIX in anaerobic cultures but did not significantly change susceptibility in cultures exposed to oxygen compared to no addition control (Figure 7e). To determine if ETS could affect MTZ resistance in a strain carrying an MTZ resistance *nim* gene ortholog, the BF8 strain carrying *nimB* was tested. The presence of ETS did not affect the BF8 resistance to MTZ either (Figure 7c). The BF8 sensitivity to AMIX did not significantly change in the presence of closantel, acriflavine, or antimycin A compared to control (Figure 7f). The BF8 strain was significant more sensitive to AMIX in the presence of HQNO in anaerobic cultures and cultures exposed to oxygen. There was a modest increase in AMIX sensitivity in the presence of 2‐MPNO in anaerobic culture but not in culture exposed to oxygen, and a modest increase in resistance to AMIX in the presence of rotenone in oxygen‐exposed cultures (Figure 7f).

### 3.8 The effect of redox cycling agents on MTZ susceptibility

**Figure 8:**
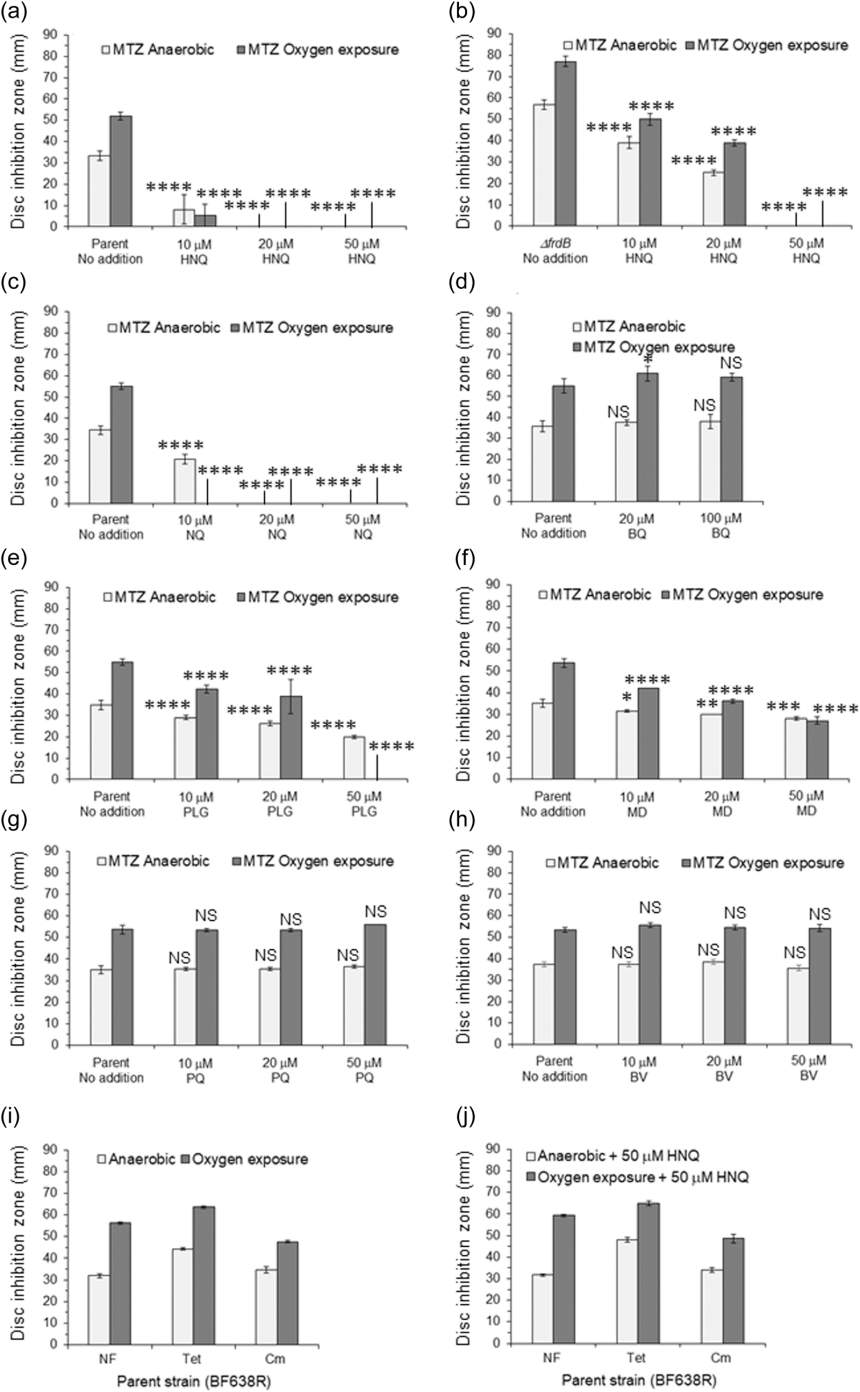
Disc diffusion assays. Susceptibility of *B. fragilis* 638R strain (Parent) and its isogenic *ΔfrdB* deletion mutant to metronidazole (MTZ). Panels (a) and (b): in the presence of 2‐hydroxy‐1,4‐napththoquinone (HNQ). Panel (c): in the presence of 1,‐4‐naphthoquinone (NQ). Panel (d): in the presence of 1,4‐benzoquinone (BQ). Panel (e): in the presence of plumbagin (PLG). Panel (f): in the presence of menadione (MD). Panel (g): in the presence of paraquat (PQ). Panel (h): in the presence of benzyl‐viologen (BV). Panels i and j: Susceptibility of *B. fragilis* 638 R strain to nitrofurantoin (NT), tetracycline (Tet), or chloramphenicol (Cm) exposed to oxygen with no addition (i) or addition of 50 μM HNQ (j). Each bar represents the average zone inhibition (mm) of at least three independent biological replicates. Vertical error bars denote the standard deviation of the means from two independent experiments in triplicate. Panels a–h: The significance of the *p* value was calculated by one‐way ANOVA followed by multiple comparisons using Dunnett and Bonferroni tests. Only groups with statistical significance in both tests are reported. *p* < 0.05 (*), *p* < 0.01 (**), *p* < 0.001 (***), and *p* < 0.0001 (****). NS, not significant. For panels i and j, no statistical analyzes were performed.

In contrast to the effects of ETS, when the redox cycling agents 1,4‐naphthoquinone (NQ) and 2‐hydroxy‐1,4‐naphthoquinone (HNQ), which are analogs to menadione (2‐methyl‐1,4‐naphthoquinone) were added to the culture media at 10 to 20 μM, they abolished *B. fragilis* 638 R sensitivity to MTZ as determined by agar dilution and disc inhibition assays (Figure 8a,c and Table 3). Plumbagin (5‐hydroxy‐2‐methyl‐1,4‐naphthoquinone) and menadione caused lesser effect (Figure 8e,f) while there were no significant effects observed with 1,4‐benzoquinone, paraquat (methyl viologen), or benzyl viologen compared to no addition control as determined by disc inhibition assays (Figure 8d,g,h). The effect of 10 μM and 20 μM of HNQ on the *ΔfrdB* mutant resistance to MTZ was significantly less than the parent strain (Figure 8a,b). Moreover, the addition of HNQ did not alter the parent strain *B. fragilis* 638R susceptibility to other antimicrobials such as nitrofurantoin, tetracycline, or chloramphenicol compared to no addition control, respectively (Figure 8i,j). The effect of redox cycling agents on MTZ susceptibility was not due to bacterial growth inhibition because they did not significantly affect the CFU counts in anaerobic conditions or cultures exposed to oxygen at concentrations equal to or above the ones used in this study (Supplemental File S8).

**Table 3:** Metronidazole minimal Inhibitory concentration (MIC μg/mL) for *B. fragilis* 638R in BHI media containing 5 μg/mL hemin (l‐cysteine was omitted) supplemented with 2‐hydroxy‐1,4‐naphthoquinone, or 1,4‐naphthoquinone.

Media supplement*/*MTZ MIC μg/mL	Anaerobic	O_2_ exposure
Control (No addition)	0.5	0.5
2‐hydroxy‐1,4‐Naphthoquinone 10 μM	8	4
2‐hydroxy‐1,4‐Naphthoquinone 50 μM	16	8
2‐hydroxy‐1,4‐Naphthoquinone 100 μM	32	32
1,4‐Naphthoquinone 10 μM	2	2
1,4‐Naphthoquinone 50 μM	16	16
1,4‐Naphthoquinone 100 μM	32	32

### 3.9 No crossed MTZ resistance and AMIX susceptibility

**Figure 9:**
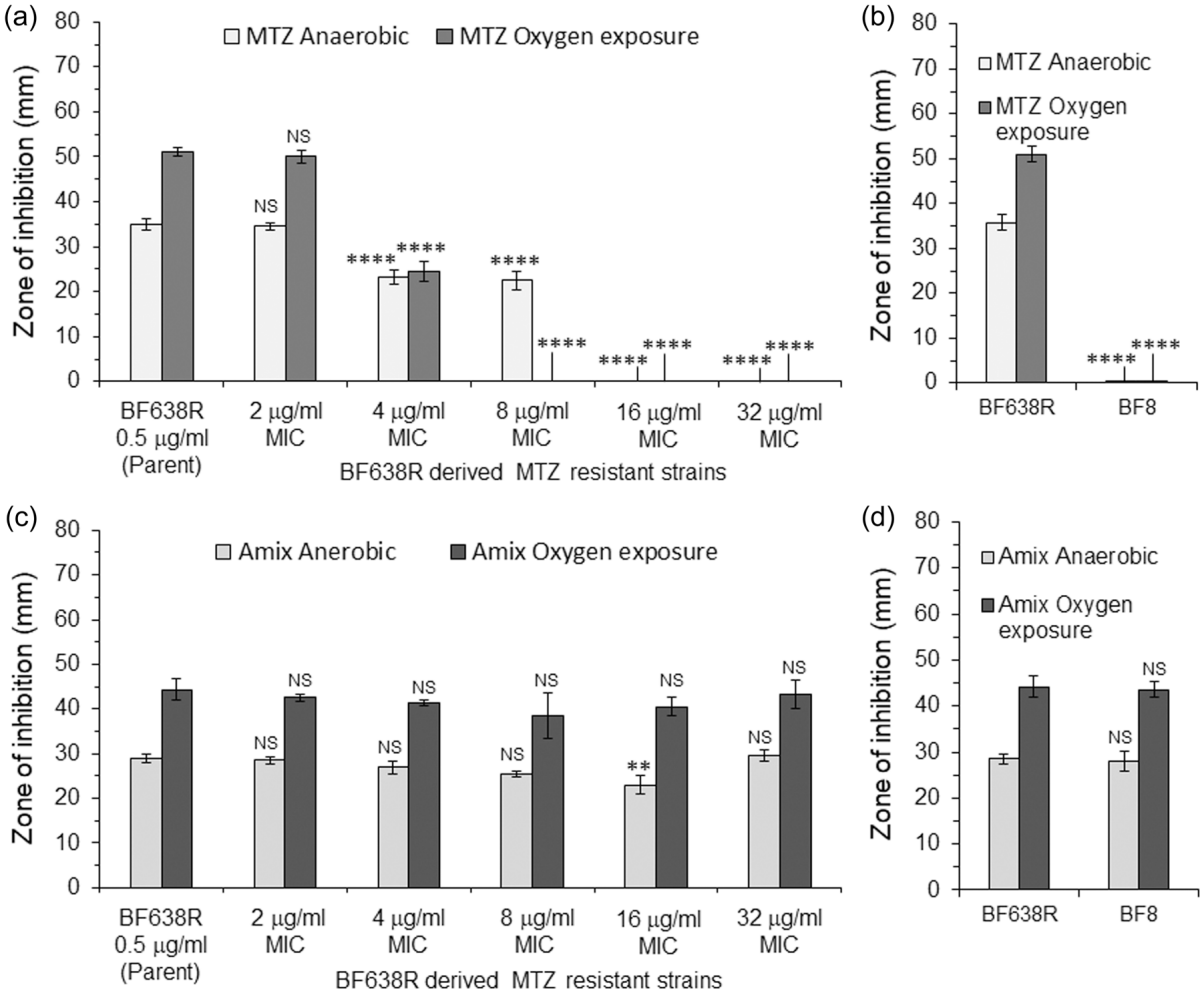
Disc diffusion assay sensitivity for (a‐b) metronidazole (MTZ) and (c‐d) Amixicile (AMIX). *B. fragilis* strains are depicted in each panel. Each bar represents the average zone inhibition (mm) of at least three independent biological replicates. Vertical error bars denote the standard deviation of the means from two independent experiments in triplicate. In panels a and c, the significance of the *p* value was calculated by one‐way ANOVA followed by multiple comparisons using Dunnett and Bonferroni tests. Only groups with statistical significance in both tests are reported. *p* < 0.05 (*), *p* < 0.01 (**), *p* < 0.001 (***), and *p* < 0.0001 (****). NS: Not significant. In Panels B and D, the significance of the *p* value was calculated using an unpaired *t‐test* (parametric and two‐tailed) to compare the means of the two groups.

There is no report of AMIX‐resistant mutant strains and this led us to test whether resistance to MTZ could alter susceptibility to AMIX. MTZ resistant strains were obtained by isolating random strains grown in increasing MTZ concentrations at 2, 4, 8, and 16 μg/mL. The findings showed that BF638R_(2 μg/mL)_ (MIC 4 μg/mL) showed no increase in AMIX resistance compared to the parent strain (Table 2). The BF638R_(4 μg/mL)_ (MIC 8 μg/mL), BF638R_(8 μg/mL)_, (MIC 16 μg/mL), and BF638R_(16 μg/mL)_ (MIC 32 μg/mL) strains only showed a twofold increase in AMIX MIC (4 μg/mL) (Table 2). In Figure 9a,b, a comparison is shown for the random MTZ‐induced resistant strains and the BF8 MTZ resistant strain using disc inhibition assays, respectively. In addition, the evaluation of the susceptibility to AMIX in the random MTZ‐induced resistant strains and the BF8 strain is shown in Figure 9c,d. The findings show MTZ resistance mechanism did not have any significative effect on AMIX susceptibility in anaerobic cultures or cultures exposed to oxygen, except for a modest increase in AMIX resistance in the BF638R resistant to MTZ at 16 μg/ml in anaerobic cultures but not in cultures exposed to oxygen as determined by disc inhibition assays (Figure 9c). There were no statistically significant differences in AMIX susceptibility between the BF638R and BF8 strains as determined by disc inhibition assays and agar dilution method (Figure 9d, Table 2).

### 3.10 AMIX has antimicrobial activity against *B. fragilis* 638R in an in vivo intra‐abdominal infection model

**Figure 10:**
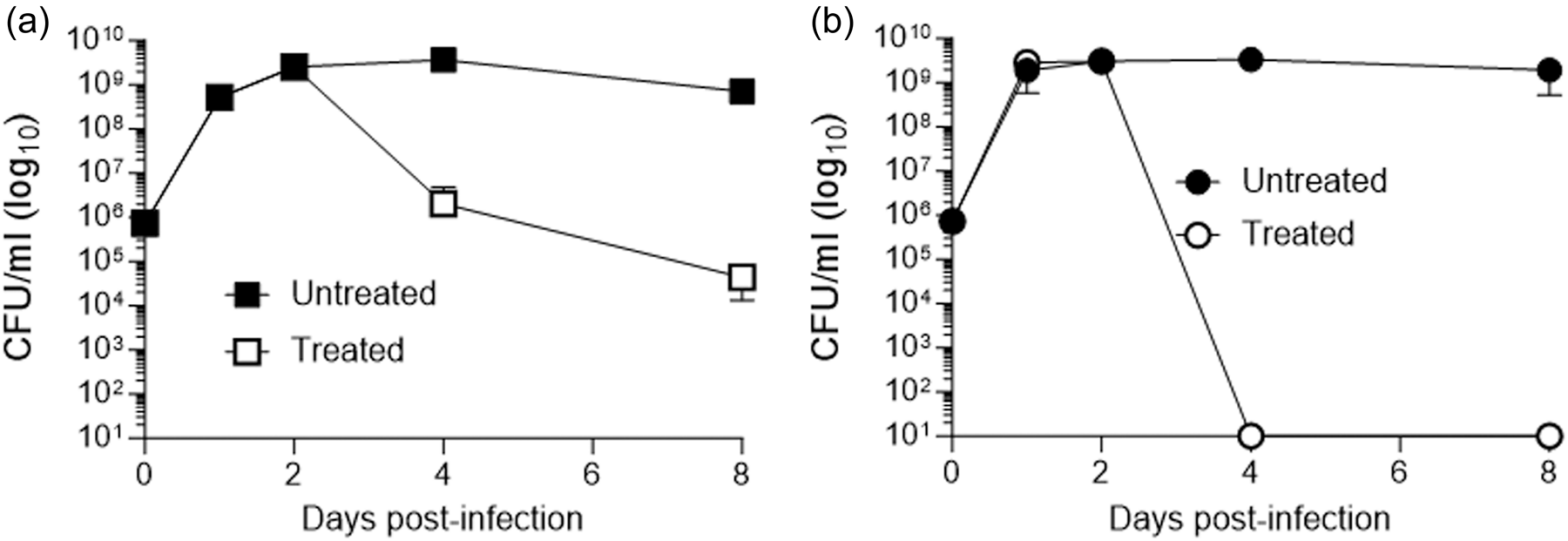
Survival of *B. fragilis* 638R in rat tissue cage infection following AMIX treatment at 20 mg/Kg/day via intraperitoneal (a) or 0.5 mg/intra‐cage/day (b). AMIX administration started on Day 1 through Day 7 postinfection. Bacteria were grown overnight in BHIS medium and diluted in PBS to approximately 1 × 10^5^ CFU/mL. Four milliliters of the suspension were inoculated into the intraperitoneal tissue cage. Fluid samples were aspirated at time points for CFU counts as described in Materials and Methods. Tissue cage fluid was aspirated at Days 1, 2, 4 and 8 postinfection. Data are expressed as the mean CFU per milliliter of intra‐abdominal tissue cage fluid from three rats. The standard errors of the means (SEM) are denoted by vertical error bars. The detection limit of 1 × 10^1^ CFU/mL.

The tissue cage model of intra‐abdominal infection was used to examine if AMIX would have antimicrobial activity against *B. fragilis in vivo.* After inoculating *B. fragilis* 638R into the intra‐abdominal tissue cage, the CFU/mL counts decreased by nearly three log‐fold at Day 4 postinfection and over four log‐fold at Day 8 postinfection in rats receiving AMIX IP compared to the CFU/mL of untreated rats receiving saline (Figure 10a). However, the viability of *B. fragilis* in rats receiving intra‐cage administration of AMIX was completely lost (below detection) at Day 4 postinfection, and the CFU counts remained undetectable at Day 8 postinfection compared to untreated control receiving saline (Figure 10b). Taken together, these findings show that AMIX, a narrow spectrum antimicrobial designed to replace and inhibit thiamine‐binding in ThPP‐binding oxidoreductases, such as PFOR, is a potential alternative antimicrobial for *B. fragilis* infection. However, it remains to be determined if AMIX is effective in inhibiting *B. fragilis* MTZ‐resistant strains in vivo.

Because lack of PFOR does not affect *B. fragilis* 638R growth rate and has a modest effect on MTZ and AMIX susceptibility compared to the parent strain, experiments were carried out to find out if other members of the ThPP‐binding 2‐ketoacid:ferredoxin oxidoreductases might play an essential role in *B. fragilis* anaerobic metabolism and physiology.

### 3.11 The ThPP‐binding 2‐ketoglutarate ferredoxin oxidoreductase subunits Kor2AEBG are essential for *B. fragilis* growth

**Figure 11:**
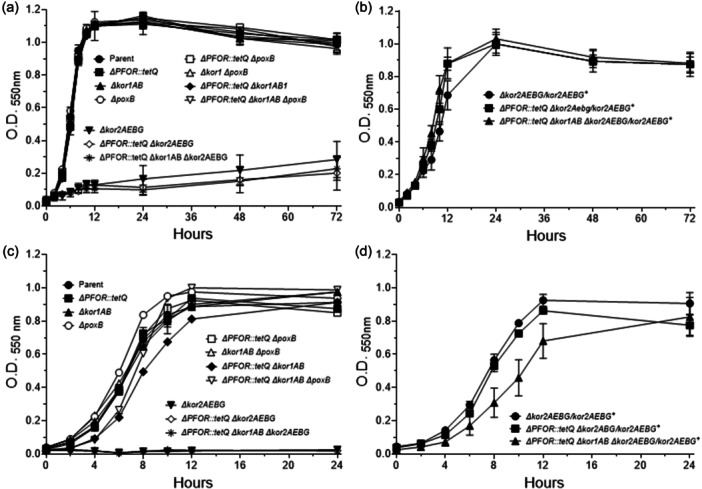
Growth of *B. fragilis* mutant strains in supplemented brain‐heart infusion (BHIS) media (Panels a and b), or in chemically defined media with glucose (DM) (panels c and d). Strain designations are depicted for each panel. Media preparations and compositions are described in the Materials and Methods section. Panels b and d show the *Δkor2AEBG* genetic complemented strains with pER‐377 grown in BHIS (b) or defined medium (d), respectively.

Deletion of the *kor2AEBG* genes, contained in the putative *kor2CDAEBG* operon, in the *Δkor2AEBG*, *ΔPFOR::tetQ Δkor2AEBG*, and *ΔPFOR::tetQ Δkor1AB Δkor2AEBG* mutant strains caused a severe growth defect in rich media and completely abolished growth in minimally defined media (Figure 11a,c). This indicates that the deficiency of *kor2AEBG* genes alone is responsible for the growth defect. The *ΔPFOR::tetQ*, *Δkor1AB*, *ΔpoxB*, *ΔPFOR::tetQ ΔpoxB*, *Δkor1AB ΔpoxB*, *ΔPFOR::tetQ Δkor1AB*, and the *ΔPFOR::tetQ Δkor1AB ΔpoxB* mutant strains did not show significant growth defect compared to the parent strain. The genetic complementation of the strains carrying the *Δkor2AEBG* deletion with the *kor2AEBG* native genes completely restored growth to the parent strain levels in both BHIS media and in minimally defined media (Figure 11b,d). However, the role of the *kor2AEBG* genes in the reductive formation of the 2‐KG *B. fragilis* TCA cycle is not well defined. In addition, we cannot rule out that *kor1AB* genes could be involved in the synthesis of 2‐KG because the *kor2AEBG* was shown to play a multifunctional role as described below.

### 3.12 Supplementation with dimethyl‐2‐ketoglutarate (dM‐2KG) restores the growth of the *Δkor2AEBG* mutant in complex media but not in chemically defined media

**Figure 12:**
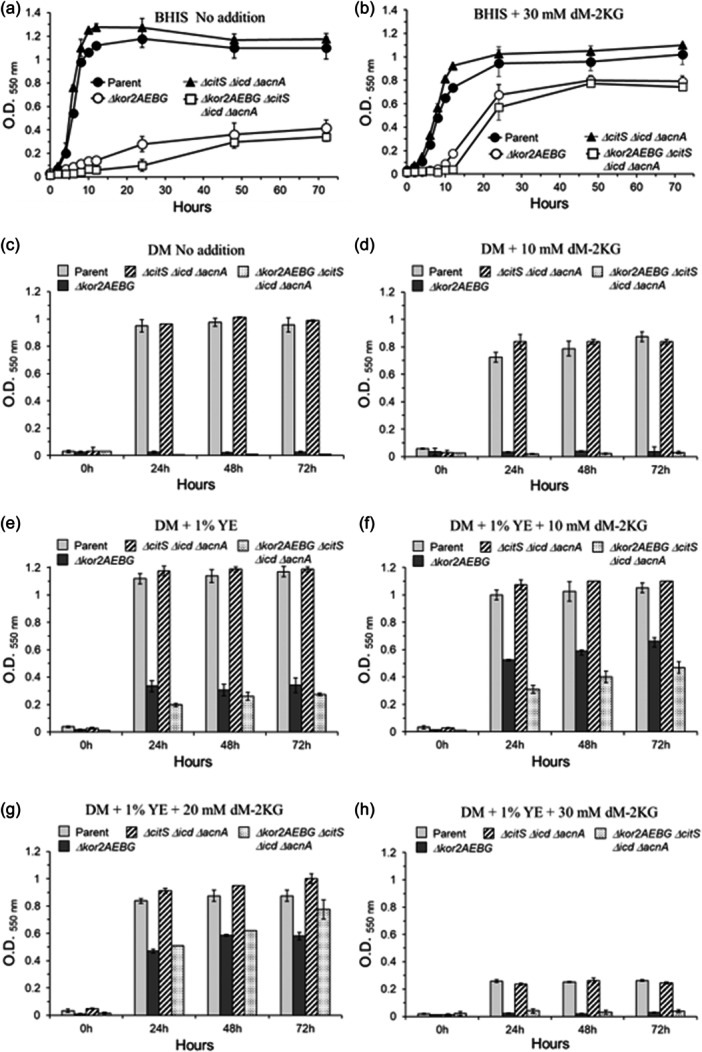
Growth of *B. fragilis* mutant strains in supplemented brain‐heart infusion (BHIS) media (Panels a and b), or chemically defined media with glucose (DM) (panels c to h). DM supplemented with 1% yeast extract (YE) (Panels e to h). Dimethyl‐2‐ketoglutarate (dM‐2KG) was added as indicated in the panels (Panels b–f). Strain designations are depicted for each panel. Media preparations and compositions are described in the Materials and Methods section.

Previous work with *B. thetaiotaomicron* has demonstrated that dM‐2KG, a membrane‐permeable precursor of 2‐KG is transported across the cytoplasmic membrane and is hydrolyzed to 2‐KG (Schofield et al., 2018). Therefore, we hypothesized that dM‐2KG could rescue the *Δkor2AEBG* phenotype. When 30 mM dM‐2KG was added to BHIS media it stimulated the growth of the *Δkor2AEBG* single mutant and the *Δkor2AEBG ΔcitS Δicd ΔacnA* quadruple mutant strains compared to no addition culture controls (Figure 12a,b). The effect of dM‐2KG from 1 mM to 40 mM on the growth of the *Δkor2AEBG* strain in BHIS media is shown in Supplemental File S9. In chemically defined media there was no growth stimulation of the *Δkor2AEBG* or the *Δkor2AEBG ΔcitS ΔacnA Δicd* quadruple mutant strain in the presence of 10 mM dM‐2KG compared to no addition control (Figure 12c,d), but in defined media containing 1% yeast extract, the addition of 10 mM or 20 mM dM‐2KG caused partial stimulation of growth of the *Δkor2AEBG* and the *Δkor2AEBG ΔcitS ΔacnA Δicd* quadruple mutant strains compared to basal media culture controls (Figure 12e, f, g). No growth defect was observed in the *ΔcitS ΔacnA Δicd* triple mutant strain compared to the parent strain. However, growth was strongly inhibited by 30 mM dM‐2KG in chemically defined media supplemented with yeast extract (Figure 12h). The addition of 10 mM dM‐2KG in defined media containing 1% tryptone did not restore growth of the *Δkor2AEBG* mutant strain compared to basal media culture controls (Supplemental File S10A,F). The addition of 30 mM dM‐2KG into the minimally defined media with or without 1% Tryptone was also highly inhibitory to growth (Supplemental File S10C,G). The effect of dM‐2KG on *B. fragilis* growth inhibition has not been defined. However, in *E. coli,* the addition of 20 mM dM‐2KG was required to cause a decrease in glucose uptake and growth inhibition (Doucette et al., 2011). Also, the addition of 1 mM dM‐2KG was required for *B. thetaiotaomicron* to increase levels of 2‐KG, l‐glutamate, and l‐glutamine (Schofield et al., 2018). The increase in 2‐KG levels in *B. thetaiotaomicron* halts the growth of growing cells in defined media, and it rescues the survival defect of a (p)ppGpp deficient strain (Schofield et al., 2018). Moreover, the membrane‐permeable ester dM‐2KG is cleaved by intracellular esterases to form 2‐KG in *E. coli* (Doucette et al., 2011); therefore, we cannot rule out transport deficiency, low “esterase” activity, or increase in 2‐KG might have led to growth arrest in *B. fragilis*.

Overall, our results suggest that Kor2AEBG might have dual function as determined by (1) growth deficiency in rich media is rescued by dM‐2KG supplementation suggesting a 2‐KG precursor by‐pass requirement for this enzyme, (2) an unknown metabolic function since the addition of l‐glutamate, l‐glutamine, or tryptone (a glutamate‐rich peptide source) did not compensate for the lack of the *kor2AEBG* genes in defined media suggesting that formation of 2‐KG as a precursor for the synthesis of l‐glutamate is not the only metabolic function of Kor2AEBG (Supplemental File S10I,J,K).

**Figure 13:**
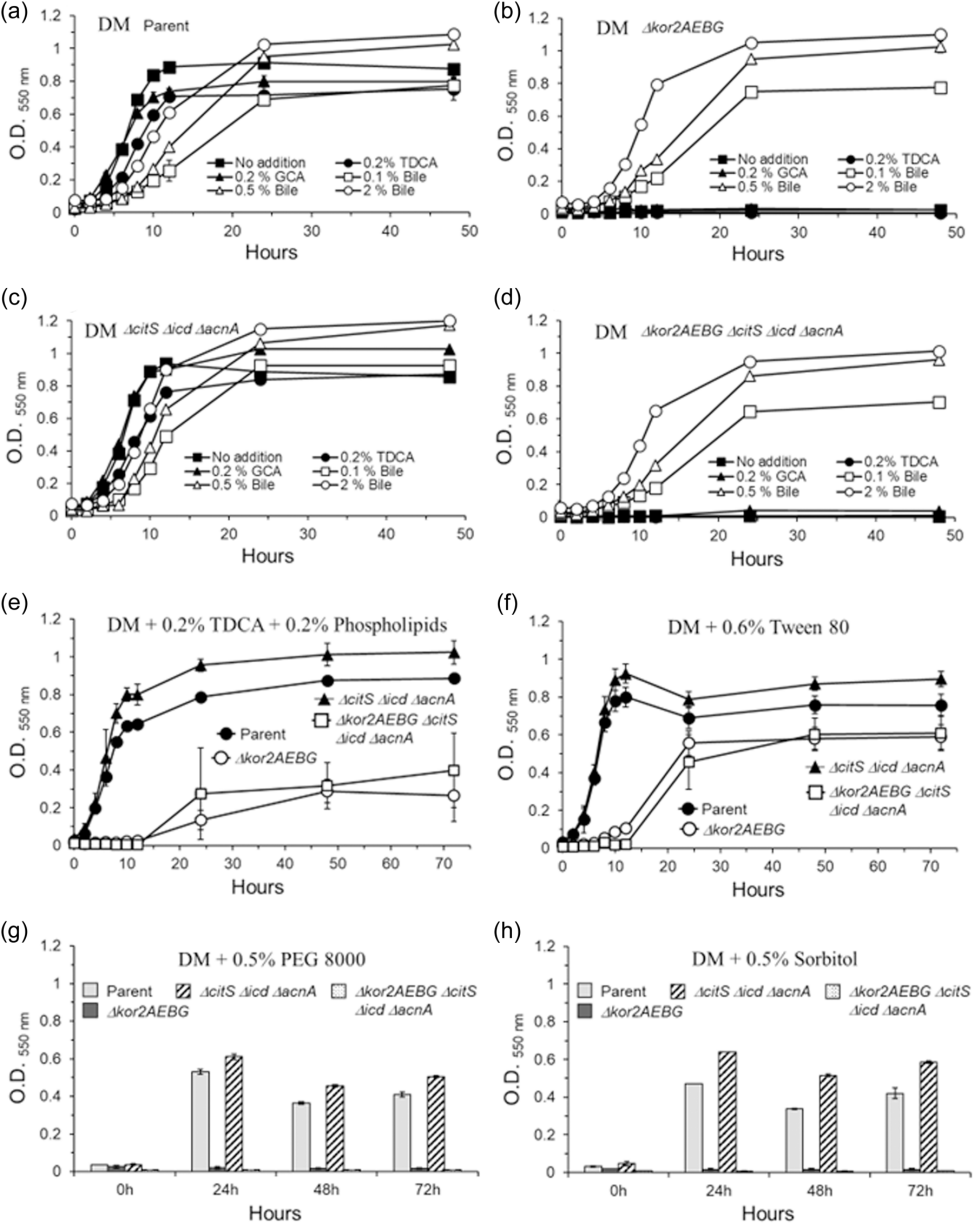
Growth of *B. fragilis* mutant strains in chemically defined media with glucose (DM). Panels a–d: DM with glucose was supplemented with 0.2% glycocholic acid (GCA), 0.2% taurodeoxycholic acid (TDCA), 0.1%, 0.5% or 2% ox‐bile (Bile), or no addition. (a) BF638R parent strain. (b) *Δkor2AEBG* single mutant strain. (c) *ΔcitS Δicd ΔacnA* triple mutant strain. (d) *Δkor2AEBG ΔcitS Δicd ΔacnA* quadruple mutant strain. Panels (e–h): The supplements added to DM are indicated in each panel. Phospholipids: Soy (*Glycine max*) phospholipids mixture, Sigma Aldrich Cat # 11145, Lot/Batch # BCCJ8356, contains roughly equal proportions of lecithin, cephalin, and phosphatidylinositol along with minor amounts of other phospholipids and polar lipids. It contains about 24% saturated fatty acids, 14% mono‐unsaturated and 62% poly‐unsaturated fatty acids. TDCA was mixed with phospholipids to solubilize micelles (Panel e). PEG: polyethylene glycol. Sorbitol was added instead of sorbitan (1,4‐sorbitol cyclic ester derivative of sorbitol dehydration), a component of Tween 80, which is a polyethoxylated sorbitan with one oleic acid as a primary fatty acid. Tween 80 was added to culture media as described in Holdeman et al., 1977. Strain designations are depicted in each panel.

To better understand how Kor2AEBG functions in *B. fragilis* metabolism, we supplemented chemically defined media with soluble cecal content material or bile extract to investigate if nutrients available in the intestinal tract could rescue *Δkor2AEBG* mutant strain growth. When chemically defined media was supplemented with 10% cell‐free sterile rat cecum aqueous content or with 2% ox bile, they strongly supported the growth of the *Δkor2AEBG* strain compared to growth in basal media (Supplemental File S10A,D,H). The growth of the *Δkor2AEBG* and *Δkor2AEBG ΔcitS Δicd ΔacnA* mutant strains in ox bile was restored in a dose‐dependent manner compared to the parent strain (Figure 13a,b,d). There was no significant growth defect of the *ΔcitS Δicd ΔacnA* triple mutant strain in defined media compared to the parent strain (Figure 13a,c). The addition of 0.2% taurodeoxycholic acid (TDCA) or 0.2% glycocholic acid (GCA) had no stimulatory growth effect on either *Δkor2AEBG* or *Δkor2AEBG ΔcitS Δicd ΔacnA* mutant strains compared to growth in the presence of ox bile (Figure 13b,d). This indicates that bile components other than bile salts were involved in the growth stimulation of the mutant strains lacking *kor2AEBG* genes.

### 3.13 The addition of phospholipids or tween 80 restores the growth of *Δkor2AEBG* mutant in chemically defined media

**Figure 14:**
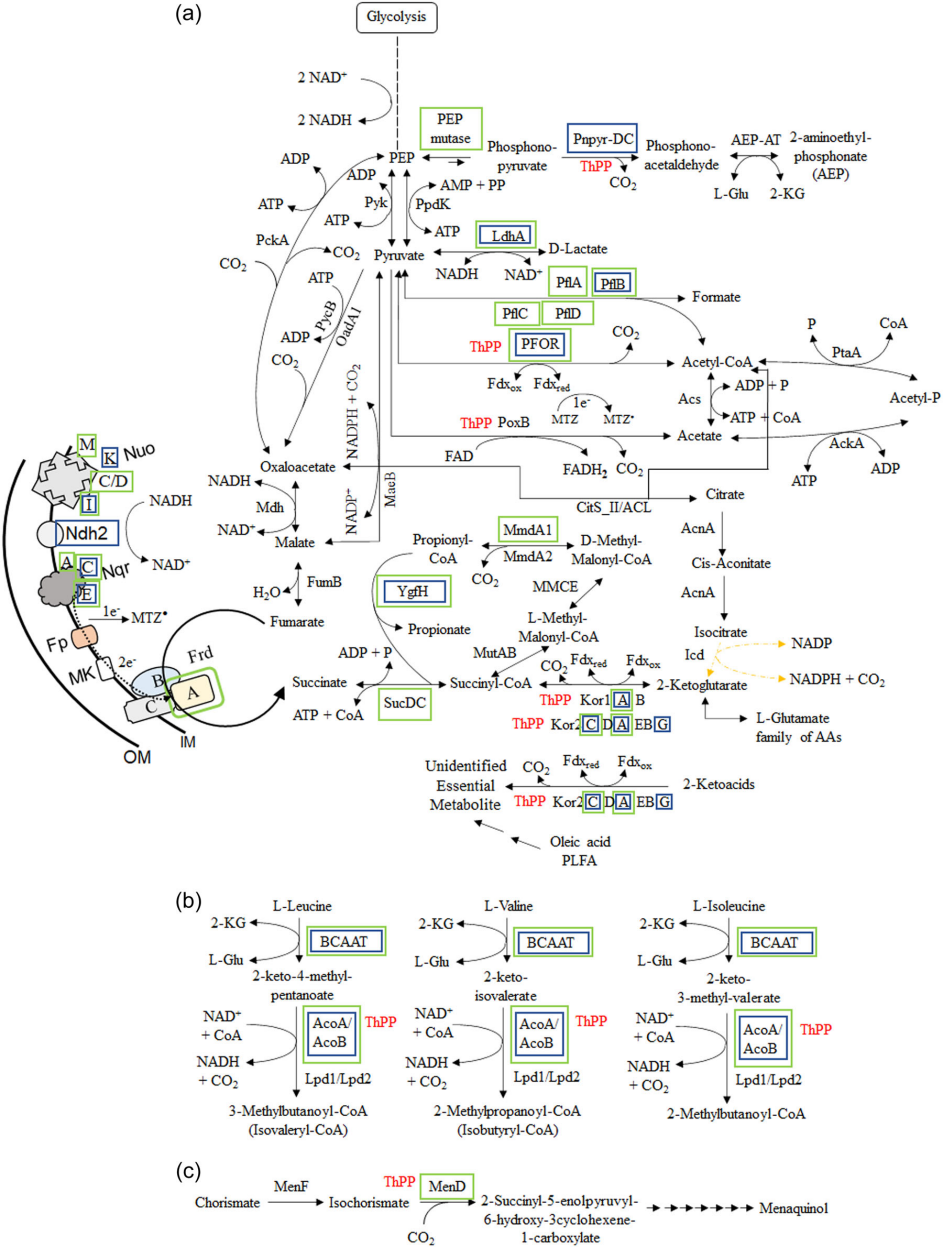
Schematic diagram of *B. fragilis* 638R (a) central metabolism, (b) degradation of branched amino acids pathway, and (c) initial steps of menaquinol biosynthetic pathway. The thiamine diphosphate binding enzymes are depicted with ThPP in red font beside the enzyme designation. The enzyme subunits with predicted stable protein‐protein interaction with Pir1 are highlighted with a blue box and interaction with Pir2 with a green box. The compilation of the pathway reactions was based on public database https://www.genome.jp/pathway/bfg00020,/pathway/bfg00010,/pathway/bfg00620,/pathway/bfg00640,/pathway/bfg00280,/pathway/bfg00130), *B. fragilis* 638R genome (GenBank accession number FQ312004), and Refs.: Allison & Robinson, 1970; Allison et al., 1979; Butler et al., 2023; Harris & Reddy, 1977; Ito et al., 2020; Macy et al., 1978; Rios‐Covian et al., 2015; Schofield et al., 2018; Sund et al., 2008;. Zhang et al., 2003. Orange dash/dot arrows indicate there is no report that 2‐ketoglutrate or l‐glutamate is formed from isocitrate dehydrogenase (Icd) pathway under anaerobic conditions (Allison & Robinson, 1970; Allison et al., 1979; Schofield et al., 2018). AAs, amino acids; AckA, acetate kinase (BF638R_0490); AcnA, aconitase (BF638R_3569); AcoA/AcoB, 2‐Ketoisovalerate dehydrogenase, a single peptide containing alpha‐ and beta‐subunit domains (BF638R_1637); Acs, acetate,CoA ligase (BF638R_4449); AEP‐AT, 2‐aminoethylphosphonate aminotransferase (BF638R_1869) and (BF638R_3511); BCAAT, branched‐chain amino acid transferase (BF638R_3846); CitS_II/ACL, citrate synthase/2‐methylcitrate synthase/ACL family (BF638R_3567); Fp, flavoprotein; Frd, fumarate reductase, FrdCAB (BF638R_4499‐4501); FumB, fumarate hydratase class I, anaerobic (BF638R_0646); Icd, isocitrate dehydrogenase (BF638R_3568); IM, inner cytoplasmic membrane; Kor1AB, 2‐Ketoglutarate ferredoxin oxidoreductase (BF638R_4321‐4322); Kor2CDAEBG, 2‐Ketoglutarate ferredoxin oxidoreductase (BF638R_1660‐1665); LdhA, d‐Lactate dehydrogenase (BF638R_1473); Lpd1, dihydrolipoamide dehydrogenase‐E3 (BF638R_0023); Lpd2, dihydrolipoamide dehydrogenase‐E3 (BF638R_1634); MaeB, malate dehydrogenase [oxaloacetate‐decarboxylating NADP^+^‐dependent] (BF638R_3435); Mdh, malate dehydrogenase (BF638R_0537); MenD, 2‐Succinyl‐5‐enolpyruvyl‐6‐hydroxy‐3‐cyclohexene‐1‐carboxylic‐acid synthase (BF638R_1316); MenF, isochorismate synthase (BF638R_1315); MK, menaquinone; MMCE, methylmalonyl‐CoA epimerase (BF638R_3151); MmdA1 (PccB), propionyl‐CoA carboxylase beta‐chain (BF638R_1625); MmdA2 (PccB), propionyl‐CoA carboxylase beta‐chain (BF638R_3367); MutAB, methylmalonyl‐CoA mutase, small (A) and large (B) subunits (BF638R_3616‐3615); Nad2, NADH dehydrogenase, FAD‐containing subunit (BF638R_1612); Nqr, Na+‐translocating NADH‐quinone oxidoreductase, NqrABCDE, (BF638R_2136_2140); Nuo, NADH,ubiquinone oxidoreductase, NuoABC/DHIJKLMN (BF638R_0850‐0841); OadA1, pyruvate/oxaloacetate carboxyltransferase (BF638R_2828); OM, outer membrane; PckA, phosphoenolpyruvate carboxykinase (BF638R_4326); PEP mutase, phosphoenolpyruvate mutase (BF638R_1867); PFLA, phospholipid fatty acids; PflA, pyruvate formate‐lyase 1, activating enzyme (BF638R_1338); PflB, pyruvate formate‐lyase 1 [formate acetyltransferase 1] (BF638R_1339); PflC, pyruvate formate‐lyase 2, activating enzyme (BF638R_4262); PflD, pyruvate formate‐lyase 2 [formate acetyltransferase 2] 9BF638R_4263); PFOR, pyruvate ferredoxin oxidoreductase (BF638R_3194); Pnpyr‐DC, phosphonopyruvate decarboxylase (BF638R_1868); PoxB, an inner membrane enzyme that catalyzes oxidative decarboxylation of pyruvate to form acetate + CO_2_ (BF638R_3245); PpdK, pyruvate orthophosphate dikinase (BF638R_2565); PtaA, phosphate acetyltransferase (BF638R_0489); PycB, pyruvate carboxylase biotin‐containing subunit (BF638R_1927); Pyk, pyruvate kinase (BF638R_4359); SucDC, succinyl‐CoA synthase alpha‐ and beta‐chains (BF638R_2360‐2361); YgfH, succinate‐CoA transferase subfamily (BF638R_0025).

To test if other components of bile such as phospholipids/fatty acids (Alvaro et al., 1986, Boyer, 2013) could restore the growth of the *Δkor2AEBG* mutant, we used a commercially available phospholipids mixture from soy (Sigma Aldrich Cat # 11145) containing roughly equal proportions of lecithin, cephalin, and phosphatidylinositol along with minor amounts of other phospholipids and polar lipids, and a source of saturated, monounsaturated and polyunsaturated fatty acids as certified by the supplier (Lot/Batch number BCCJ8356). When these mutant strains were grown in defined media containing 0.2% of a soy phospholipids mixture (in the presence of TDCA to solubilize micelles), it caused a partial growth stimulation of the *Δkor2AEBG* and *Δkor2AEBG ΔcitS Δicd ΔacnA* mutant strains (Figure 13e) compared to no addition cultures (Figure 13b,d). When the surfactant and emulsifier tween 80 [Polyoxyethylene (80) sorbitan monooleate]–a media supplement to enhance the growth of some anaerobic bacteria (Holdeman et al., 1977)–which contains oleic acid, a monounsaturated fatty acid present in phosphatidylcholine, the major phospholipid in bile (Boyer, 2013), was added to the media, it caused the *Δkor2AEBG* and *Δkor2AEBG ΔcitS Δicd ΔacnA* mutant strains to grow to levels comparable to the parent strain though with an extended lag‐growth phase (Figure 13f). The growth rate of the *Δkor2AEBG* and *Δkor2AEBG ΔcitS Δicd ΔacnA* mutant strains in defined media containing tween 80 was similar to growth rate observed in BHIS containing dM‐2KG as shown above (Figure 12b). The addition of PEG 8000 or sorbitol (used instead of sorbitan, a cyclized ester of sorbitol in tween 80) did not affect the growth of the *Δkor2AEBG* nor *Δkor2AEBG ΔcitS Δicd ΔacnA* mutant strain (Figure 13g,h). This indicates that it was the oleic acid component of tween 80 responsible for promoting growth stimulation. Taken together, these findings indicate that the ThPP‐binding Kor2CDAEBG enzyme complex has a novel metabolic function essential for *B. fragilis* growth that remains to be characterized. Collectively, our investigation reveals new information on *B. fragilis* central metabolism and its modulatory control by protein‐protein interactions (Figure 14).

## 4 DISCUSSION

In this study, we show that Pir1 and Pir2 proteins play in part a role in modulating anaerobic fermentation pathways as demonstrated by changes in the SCFAs, mostly minor SCFAs products in different growth conditions and alterations in the susceptibility to the antimicrobials MTZ and AMIX. It remains to be defined if changes in metabolism correlate with protein‐protein interactions of Pir1 or Pir2 with PFOR and/or Zn‐ADH as demonstrated by bacterial THS assays and potentially with several other enzymes acting in the central carbon metabolism and energy preservation pathways as predicted by computational structural models (Supplemental File S5). A schematic description of the metabolic enzymes of the central metabolism and energy‐generating processes, degradation of branched amino acids (BCAA), and synthesis of menadione that form protein‐protein interaction with Pir1, Pir2 or both Pir1 and Pir2 is shown in Figure 14. These findings agree with reports demonstrating a role for pirin in the control of TCA cycle switching to fermentation metabolism or aerobic respiration in aerobic and facultatively anaerobic bacteria (Soo et al., 2007; Hansen et al., 2012; Tala et al., 2018; Young et al., 2023). We assume that the interactions of Pir1 and Pir2 with BCAAT and AcoA/AcoB, in the degradation pathway of branched‐chain amino acids, are linked to modulating upregulation and downregulation production of minor SCFA such as isobutyrate, isovalerate, though experimental evidence that enzymatic activities are indeed altered by interactions with pirins remain to be determined.

Moreover, pirins undergo Fe(II) to Fe(III) redox changes during oxygen exposure or iron‐limiting conditions to modulate protein‐protein interactions and conformational dynamics (Ahsan et al., 2023); Barman & Hamelberg, 2016; Liu et al., 2013). It is in the pirin active Fe(III)‐form, but not in the inactive Fe(II)‐form that human pirin coordinates binding to and regulates the nuclear factor NF‐κB function (Liu et al., 2013). In this regard, we cannot rule out that Pir1 and Pir2, in the Fe(III) state, form stable protein‐protein interactions with *Bacteroides* proteins such as the *Bacteroides* aerotolerance protein BatC (Tang et al., 1999), and with oxygen‐sensitive enzymes such as PFOR, Pfl, Frd, aconitase, fumarase, and NADH dehydrogenases (Khademian & Imlay, 2020; Lu & Imlay, 2019; Lu & Imlay, 2021; Pan & Imlay, 2001) might also play a role in sensing redox functional groups such as iron‐sulfur clusters during aerotolerance when expression of *pir1* and *pir2* genes are upregulated by oxygen and iron limiting conditions (Figure 14 and Supplemental File S5A). If this is true, it is possible that pirin interactions affect enzyme kinetics and may help to explain variations in the production of minor SCFAs in different anaerobic culture growth phases, during oxygen exposure, or iron‐limiting conditions. Furthermore, the effects of pirins on the susceptibility of MTZ and AMIX were statistically significant in cultures exposed to oxygen but had minimal effect in anaerobic cultures. However, the modulatory effect of pirins on oxidoreductases and ThPP‐binding 2‐ketoacids:ferredoxin oxidoreductase activities remain to be defined. Very little is known about anaerobic bacteria control of reductive pathways. Perhaps, in an anaerobic redox milieu inside intact cells, protein‐protein interactions of oxidoreductases with redox‐sensitive regulatory modulators such as pirin proteins might assist in exerting more stringent controls in regulating substrate access, the direction of catalysis, and the rate of the electron transfer traveling through redox enzyme complex pathway in vivo (Agapakis & Silver, 2010; McMillan et al., 2013). This is stated here because the reduction of the redox cycling agents paraquat or benzyl viologen are widely used as artificial electron acceptors to determine PFOR activity in in vitro assays (Noth et al., 2013; Menon & Ragsdale, 1997; Khademian & Imlay, 2020; Bock et al., 1996; Williams et al., 1987; Thorgersen et al., 2022). However, there is little evidence that paraquat or benzyl viologen is reduced inside intact metabolic active *B. fragilis* cells, at least not to a detectable toxic level in the presence or absence of oxygen (Rocha et al., 1996; Rocha and Smith, 1997, Figure 8, Supplemental File 8). Previous studies showed that paraquat did not cause significant physiological or gene expression changes in *B. fragilis* growing in an anaerobic culture, or during oxygen exposure compared to control cultures (Rocha et al., 1996; Rocha & Smith, 1997).

Incidentally, constitutive expression of Pir1 or Pir2 significantly increased BF638R parent strain sensitivity to MTZ to similar levels of sensitivity found in the *ΔfrdB* mutant following oxygen exposure. Here we show that in the mutant lacking fumarate reductase subunit B activity, *ΔfrdB*, a backup/retention of NADH dehydrogenase coupled redox pair cycling electron transfer is assumed to occur. This is supported by our findings showing that inhibitors of the ETS such as acriflavine, which inhibits NADH dehydrogenase reduction of a flavoprotein, and HQNO inhibits electron flow of reduced quinone as an electron donor for the reduction of fumarate to succinate by fumarate reductase (Harris & Reddy, 1977), increase MTZ sensitivity. Support for an NADH backup/retention occurrence is the fact that the level of MTZ susceptibility of the *ΔfrdB* deletion mutant strain was not further altered in the presence of ETS inhibitors compared to the parent strain (Figure 7a,b). These findings support recent studies showing that in heme‐limiting growth conditions, *B. fragilis* becomes hypersensitive to MTZ and abolishes high levels of MTZ resistance (Paunkov et al., 2022a). Since fumarate reductase activity is heme‐dependent (Macy et al., 1975; Baughn and Malamy, 2003), the effect of heme availability on MTZ susceptibility seems to correlate nicely with the fumarate reductase mutants *ΔfrdB* and *ΔfrdC* susceptibility to MTZ. Moreover, whether the predicted stable protein‐protein interactions of Pir2 with fumarate reductase FrdA subunit, and the interactions of Pir1 and Pir2 with subunits of three NADH dehydrogenase types encoded in *B. fragilis* 638R genome; NADH:ubiquinone oxidoreductase (Nuo), NADH:quinone oxidoreductase (Nqr), and the NADH FAD‐containing dehydrogenase II (Ndh2) (Butler et al., 2023; Ito et al., 2020) play a role in the modulation of NADH dehydrogenases redox activities remains to be defined (Figure 14 and Supplemental File S5A). Taken together, it is likely that dysregulation of the cellular membrane bioenergetics redox balance could account for an increase in the reduction of MTZ.

Support for the fact that disruption of bioenergetics processes affects MTZ susceptibility comes from the findings showing that analogs of menaquinone precursor, HNQ and NQ, abolished sensitivity to MTZ either by blocking reductive activation or by protecting against radical toxicity. Energy conservation mechanisms in commensal and pathogenic intestinal anaerobes are not well understood and among *Bacteroides* themselves there seem to be significant differences in bioenergetics which could be explored for novel antimicrobials. For example, the susceptibility to the antiparasitic and antibacterial agent closantel, a hydrogen ionophore that decouples oxidative phosphorylation and leads to inhibition of ATP synthesis (Gooyit & Janda, 2016; Hlasta et al., 1998; Rajamuthiah et al., 2014; Skuce & Fairweather, 1990; Stephenson et al., 2000; Tran et al., 2016) varied immensely when comparing *B. fragilis, B. thetaiotaomicron, and B. vulgatus* (Supplemental file S11). This also seems to be the condition among different anaerobic bacteria such as *Porphyromonas gingivalis*, *Prevotella melaninogenica*, and *Clostridioides difficile* as their susceptibility to MTZ in the presence of HNQ varies greatly compared to *B. fragilis* (Supplemental File S12).

Moreover, we show in this study that two KFORs, the *ΔPFOR::tetQ,* associated with the oxidative TCA branch and *Δkor2AEBG*, associated with the reductive TCA branch, showed moderate effect in MTZ resistance. However, it was the additional deletion of the *citS icd acnA* genes with *ΔPFOR::tetQ Δkor2ABG* background in the Δ*PFOR::tetQ Δkor2ABG ΔcitS Δicd ΔacnA* quintuple mutant strain that caused an eightfold increase in resistance to MTZ MIC (4 μg/mL) compared to the parent strain. It is not clear what role deletion of the *citS icd acnA* genes plays in enhancing MTZ resistance. The *ΔcitS Δicd ΔacnA* triple mutant alone had a twofold MIC increase in MTZ (1 μg/mL) and AMIX (4 μg/mL) resistance compared to the parent strain. Interestingly, no synthesis of 2‐KG or l‐glutamate has been demonstrated to occur via the citrate‐isocitrate pathway in *Bacteroides* (Allison & Robinson, 1970; Allison et al., 1979; Schofield et al., 2018) nor does it compensate for the lack of Kor2AEBG (this study). Although the regulation and functionality of the *citS icd acnA* operon in *B. fragilis* remains to be further explored, the lack of CitS Icd AcnA may have altered other unidentified metabolic activities to maintain the redox carbon balance flow and energy generation that somehow enhances MTZ resistance.

Another aspect of *B. fragilis* metabolism revealed in this study was the role of KFOR orthologs Kor1AB and Kor2CDAEBG putative protein complexes. In the KEGG pathway database, the function of *kor1AB* and *kor2ABG* genes are indicated to perform reductive synthesis of 2‐KG via carboxylation of succinyl‐CoA (https://www.genome.jp/pathway/bfg00020 and https://www.genome.jp/pathway/bfg00620). This agrees with the fact that the *kor1AB* and *kor2ABG* gene orthologs in several microorganisms have been shown to synthesize 2‐KG from succinyl‐CoA (Chen et al., 2019; Dörner & Boll, 2002; Mai & Adams, 1996; Yamamoto et al., 2006; Yamamoto et al., 2010). However, our findings indicate that Kor2AEBG may have an uncharacterized metabolic activity other than a reductive synthesis of 2‐KG since the addition of l‐glutamate, l‐glutamine, or tryptone did not restore the growth of the *Δkor2AEBG* mutant.

In this regard, we showed in this study that rat cecum content or ox bile strongly restored growth deficiency of the *Δkor2AEBG* deletion strain in defined media. We ruled out that bile salts were involved and showed that the addition of a phospholipids mixture or oleic acid is sufficient for restoring the growth of the *Δkor2AEBG* deletion strain. It remains unclear which metabolic pathway is used by *B. fragilis* to stimulate growth in the presence of oleic acid in the absence of *kor2ADBG* genes; however, we presume that oleic acid or other fatty acids might be a contributing factor present in bile which are known to stimulate growth of several bile‐resistant anaerobic bacteria (Holdeman et al., 1977). We do not think that the production of acetyl‐CoA from the fatty acid β‐oxidation degradation is involved in compensating for the lack of Kor2AEBG since other pathways for acetyl‐CoA production from decarboxylation of pyruvate such as PFOR or Pfl remain intact, and lack of the citrate/isocitrate pathway does not compensate for Kor2AEBG deficiency. Studies have shown that degradation of fatty acids in microorganisms occurs via coordinated alternations between α‐oxidation, β‐oxidation, ω‐oxidation [(ω1), (ω2), (ω3), (ω4)‐type oxidation] and fatty acid hydroxylation by fatty acid hydratases mechanisms which can produce shorter dicarboxylic acids, shorter hydroxy‐fatty acids, keto‐fatty acids, and short branched‐dicarboxylic acids (from long branched‐fatty acids) (Child et al., 2019; Gatter et al., 2014; Miura & Fulco, 1974; Hagedoom et al., 2021; Kang et al., 2017a; Kang et al., 2017b; Miura & Fulco, 1975; Miura, 2013; Ruettinger et al., 1974; Shoun et al., 1985; Vanhanen et al., 2000). However, the product(s) of 2‐ketoacid oxidoreductase metabolism from Kor2AEBG activities that intersect with oleic acid or other long fatty acids catabolism remains to be defined (Figure 14).

In conclusion, we show that the central metabolism and bioenergetics of *B. fragilis* still have many features that are yet to be explored and are potential targets for the development of novel narrow‐spectrum antibiotics. One of these targets is the ThPP‐binding enzymes related to anaerobic bacteria metabolism to which AMIX was shown to be effective against *B. fragilis in vivo*. Although intraperitoneal administration of AMIX significantly decreased CFU counts, compared to elimination of *B. fragilis* with intra‐cage administration, this difference may have been due to diminished systemic antimicrobial diffusion through the encapsulated barrier of the artificial tissue cage. Future studies using different experimental abscess models would clarify this matter. Nonetheless, our findings agree with previous studies showing the effectiveness of AMIX against infection caused by *C. difficile*, *H. pylori*, *C. jejuni*, and by oral anaerobic pathogens in infections such as gingivitis, periodontitis, and in biofilms in *vivo* animal models (Gui et al., 2019; Gui et al., 2020; Hoffman et al., 2014; Hoffman, 2020; Hutcherson et al., 2017; Kennedy et al., 2016; Reed et al., 2018; Warren et al., 2012). Taken together, these findings show that AMIX is a potential antimicrobial for *B. fragilis* extra‐intestinal infection. Lastly, we show evidence that the α*‐*ketoglutarate ferredoxin oxidoreductase Kor2CDADBG is an essential enzyme for *B. fragilis* growth and plays a novel function in anaerobic central metabolism which remains to be completely characterized. We believe that understanding the *B. fragilis* central carbon metabolism and energy‐conservation pathways may lead to the development of novel narrow‐spectrum selective antimicrobials for the inhibition of essential metabolic targets (Baek et al., 2011; Bunik et al., 2013; Cook et al., 2014; Feng et al., 2015; Gil‐Gil et al., 2020; Murima et al., 2014; Stokes et al., 2019).

## AUTHOR CONTRIBUTIONS

**Andrea M. Gough**: Methodology; investigation; validation; visualization; formal analysis. **Anita C. Parker**: Methodology; investigation; validation; visualization; formal analysis. **Patricia J. O'Bryan**: Methodology; validation; investigation; formal analysis. **Terence R. Whitehead**: Methodology; validation; investigation. **Sourav Roy**: Writing–review and editing; methodology; validation; visualization; investigation; formal analysis; software. **Brandon L. Garcia**: Validation; writing–review and editing; methodology; software; visualization; formal analysis; investigation. **Paul S. Hoffman**: Writing–review and editing; conceptualization; formal analysis. **C. Jeffrey Smith**: Formal analysis; writing–review and editing; conceptualization. **Edson R. Rocha**: Writing–original draft; investigation; validation; writing–review and editing; project administration; supervision; conceptualization; methodology; formal analysis; funding acquisition; visualization.

## CONFLICT OF INTEREST STATEMENT

None declared.

## ETHICS STATEMENT

All procedures involving animals followed the guidelines given by the National Research Council's *Guide for the Care and Use of Laboratory Animals* (National Research Council, 2011) and approved by the Institutional Animal Care and Use Committee of East Carolina University.

## Supporting information

Supporting information.

Supporting information.

## Data Availability

The data that supports the findings of this work are available at https://www.ncbi.nlm.nih.gov/gds/, GEO Datasets GSE241210 and GSE241676.
