# Supplementary material for: New functions of pirin proteins and a 2‐ketoglutarate: Ferredoxin oxidoreductase ortholog in Bacteroides fragilis metabolism and their impact on antimicrobial susceptibility to metronidazole and amixicile
Source: Microbiologyopen. 2024 Aug 7;13(4):e1429. doi: 10.1002/mbo3.1429 (PMC11304471; doi:10.1002/mbo3.1429)
Supplement: Supplementary file 2 — Supporting information. [file MBO3-13-e1429-s002.pdf]

Supplemental File S1

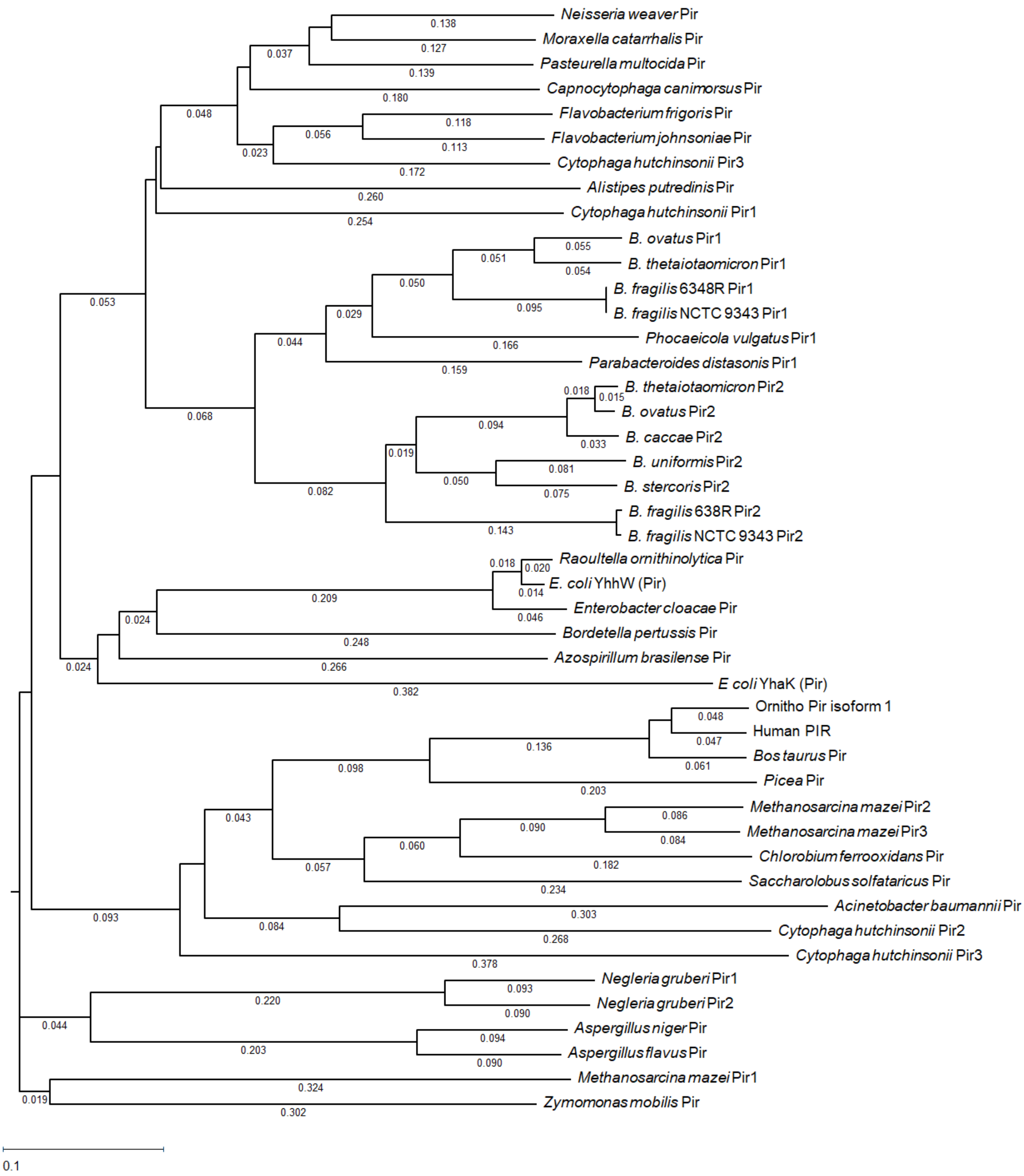

Supplemental File S1. Phylogenetic relationship of 49 Pirin-like protein homologues representatives from Bacteroidetes, and from other prokaryotes, eukaryotes, and Archaea. The unrooted phylogenetic tree was constructed from multiple amino acid sequences alignment based on ClustalW algorithm in MegAlign Pro program of Lasergene Version 17.5.0 (DNASTAR, Inc). The built-in Neighbor Joining method was used to

calculate the distances between all pairs and the calculated relative distance values are depicted in parenthesis following each tree entry name. GenBank accession numbers and abbreviations are as follow: Acinetobacter baumanii AC12 Pir (AHX26982). *Alistipes putredinis* DSM 17216 Pir (EDS02825). *Aspergillus flavus* AF70 Pir (KOC10185). *Aspergillus niger* ATCC 1015 Pir (EHA28466). *Azospirillum brasilense* Pir (TWA73044). *Bacteroides fragilis* 638R Pir1 (CBW23516), Pir2 (CBW22007). *Bacteroides fragilis* NCTC 9343 Pir1 (CAH08727), Pir2 (CAH07099). *Bacteroides ovatus* ATCC 8483 Pir1 (EDO13012), Pir2 (EDO09940). *Bacteroides stercoris* ATCC 43183 Pir2 (EDS16761). *Bacteroides thetaiotaomicron* VPI-5482 Pir1 (AAO75294), Pir2 (AAO76683). *Bacteroides uniformis* ATCC 8492 Pir2 (EDO51902). *Bordetella pertussis* CS Pir (AEE66951). *Bos taurus* Pir (NC_037357). *Capnocytophaga canimorsus* Cc5 Pir (AEK22609). *Chlorobium ferrooxidans* DSM 1303 Pir (EAT59973). *Cytophaga hutchinsonii* ATCC 33406 Pir1 (ABG57630), Pir2 (ABG58934), Pir3 (ABG59359), and Pir4 (ABG60251). *Enterobacter cloacae* subsp. cloacae ATCC 13047 Pir (ADF64329). *Escherichia coli* str K12 MG1655 YahK (NP_417577), YhhW (NC_000913). *Flavobacterium frigoris* PS1 Pir (EIA09520). *Flavobacterium johnsoniae* UW101 Pir (ABQ04767). Human Pir: *Homo sapiens* (NP_003653). *Methanobactins mazei* Go1 Pir1 (AAM32493), Pir2 (AAM32593) and Pir3 (AAM32603). *Moraxella catarrhalis* 7169 Pir (EGE09966). *Naegleria gruberi* Pir1 (EFC43129), Pir2 (EFC40196). *Neisseria weaveri* ATCC 51223 Pir (EGV38178). Ornitho Pir Isoform 1: *Ornithorhynchus anatinus* pirin isoform X1 (XP_003431123). *Parabacteroides distasonis* ATCC 8503 Pir1 (ABR41886). *Pasteurella multocida* 36950 Pir (AET16501). *Phocaeicola vulgatus* ATCC 8482 Pir1 (ABR40270). Picea: *Picea sitchensis* Pir (ABK27098). *Raoultella ornithinolytica* 10-5246 Pir (EHT04992). *Saccharolobus solfataricus* Pir (AKA74406). *Zymomonas mobilis* subsp. mobilis ZM4 Pir (AAV89961).

Supplemental File S2

```
                          1 ........10........20........30........40........50........60........70........80
E. coli YhaK              1 -MITTRTARQCGQADYGWLQARYTFSFGHYFDPKLLGYASLRVLNQEVLAPGAAFQPRTYPKVDILNVILDG-EAEYRDS
BF638R_1469 Pir2          1 MKKVVHRSDTRGRSVYDWLDSHHSFSFDEYYNPERVHFGALRVLNDDRVAPGEGFQPHPHKNMEIVSIPLKG-LLAHGDS
BF638R_3039 Pir1          1 MKTVVDKASSRGYFNHGWLKTHHTFSFANYYNPSRMHFGVLRVLNDDSVDPEMGFDTHPHQNMEVISIPLKG-YLRHGDS
BT_0187 Pir1              1 MKKVIDRASSRGYFNHGWLKTHHTFSFANYYNPERIHFGALRVLNDDSVDPSMGFDTHPHKNMEVISIPLKG-YLRHGDS
BT_1576 Pir2              1 MKKVIHKADTRGHSQYDWLDSYHTFSFDEYFDSDRINFGALRVLNDDKVAPGEGFQTHPHKNMEIISIPLKG-HLQHGDS
E. coli YhhW              1 -MIYLRKANERGHANHGWLDSWHTFSFANYYDPNFMGFSALRVINDDVIEAGQGFGTHPHKDMEILTYVLEG-TVEHQDS
Human_Pir                 1 --MGSSKKVTLSVLSREQSEGVGARVRRSIGRPELKNLDPFLLFDEFKGGRPGGFPDHPHRGFETVSYLLEGGSMAHEDF
                                           R        R        E K                     * *
BF_Pir1 binding sites                     NHG         FAN Y P  M     RV          G DTHPHQNM
BF_Pir2 binding sites                                 SFDEYY P  V     R            FQPHPHKNM
                         81 ........90.......100.......110.......120.......130.......140.......150.......160
E. coli YhaK             79 EGNHVQASAGEALLLSTQPGVSYSEHNLSKDKPLTRMQLWLDAC--PQRENPLIQKLALNMGK-QQLIASPEGAMG--SL
BF638R_1469 Pir2         80 KKNSRTITVGDIQVMSAGTGIYHSEMNGSKSEPVEFLQIWIIPK--ERNTHPLYQDYDIRGLLKKDELAFILSPNGSTPA
BF638R_3039 Pir1         80 VKNTRTITPGDIQVMSTGKGIFHSEYNGSDKEQLEFLQIWVFPR--IENTEPEYNNYDIRPLLKRNELALIISPDGKVPA
BT_0187 Pir1             80 VQNTKTITPGDIQVMSTGSGIYHSEYNDSKEEQLEFLQIWVFPR--IENTKPEYNNFDIRPLLKPNELSLFISPNGKTPA
BT_1576 Pir2             80 KKNSRIITVGEIQTMSAGTGIFHSEVNASPVEPVEFLQIWIMPR--ERNTHPVYKDFSIKELERPNELAVIVSPDGSTPA
E. coli YhhW             79 MGNKEQVPAGEFQIMSAGTGIRHSEYNPNSTERLHLYQIWIMPE--ENGITPRYEQRRFDAVQGKQLVLSPDARDG--SL
Human_Pir                79 CGHTGKMNPGDLQWMTAGRGILHAEMPCSE-EPAHGLQLWVNLRSSEKMVEPQYQELKSEEIPKPSKDGVTVAVISGEAL
                                                  * *

BF_Pir1 binding sites                            F                 W F
BF_Pir2 binding sites                         T  Y                   I     R
                        161 .......170.......180.......190.......200.......210.......220.......230.......240
E. coli YhaK            154 QLRQQVWLHHIVLDKGESANF-----QLHGPR--AYLQSIHGKFHALTH-HEEKAALTCGDGAFIRDEANITLVADSPLR
BF638R_1469 Pir2        158 KLLQDTWFSMGEIGAGKTVEY-----TLHGTDMGVYVFLIEGEVKIDD------VILTRRDGLGISEIENFEIETLKDSK
BF638R_3039 Pir1        158 SIKQDAWFSMGTFDAGKSFEY-----KLHQEGNGVYLFIIEGDVEVAG------NRLSRRDGIGLWDTKSFKVEITQEAT
BT_0187 Pir1            158 SIKQDAWFSMGDFDTERTIEY-----CMHQEGNGAYLFVIEGEISVAD------EHLAKRDGIGIWDTKSFSIRATKGTK
BT_1576 Pir2            158 SLLQDTWFSIGKVEAGKKLGY-----HLHQSHGGVYIFLIEGEIVVDG------EVLKRRDGMGVYDTKSFELETLKDSH
E. coli YhhW            155 KVPQDMELYRWALLKDEQSVH-----QIAAERR-VWIQVVKGNVTIN------GVKASTSDGLAIWDEQAISIHADSDSE
Human_Pir               158 GIKSKVYTRTPTLYLDFKLDPGAKHSQPIPKGWTSFIYTISGDVYIGPDDAQQKIEPHHTAVLGEGDSVQVENKDPKRSH


                        241 .......250.......260.......270.......280.......290...
E. coli YhaK            226 ALLIDLPV---------------------------------------------
BF638R_1469 Pir2        227 ILLIEVPM---------------------------------------------
BF638R_3039 Pir1        227 LLLMEVPMR--------------------------------------------
BT_0187 Pir1            227 LLVMEVPM---------------------------------------------
BT_1576 Pir2            227 ILLIEVPM---------------------------------------------
E. coli YhhW            223 VLLFDLPPV--------------------------------------------
Human_Pir               238 FVLIAGEPLREPVIQHGPFVMNTNEEISQAILDFRNAKNGFERAKTWKSKIGN
```

Supplemental File S2. Multiple alignment of predicted pirin (Pir) proteins from *Bacteroides fragilis* 636R (BF638R) Pir1 (CBW23516) and Pir2 (CBW22007), *Bacteroides thetaiotaomicron* VPI 5482 (BT), Pir1 (AAO75294) and Pir2 (AAO76683) with pirin proteins from *Escherichia coli* str. K-12 substr. MG1655 YhaK (NP_417577) and YhhW (NP_417896), and Human Pir (NP_003653). The conserved amino acid residues (>50 %) are labeled with black boxes. Semiconserved amino acid substitutions are depicted by grey boxes. Alignment of the protein sequences was performed using ClustalW algorithm from the MegAlign Pro component of the DNASTAR Lasergene software Version: 17.5.0 (DNASTAR, Inc. Madison, WI). The N-terminal domain residues His56, His58, His101, and Glu103 ligands of the iron centre of human pirin (Pang et al., 2004; Liu et al., 2013) are depicted by an asterisk below the sequence. The amino acids interacting with >50% of the binder proteins are denoted in blue bold-face font below the sequence. Orange bold-face font denotes binding interaction with <49% to >25% of binder proteins, and black regular font below the sequence represents amino acids binding <25% to >10% of binder proteins. The green font represents human pirin amino acids Arg14, Arg23, Glu32, and Lys34 interacting with nuclear factor NF-κB (Liu et al., 2013).

References

Liu F, Rehmani I, Esaki S, Fu R, Chen L, de Serrano V, Liu A. 2013. Pirin is an iron-dependent redox regulator of NF-κB. Proc Natl Acad Sci U S A. 110:9722-9727. doi: 10.1073/pnas.1221743110. PMID: 23716661.

Pang H, Bartlam M, Zeng Q, Miyatake H, Hisano T, Miki K, Wong LL, Gao GF, Rao Z. 2004. Crystal structure of human pirin: an iron-binding nuclear protein and transcription cofactor. J Biol Chem. 279:1491-1498. doi: 10.1074/jbc.M310022200. PMID: 14573596.

Supplemental File S3

Deduced peptide sequences of pTRG/genomic library clones interacting with Pir1.

pTRG clone number	Deduced Peptide Sequence	Homology to:	Locus-tag
1	DQLANGFHYTVGFNLSDYQSEVTKFDNESKELGNWYVGQKQGEI	TonB-dependent receptor	BF638R_3226
3	SPAPVGYYSTGSWLLFGRSYFVCLKSDCPYTYRDFEKTVFPNQEQI	Lipopolysaccharide kinase InaA family protein	BF638R_0173
6	DRTKEPGANGEPLYLDVKDCFYGAENAPVIVGGRYGLGSKDTTPAQIIAV FKNLAMPMPKNHFTIGIVDDVTFTSLPQEAEIALGGEGMFEAKFYGLGAD GTVGANKNSVKIIGDNTDKHCQAYFSYDSKKSGGFTCSHLRFGDDRSAKK FDHGILKFKWLQQIVDCIYLETLDGIFRIGGCKHNQGLHH	Pyruvate:ferredoxin (flavodoxin) oxidoreductase	BF638R_3194
11	DRLEDTQFFEEEIHAAVKAAEDAGTYVMVHVYVPRAIQRAIHAGVKSIEH GHLIDEPTMQLI	amidohydrolase family protein	BF638R_1629
12	DLLLMSEIKGYVNNPQYYFESRDDTHRKAI	Hypothetical protein	BF638R_1398
13	DRTKEPGANGEPLYLDVKDCFYGAENAPVIVGGRYGLGSKDTTPAQIIAV FKNLAMPMPKNHFTIGIVDDVTFTSLPQEAEIALGGEGMFEAKFYGLGAD GTVGANKNSVKIIGDNTDKHCQAYFSYDSKKSGGFTCSHLRFGDDRSAKK FDHGILKFKWLQQIVDCIYLETLDGIFRIGGCKHNQGLHH	Pyruvate:ferredoxin (flavodoxin) oxidoreductase	BF638R_3194
15	DRTKEPGANGEPLYLDVKDCFYGAENAPVIVGGRYGLGSKDTTPAQIIAV FKNLAMPMPKNHFTIGIVDDVTFTSLPQEAEIALGGEGMFEAKFYGLGAD GTVGANKNSVKIIGDNTDKHCQAYFSYDSKKSGGFTCSHLRFGDDRSAKK FDHGILKFKWLQQIVDCIYLETLDGIFRIGGCKHNQGLHH	Pyruvate:ferredoxin (flavodoxin) oxidoreductase	BF638R_3194
19	DLLLMSEIKGYVNNPQYYFESRDDTHRKAI	Hypothetical protein	BF638R_1398
21	SVGRYTVLRGRDSCCR	No significant similarity found	Non-canonical peptide
22	SINSTATPIASITASRRASGSPIRVTTSRL	No significant similarity found	Non-canonical peptide
24	TFCGECFFCRHGYVNNCTDPDGGWALGCRIDGGQAEYVRV	zinc-binding alcohol dehydrogenase	BF638R_1292
25	SFINVRNKRICQQSPILFRIPGRYTPQGNRSPI	No significant similarity found	Non-canonical peptide

Deduced peptide sequences of pTRG/genomic library clones interacting with Pir2. First experiment

pTRG clone number	Deduced Peptide Sequence	Homology to:	Locus-tag
1	SICILFLF	No significant similarity found	Non-canonical peptide
2	SICILFLF	No significant similarity found	Non-canonical peptide
3	TFCGECFFCRHGYVNNCTDPDGGWALGCRIDGGQAEYVRV	zinc-binding alcohol dehydrogenase	BF638R_1292
12	SFFILLNVFSA	No significant similarity found	Non-canonical peptide
18	SICILFLF	No significant similarity found	Non-canonical peptide
22	SFFILLNVFSA	No significant similarity found	Non-canonical peptide

Deduced peptide sequences of pTRG/genomic library clones interacting with Pir2. Second experiment

pTRG clone number	Deduced Peptide Sequence	Homology to:	Locus-tag
2	TFCGECFFCRHGYVNNCTDPDGGWALGCRIDGGQAEYVRV	zinc-binding alcohol dehydrogenase	BF638R_1292
14	SLVLLLSVKQW	No significant similarity found	Non-canonical peptide
20	TFCGECFFCRHGYVNNCTDPDGGWALGCRIDGGQAEYVRV	zinc-binding alcohol dehydrogenase	BF638R_1292
21	TFCGECFFCRHGYVNNCTDPDGGWALGCRIDGGQAEYVRV	zinc-binding alcohol dehydrogenase	BF638R_1292
31	TFCGECFFCRHGYVNNCTDPDGGWALGCRIDGGQAEYVRV	zinc-binding alcohol dehydrogenase	BF638R_1292

Supplemental file S3: Deduced amino acid sequences of Two-Hybrid System partial genomic library from *B. fragilis* 638R genome inserted in the pTRG plasmid interacting with pirin 1 (Pir1) or Pirin 2 (Pir2). The pTRG forward primer (Table S1) was used in the sequencing reactions to obtain nucleotide sequence of the inserted DNA cloned into the BamHI site downstream of the RNAP α-subunit.. Non-canonical peptide: in-frame translation of non-annotated DNA reading frame. The ligation of Sau3AI partial library into the pTRG BamHI site contains multiple random self-ligated Sau3AI DNA fragments. Only the deduced peptides expressed in-frame with RNA-α-subunit are shown.

Supplemental file S4

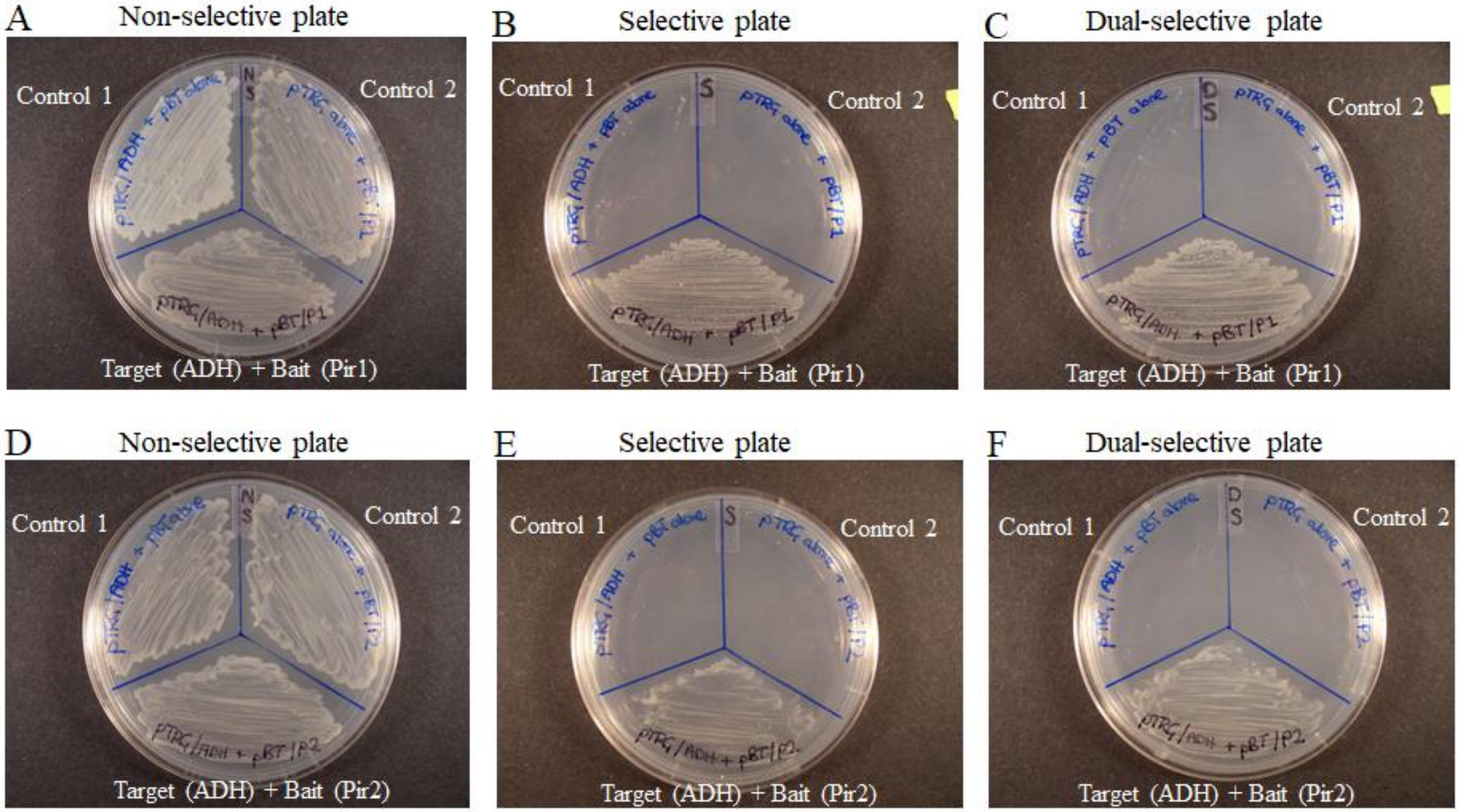

Supplemental file S4. Bacterial two-hybrid system assay showing protein-protein interaction between Pir1 and Zn-ADH (A, B, C), and between Pir2 and Zn-ADH (D, E, F). *E. coli* reporter strain was cotransformed with pBT/Pir1 (bait) and pTRG/ADH (prey) or pBT/Pir2 (bait) and pTRG/ADH (prey) constructs and they were grown on control non-selective plate (A, D), selective plate (B, E), and dual selective plate(C, F). Self-activation controls are *E. coli* two-hybrid system reporter strain carrying the following constructs: control 1, empty "bait" (pBT alone) cotransformed with loaded "prey" (pTRG/ADH); control 2, loaded "bait" (pBT/Pir1 or pBT/Pir2) cotransformed with empty "prey" (pTRG alone). See Materials and Methods for details on the bacterial two-hybrid system assay.

Supplemental File S6.

Growth conditions/strains	Short chain fatty acids concentration (mM)									
Anaerobic mid-log	Succinate	Lactate	Formate	Acetate	Propionate	Isobutyrate	Butyrate	Isovalerate	Phenylacetate	% Glucose used
638R	1.42 (0.71)	0.00 (0.00)	0.84 (0.75)	1.83 (0.53)	0.00 (0.00)	1.08 (1.08)	0.21 (0.21)	0.67 (0.21)	0.35 (0.01)	8.88 (2.06)
Δ pir1	1.30 (0.29)	0.06 (0.06)	0.79 (0.79)	0.93 (1.48)	0.51 (1.02)	1.56 (3.11)	0.22 (0.43)	0.07 (0.02)	0.00 (0.00)	8.30 (3.73)
Δ pir2	1.82 (0.25)	0.15 (0.30)	1.44 (1.21)	1.68 (1.59)	0.14 (0.13)	1.32 (2.63)	0.04 (0.08)	0.04 (0.07)	0.00 (0.00)	9.71 (4.37)
Δ pir1 Δ pir2	1.67 (0.9)	0.31 (0.61)	0.92 (1.15)	0.98 (1.3)	0.18 (0.36)	0.99 (1.96)	0.72 (0.54)	0.04 (0.06)	0.00 (0.00)	10.43 (0.14)
Anaerobic mid-log + DP	Succinate	Lactate	Formate	Acetate	Propionate	Isobutyrate	Butyrate	Isovalerate	Phenylacetate	% Glucose used
638R	1.12 (0.21)	0.32 (0.42)	1.81 (0.18)	0.87 (0.65)	1.14 (0.13)	0.00 (0.00)	0.00 (0.00)	0.32 (0.52)	0.00 (0.00)	8.19 (3.19)
Δ pir1	1.00 (0.02)	0.26 (0.34)	1.35 (2.70)	1.64 (1.00)	0.14 (0.27)	0.15 (0.29)	0.00 (0.00)	0.00 (0.00)	0.00 (0.00)	10.49 (3.13)
Δ pir2	1.14 (0.32)	0.42 (0.64)	1.29 (2.57)	1.24 (1.57)	0.02 (0.02)	2.28 (2.65)	0.00 (0.00)	0.03 (0.06)	0.00 (0.00)	8.92 (1.02)
Δ pir1 Δ pir2	0.86 (0.37)	0.29 (0.52)	0.92 (1.83)	0.78 (1.11)	0.25 (0.49)	2.16 (3.65)	0.47 (0.93)	0.00 (0.00)	0.00 (0.00)	7.41 (2.29)
Oxygen for 1h	Succinate	Lactate	Formate	Acetate	Propionate	Isobutyrate	Butyrate	Isovalerate	Phenylacetate	% Glucose used
638R	1.93 (0.91)	0.22 (0.43)	0.95 (0.98)	1.23 (1.40)	0.14 (0.28)	1.15 (2.29)	0.66 (0.53)	0.02 (0.02)	0.01 (0.03)	12.46 (2.74)
Δ pir1	1.81 (0.66)	0.23 (0.47)	0.85 (1.18)	1.17 (1.63)	0.00 (0.00)	0.87 (1.75)	0.20 (0.40)	0.05 (0.04)	0.01 (0.20)	12.16 (4.91)
Δ pir2	2.04 (0.16)	0.45 (0.32)	1.07 (1.46)	0.87 (1.09)	0.14 (0.28)	1.63 (3.24)	0.53 (0.93)	0.03 (0.04)	0.01 (0.02)	12.73 (4.73)
Δ pir1 Δ pir2	1.67 (0.53)	0.26 (0.17)	1.01 (0.76)	0.94 (1.88)	0.30 (0.60)	1.10 (2.07)	0.64 (0.26)	0.02 (0.02)	0.01 (0.03)	11.10 (5.16)
Anaerobic for 24 h	Succinate	Lactate	Formate	Acetate	Propionate	Isobutyrate	Butyrate	Isovalerate	Phenylacetate	% Glucose used
638R	14.01 (1.52)	3.50 (0.63)	6.00 (0.1)	15.38 (0.70)	3.57 (0.03)	0.94 (0.02)	0.00 (0.00)	0.29 (0.01)	0.14 0.00)	79.67 (0.35)
Δ pir1	13.71 (0.83)	4.40 (0.38)	6.07 (0.11)	15.36 (0.70)	3.48 (0.03)	0.97 (0.06)	0.00 (0.00)	0.30 (0.00)	0.14 (0.00)	79.56 (0.73)
Δ pir2	13.24 (1.13)	5.33 (0.88)	6.16 (0.10)	15.02 (0.84)	3.87 (0.71)	1.96 (2.02)	0.00 (0.00)	0.22 (0.12)	0.08 (0.11)	78.02 (1.76)
Δ pir1 Δ pir2	13.72 (1.00)	4.54 (0.41)	4.96 (0.58)	14.74 (0.06)	3.82 (0.75)	1.36 (0.75)	0.16 (0.31)	0.26 (0.07)	0.09 (0.11)	80.47 (0.22)
Oxygen for 24h	Succinate	Lactate	Formate	Acetate	Propionate	Isobutyrate	Butyrate	Isovalerate	Phenylacetate	% Glucose used
638R	3.31 (0.43)	9.92 (2.39)	0.00 (0.00)	2.88 (0.00)	1.65 (0.33)	0.82 (0.26)	0.00 (0.00)	0.00 (0.00)	0.05 (0.04)	26.20 (0.42)
Δ pir1	3.05 (0.75)	10.47 (2.22)	0.00 (0.00)	2.64 (1.66)	1.53 (1.84)	0.90 (0.82)	0.62 (0.45)	0.00 (0.00)	0.18 (0.26)	24.78 (3.06)
Δ pir2	3.02 (0.21)	11.17 (1.82)	0.00 (0.00)	3.15 (1.14)	1.36 (1.48)	0.67 (0.57)	0.00 (0.00)	0.34 (1.01)	0.15 (0.13)	24.41 (8.00)
Δ pir1 Δ pir2	2.72 (0.49)	10.90 (2.19)	0.00 (0.00)	2.69 (0.90)	1.15 (1.42)	1.15 (1.08)	0.63 (0.76)	0.00 (0.00)	0.17 (0.13)	28.02 (1.79)
Oxygen for 24h + DP	Succinate	Lactate	Formate	Acetate	Propionate	Isobutyrate	Butyrate	Isovalerate	Phenylacetate	% Glucose used
638R	2.21 (2.02)	2.62 (2.8)	0.12 (0.25)	2.62 (2.4)	0.00 (0.00)	0.04 (0.08)	0.00 (0.00)	0.00 (0.00)	0.00 (0.00)	11.46 (4.44)
Δ pir1	2.07 (1.95)	2.67 (2.00)	0.00 (0.00)	1.90 (1.23)	0.00 (0.00)	0.03 (0.06)	0.19 (0.39)	0.00 (0.00)	0.14 (0.24)	11.42 (4.57)
Δ pir2	2.23 (2.11)	2.34 (4.39)	0.00 (0.00)	2.46 (2.15)	0.00 (0.00)	0.32 (0.28)	0.11 (0.22)	0.00 (0.00)	0.07 (0.14)	9.44 (5.06)
Δ pir1 Δ pir2	2.18 (2.08)	2.93 (4.30)	0.00 (0.00)	2.54 (1.74)	0.00 (0.00)	0.13 (0.26)	0.37 (0.75)	0.00 (0.00)	0.00 (0.00)	10.14 (5.93)
Anaerobic for 48h	Succinate	Lactate	Formate	Acetate	Propionate	Isobutyrate	Butyrate	Isovalerate	Phenylacetate	% Glucose used
638R	14.49 (0.56)	3.60 (0.85)	6.04 (0.18)	15.73 (0.03)	3.86 (0.55)	0.90 (0.07)	0.00 (0.00)	0.29 (0.00)	0.15 (0.01)	79.47 (0.76)
Δ pir1	14.40 (0.57)	3.27 (2.63)	6.03 (0.03)	15.77 (0.11)	3.88 (0.83)	1.08 (0.27)	0.00 (0.00)	0.31 (0.01)	0.15 (0.01)	79.49 (0.58)
Δ pir2	14.21 (0.52)	3.87 (3.80)	6.37 (0.52)	15.89 (0.90)	4.10 (0.24)	2.25 (1.45)	0.04 (0.08)	0.16 (0.01)	0.04 (0.01)	78.46 (0.89)
Δ pir1 Δ pir2	14.27 (0.11)	3.39 (2.71)	5.24 (1.14)	14.66 (0.11)	4.26 (0.13)	1.78 (0.10)	0.16 (0.30)	0.18 (0.09)	0.03 (0.00)	80.56 (0.41)

Supplemental file S6. Short chain fatty acids concentrations (mM) in the culture supernatant of *B. fragilis* strains grown in PYG broth in conditions described in the panel, respectively. Anaerobic mid-log cultures were split, and one half was exposed to atmospheric air for 1h or 24h in aerobic shaker incubator at 37° C. After pelleting cultures, the clear supernatants were passed through 0.22 mm filters. Samples were analyzed for SCFAs using a BioRad HPLC organic acid system with AMINEX 87H, 300x7mm column with 5 mM sulfuric acid eluant at 0.6 ml/min, 65° C, with refractive index detector. Uninoculated media were used as blank and media background was subtracted except for glucose peak. DP: 2,2'-dipyridyl added at 50 μM. Values are means of two independent biological samples in mM. The range of the data points are shown inside parentheses.

Supplemental File S7

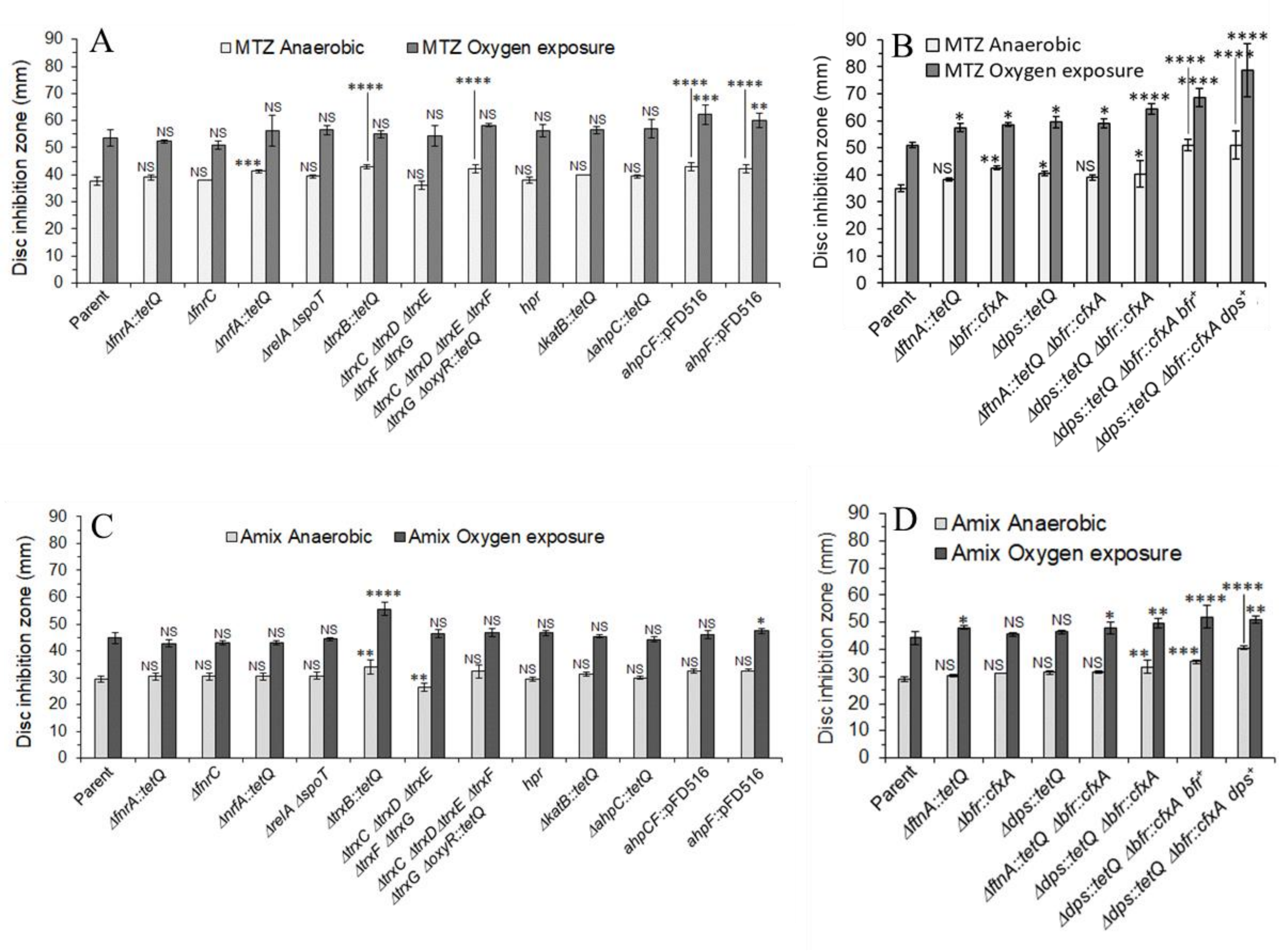

Supplemental file S7. Disc diffusion assay sensitivity for metronidazole (MTZ), panels A and B, and for Amixicile (Amix), panels C and D. Parent (*B. fragilis* 638R) and its isogenic mutant strains are depicted in each panel, respectively. Panels A and C) show mutant strains in the oxidative and redox stress responses, stringent response (*relA/spoT*), nitrite reductase (*nrfA*), and anaerobic regulators (*fnrA* and *fnrC*). Panels B and D) show mutants in the iron-storage family proteins *ftnA*, *bfr*, and *dps*, and genetic complemented strains, respectively. Strains designations are shown in each panel. Each bar represents the average zone inhibition (mm) of at least three independent biological replicates. Vertical error bars denote standard deviation of the means from two independent experiments in triplicate. The significance of the *P* value was calculated by one-way ANOVA followed by multiple comparisons using Dunnett and Bonferroni tests. Only groups with statistical significance in both tests are reported. $p<0.05$ (*), $p<0.01$ (**), $p<0.001$ (***), and $p<0.0001$ (****). NS: Not significant.

Supplemental File S8

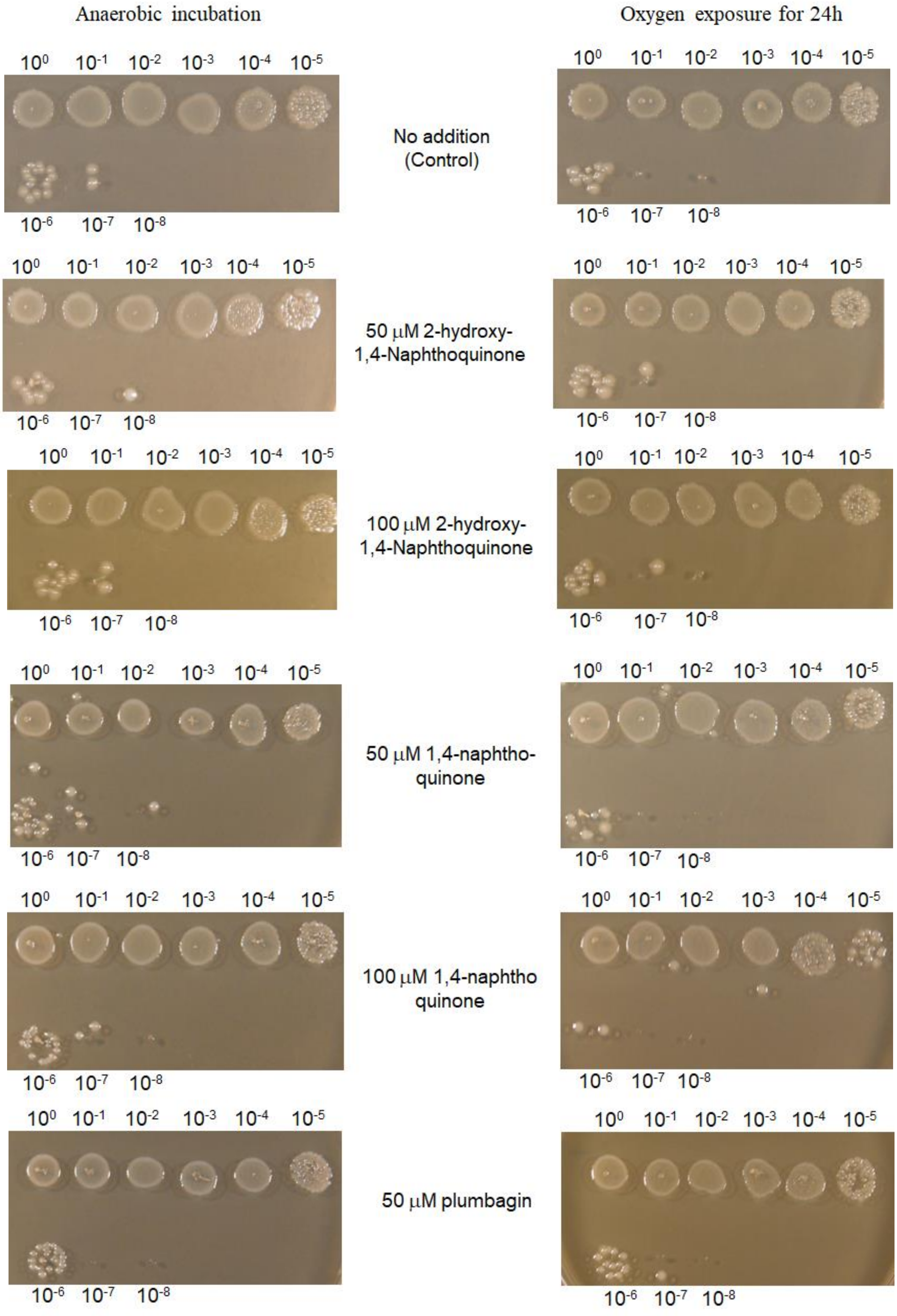

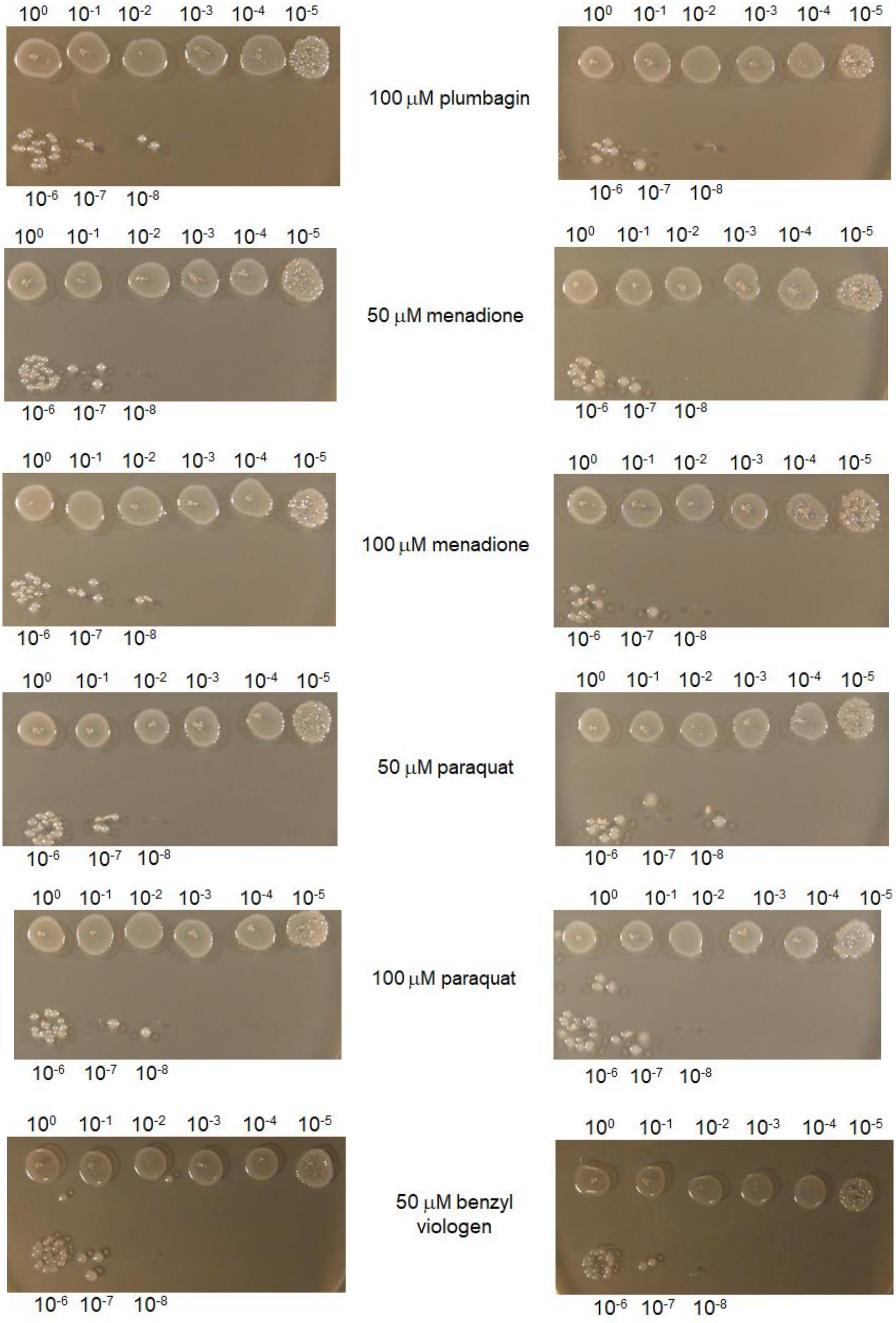

10^0 10^-1 10^-2 10^-3 10^-4 10^-5
10^-6 10^-7 10^-8
100 μM plumbagin
50 μM menadione
100 μM menadione
50 μM paraquat
100 μM paraquat
50 μM benzyl viologen

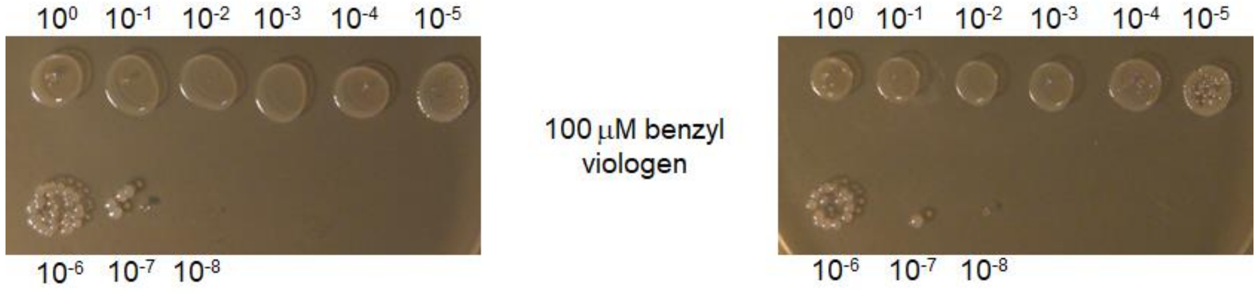

Supplemental file S8. Survival of *B. fragilis* 638R on BHIS supplemented with redox cycling agents as shown in each panel. Bacteria were grown in BHIS broth overnight and serially diluted in PBS, pH 7.4. Five µl aliquot from each dilution was place on top of the plate as indicated, respectively. Plates were inoculated in duplicated, and one set was immediately incubated in an anaerobic chamber incubator at 37° C (Anaerobic). The second set was placed in an aerobic incubator at 37° C for 20-24 h prior to incubation in an anaerobic chamber incubator (Oxygen exposure). Dilution factor is indicated above and below the panels.

Supplemental File S9

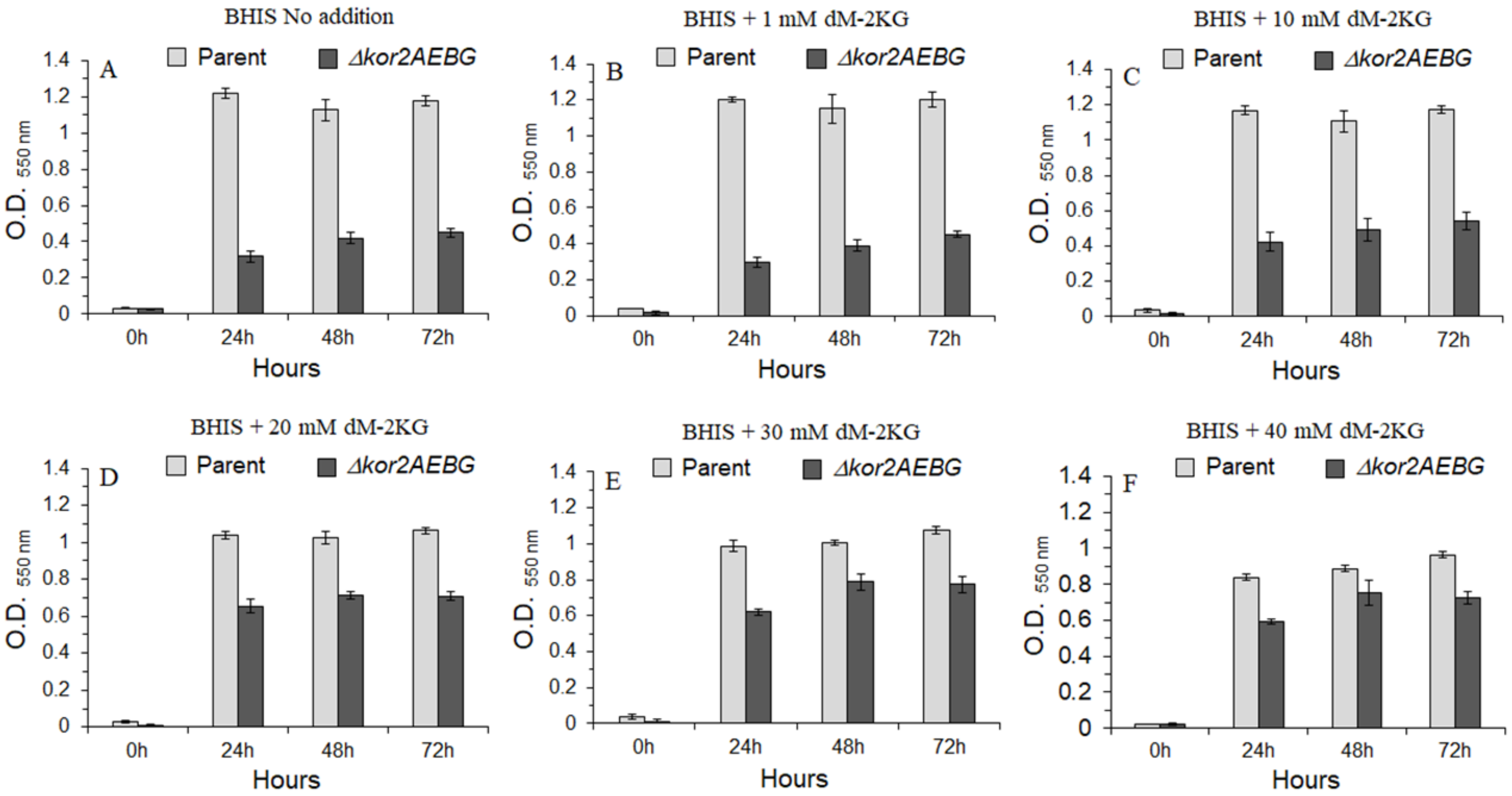

Supplemental file S9. Growth of *B. fragilis* 638R (parent) strain and the isogenic *Δkor2AEBG* mutant strain in supplemented brain-heart infusion (BHIS) media. Dimethyl-2-ketoglutarate (dM-2KG) was added as indicated in the panels. Strain designations are depicted for each panel. Media preparations and compositions are described in Materials and Methods section. Each bar represents the average of at least two independent experiments in triplicate. Vertical error bars denote standard deviation of the means.

Supplemental File S10

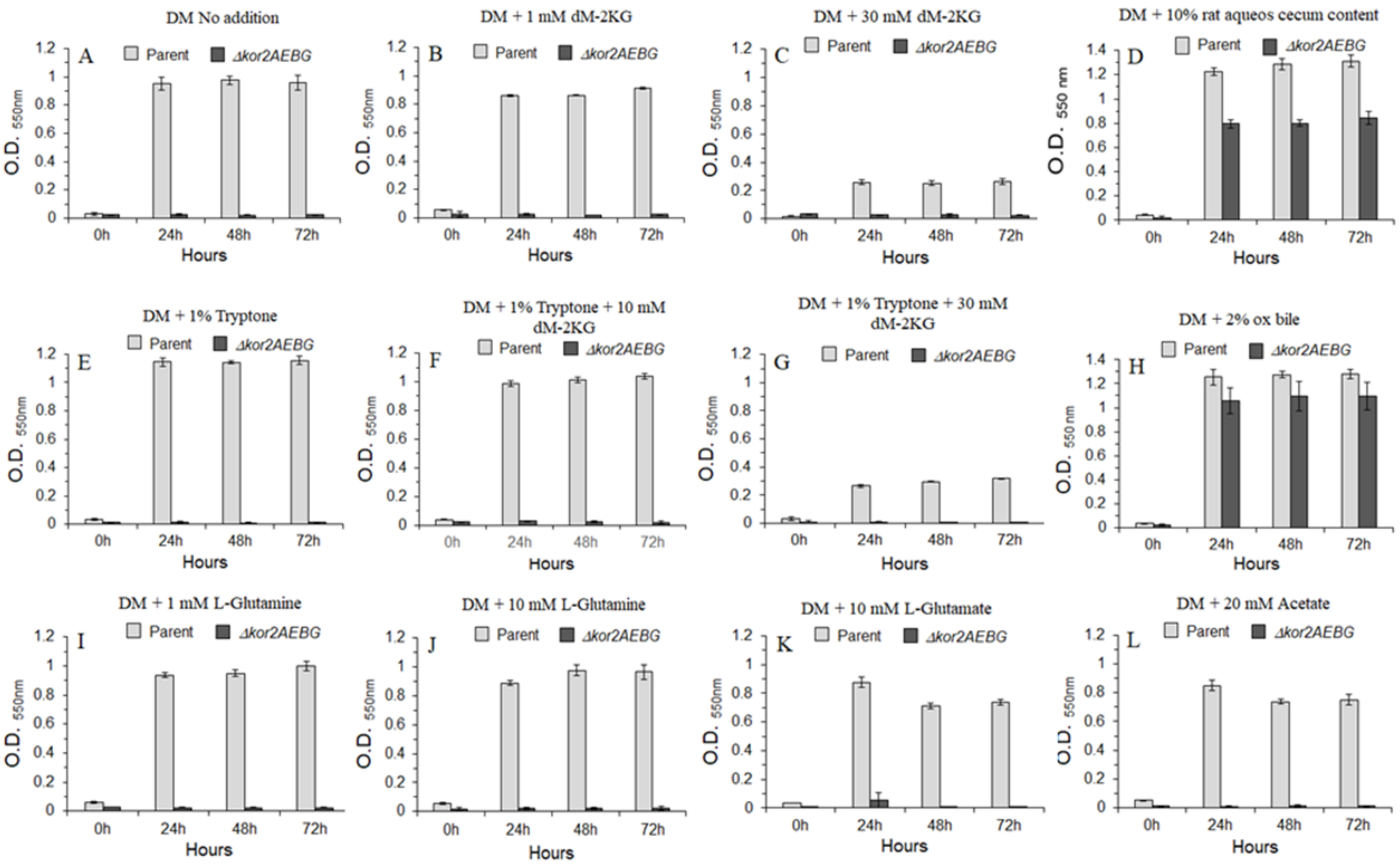

Supplemental file S10. Growth of *B. fragilis* 638R (parent) strain and the isogenic *Δkor2AEBG* mutant strain in chemically defined media with glucose (DM). DM was supplemented with 1% Tryptone, Dimethyl-2-ketoglutarate (dM-2KG), L-glutamine, L-glutamate, acetate, rat cell-free sterile aqueous cecum content, or ox bile as indicated in each panel. Strain designations are depicted for each panel. Media preparations and compositions are described in Materials and Methods section. Each bar represents the average of at least two independent experiments in triplicate. Vertical error bars denote standard deviation of the means.

Supplemental File S11

Supplemental file S11. Closantel minimal inhibitory concentration (MIC) for *Bacteroides* species in BHIS media.

Strains/Closantel MIC μg/ml (μM)	Anaerobic	O_2 exposure
B. fragilis 638R	8.6 (12.5)	8.6 (12.5)
B. fragilis BF8	4.3 (6.25)	4.3 (6.25)
B. fragilis ATCC 25285	4.3 (6.25)	4.3 (6.25)
B. thetaiotaomicron VPI 5482	274.4 (400)	34.3 (50)
B. vulgatus ATCC 8482	2.15 (3.12)	<0.01325 (<0.1953)

Supplemental File S12

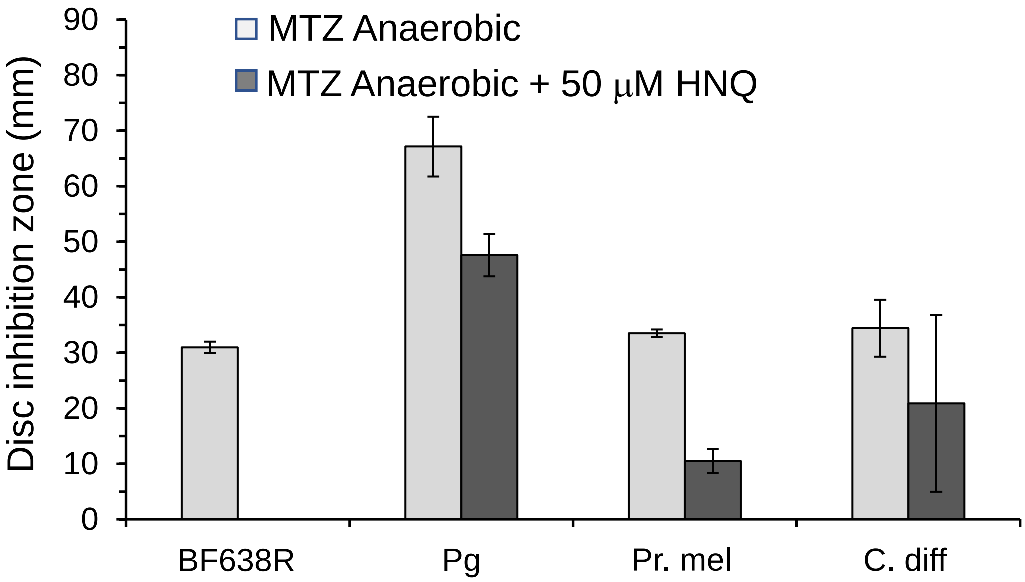

Supplemental file S12. Disc diffusion assays for MTZ susceptibility in the presence of 50 μM 2-hydroxy-1,4-naphthoquinone (HNQ). BF638R: *B. fragilis* 638R. Pg: *Porphyromonas gingivalis* ATCC 33277. Pr. mel: *Prevotella melaninogenica* ATCC 25845. *Clostridioides difficile* ATCC 43255. For this experiment BHI was supplemented with 5% defibrinated laked sheep blood. Each bar represents the average zone inhibition (mm) of at least three independent biological replicates. Vertical error bars denote standard deviation of the means from two independent experiments in triplicate.

Supplemental Table S1. List of oligonucleotide primers used in this study.

Primer name	Nucleotide Sequence
PIR1-pBT (EcoRI)	GAAAGGAATTCTATGAAAACAG
PIR1-pBT (BamHI)	GAATACGGATCCTTATCGCATTGG
PFOR-pTRG-FOR	GACTAAACAGAAGGGATCCATC
PFOR-pTRG-REV	GTTATTATCGGATCCAAGAT
PIR1-FOR-RTPCR	CAATCATGGTTGGCTGAAAA
PIR1-REV-RTPCR	GAATCCCATTTCAGGGTCAA
PIR1mut-BglII-FOR	GGACGCTTCAAGATCTCATTCC
PIR1mut-BamHI-REV	CCGACGGATCCAACTGTTACC
PIR1mut-SstI-FOR	GATACAAAGAGCTCTAAAGTGG
PIR1mut-SstI-REV	GTAGAGAAGAGCTCAGGTGCCG
PIR1-pFD-BamHI-FOR	CGTTTGGGATCCAGATGTTC
PIR1-pFD-SstI-REV	AAGGGCTGAGCTCGCTATTGAAGG
PIR2-pBT (EcoRI)	GAAAGGAATTCCATCATGAAA
PIR2-pBT (BamHI)	GCGGATCCTTTAAATTTACATCGG
ADH-pTRG-FOR	ATTAGGAGGATCCATAATGC
ADH-pTRG-REV	GTACTGAGGATCCCTACTCCGGG
PIR2-FOR-RTPCR	GGGTTCATTTCGGTGCTCT
PIR2-REV-RTPCR	CATGTGCCAGAAGACCCTTT
PIR2mut-BglII-FOR	CCCACCTGAAGATCTGTCGGAG
PIR2mut-BamHI-REV	CATATACGGATCCTCCGCGGG
PIR2mut-EcoRI-FOR	GGAGTCTACGAATTCCTGATCG
PIR2mut-EcoRI-REV	GGGATACTGAATTCGCGGCTG
PIR2-pFD-BamHI-FOR	GGTTTATAGGATCCATTTATAAAAG
PIR2-pFD-SstI-REV	CACTCCCGAGCTCGTTTCCGTG
pTRG-Forward	TGGCTGAACAACTGGAAGCT
pTRG-Reverse	ATTCGTCGCCCGCCATAA
pBT-Forward	TCCGTTGTGGGGAAAGTTATC
pBT-Reverse	GGGTAGCCAGCAGCATCC
Pyc-InsF	CGATGAATTCCGCAGGCAACTTTGTGCA
Pyc-InsR	CGATGGATCCAACGGACCGGGTAAAC
FNR_1017-NT-PstI	GACCTGCAGGCCATAAATGCTGCCATGGCC
FNR_1017-NT-SOE	CCACATCTCTGCAATACGTTCGGG
FNR_1017-CT-SOE	CCCGAACGTATTGCAGAGATGTGGCGACTGATCACCATCGACGGACG
FNR_1017-CT-XbaI	GCATCTAGAGAAGCAATGGCTGCTATTGC
FNR_1017-check-F	CTGTTATTCACGAAGTAAGCC

FNR_1017-check-R	GCAATGATTTGTACGTCCGG
Kor1-BamHI-FOR3	GAAAGTGGATCCAAACAAAAAACGC
Kor1-REV-SEW2	CTGCTCGGCGACTGCATCCGGAATCTGTGATGACCAGCC
Kor1-FOR-SEW	GGCTGGTCATCACAGATTCCGGATGCAGTCGCCGAGCAG
Kor1-SalI-REV	GATCTGTTTGTCGACAGTATGTGGTGC
Kor1-mutcheck-FOR	CACTAACACCGGTTCATGTTAATCC
Kor1-mutcheck-REV	GACTATGCGGTTACGACAGATTCG
Kor2-PstI-FOR	GATCGGGTTCACTGCAGATTAGCG
Kor2-REV-SEW	ACCGGGACTTGATGATGAGG
Kor2-FOR-SEW	CCTCATCATCAAGTCCCGGTCCGAACGCCACCATCACC
Kor2-BamHI-REV	GACTGAGTGTTACTGATGGATCCGCG
Kor2-mutcheck-FOR	GTAATATCCCTCGCCAAAGAGG
Kor2-mutcheck-REV	GGCTTTCGCTGACGTGCC
Kor2comp-BamHI-FOR	GATCGGATCCGCAACCGTATGTCCGGACGG
Kor2comp-SacI-REV	GATCGAGCTCCCGCTATCTCTGCCTGTGGC
PoxB-BamHI-FOR	GATCGGATCCGACGGATCTGCTCCATCATCCG
PoxB-REV-SEW	GGACCTTCCAGCTCGAAAGCGCCGGTCAGTTGCGCTTCTGC
PoxB-FOR-SEW	GCAGAAGCGCAACTGACCGGCGCTTTCGAGCTGGAAGGTCC
PoxB-PstI-REV	GATCCTGCAGGGAATCGGATCGACAGACCG
PoxB-mutcheck-FOR	TTTCCGGCATTCAAGCCG
PoxB-mutcheck-REV	CGCGAATGAAAGGTTTGAGG
Acn-BamHI-FOR	GATCGGATCCACAATTCTGTTTCCGGGTTGG
Acn-SEW-REV	GCCAAGTCACGTGCGAGTTCCCTTCCATCTTCCGGGGATAACC
CitS-SEW-FOR	GGTTATCCCCGGAAGATGGAAGGGAACTCGCACGTGACTTGGC
CitS-SalI-REV	GATCGTCGACCCCAAACTCTTCCCGATAGCG
Acn-mutcheck-FOR	GGTCCGTTTACTCCGGACGC
CitS-mutcheck-REV	TCTGTGCAATCTGTAAATCTGCGG
AcnBglIIcompl-FOR	GATCAGATCTAATAAATAGAAGAACAGATAAAACCGAAA
CitSacIcompl-REV	GATCGAGCTCCCACCACAGATTGGCAGACGG
FrdC-BamHI-FOR	GATCGGATCCGCACAATGAGAAAGAAGGCTCG
FrdC-BamHI-REV	GATCGGATCCGGCAAGTCCGGTACCTACAACG
FrdB-BamHI-FOR	GATCGGATCCCCGGTGAAAATTCAGAACCGG
FrdB-SacI-REV	GATCGAGCTCGTTCCTTGCGAGCCTGACGG

The underlined nucleotide sequences denote restriction sites used for cloning into appropriate plasmid vector restriction sites respectively.
